# App-Based Physical Activity Intervention Among Women With Prior Hypertensive Pregnancy Disorder: A Randomized Clinical Trial

**DOI:** 10.1001/jamanetworkopen.2025.2656

**Published:** 2025-04-02

**Authors:** Lili L. Kókai, Diarmaid Ó Ceallaigh, Anne I. Wijtzes, Jeanine E. Roeters van Lennep, Johannes J. Duvekot, Martin S. Hagger, John Cawley, Alex Burdorf, Kirsten I. M. Rohde, Hans van Kippersluis

**Affiliations:** 1 Department of Public Health, Erasmus MC University Medical Center, Rotterdam, the Netherlands; 2 Economic and Social Research Institute, Dublin, Ireland; 3 IDEA Center, Erasmus University Rotterdam, Rotterdam, the Netherlands; 4 Erasmus School of Social and Behavioural Sciences, Erasmus University Rotterdam, Rotterdam, the Netherlands; 5 Department of Internal Medicine, Erasmus MC University Medical Center, Rotterdam, the Netherlands; 6 Department of Obstetrics and Gynecology, Erasmus MC University Medical Center, Rotterdam, the Netherlands; 7 Department of Psychological Sciences, University of California, Merced; 8 Faculty of Sport and Health Sciences, University of Jyväskylä, Jyväskylä, Finland; 9 Cornell Jeb E. Brooks School of Public Policy, Cornell University, Ithaca, New York; 10 School of Business and Economics, Maastricht University, Maastricht, the Netherlands; 11 Tinbergen Institute, Erasmus University Rotterdam, Rotterdam, the Netherlands; 12 Erasmus School of Economics, Erasmus University Rotterdam, the Netherlands; 13 Erasmus Research Institute of Management, Erasmus University Rotterdam, the Netherlands

## Abstract

**Importance:**

Insufficient moderate to vigorous physical activity (MVPA) is a risk factor for cardiovascular disease (CVD). Effective interventions are needed to bridge the intention-behavior gap and increase MVPA, especially among women with prior hypertensive pregnancy disorder (HPD).

**Objective:**

To test the effectiveness of two 8-week app-based MVPA interventions (motivation and action) that were based on the integrated behavior change (IBC) model and used evidence-based behavior change techniques from behavioral sciences.

**Design, Setting, and Participants:**

This randomized clinical trial (RCT) included women with prior HPD. A purpose-built app was tested from October 2021 to March 2022, with follow-up immediately after the intervention (week 9), 3 months later (week 21), and 12 months later (week 61). The study ended in May 2023. Data were analyzed from March 31, 2022, to June 9, 2024.

**Intervention:**

All participants received a wearable fitness tracker and a purpose-built physical activity intervention app and were randomized to 1 of 3 groups (control, motivation, or action): The control group received information on CVD, MVPA, and HPD; the motivation group received the same information as well as motivational interviewing–based counseling; and the action group received the same information as well as behavior change techniques that targeted all processes in the IBC model (motivational, volitional, automatic): motivational interviewing–based counseling, action and coping planning, commitment, positive psychology, and mindfulness-based stress reduction.

**Main Outcomes and Measures:**

The primary outcome was MVPA (in minutes per week). Treatment effects were estimated using available case ordinary least-squares regression.

**Results:**

A total of 619 women participated in this study: 205 in the control group, 209 in the motivation group, and 205 in the action group. Their mean (SD) age was 38.9 (7.3) years; 386 of 577 participants (67%) had a bachelor’s degree or more and 550 of 577 (95%) were living with a child or children. The mean (SD) weekly MVPA for all 3 groups went from a high baseline of 242 (190) minutes to 197 (208) minutes immediately post intervention. No significant postintervention treatment effects on MVPA were observed for the action group (week 9 treatment effect, −17 [95% CI, −58 to 23] min/wk) or the motivation group (week 9 treatment effect, −3 [95% CI, −58 to 51] min/wk), despite the action intervention positively influencing motivational and volitional processes. The app and intervention components were all evaluated positively by participants.

**Conclusions and Relevance:**

In this clinical trial of 2 app-based MVPA interventions among 619 women with prior HPD, no treatment effects on MVPA were observed. Possible explanations include (1) the importance of automatic processes in determining MVPA and the absence of treatment effects on these processes and (2) the high baseline activity levels of control group participants, which may have given less room for the treatments to improve upon. These are important considerations for those designing future MVPA interventions and RCTs.

**Trial Registration:**

Netherlands Trial Register Identifier: NL9329

## Key Points

**Question:**

Can theory- and evidence-based behavior change apps help women with a history of hypertensive pregnancy to increase their physical activity?

**Findings:**

In this randomized clinical trial of 619 participants, 2 app-based physical activity interventions (motivation and action) were tested. Participants in both treatment groups and the control group had high activity levels at baseline, and no treatment effects on physical activity were found.

**Meaning:**

Despite high initial activity levels across all groups, treatment groups receiving behavior change techniques did not sustain activity levels better than the control group.

## Teaser

This randomized clinical trial evaluates the effectiveness of 2 motivation and action app-based interventions in increasing moderate to vigorous physical activity levels among women with prior hypertensive pregnancy disorder.

## Introduction

The World Health Organization (WHO) recommends that adults (aged 18-64 years) engage in at least 150 minutes of moderate physical activity or 75 minutes of vigorous physical activity each week.^1^ A quarter of people worldwide fail to meet these guidelines.^2^ Insufficient moderate to vigorous physical activity (MVPA) can have severe consequences for physical and mental health, causing 9% of premature deaths.^3^ Women with prior hypertensive pregnancy disorder (HPD) have an increased risk of cardiovascular disease (CVD) and therefore may particularly benefit from, and be more motivated to engage in, MVPA interventions.^4,5,6^

Many behavior change interventions have focused on MVPA. Although many such interventions have been successful in producing small, short-term effects, evidence of large, long-term effects is scant.^7,8,9,10^ Health behavior change interventions may have better and less variable effects when solidly rooted in theory.^11,12,13,14,15^ Approaches based on traditional social cognition theories, which describe deliberative psychological processes only, often succeed in influencing behavioral intentions but not actual behavior.^16,17,18,19^ To bridge this gap between intentions and behavior, both deliberative and automatic processes should be targeted.^20^ To this end, the integrated behavior change (IBC) model describes the motivational, volitional, and automatic processes that govern behavior.^21^ Observational studies show that this theory predicts several health behaviors, including MVPA.^22,23,24,25,26,27^

To our knowledge, this randomized clinical trial (RCT) among women with prior HPD is the first to apply the IBC model. Our objective was to test the effectiveness of MVPA interventions designed to target the 3 IBC model processes based on insights from behavioral sciences. Participants were randomized to 1 of 3 groups: The control group received information on CVD, MVPA, and HPD; the motivation group additionally received an intervention targeting motivational processes; and the action group also received information as well as an intervention that targeted all 3 IBC model processes. These interventions were delivered over 8 weeks via a purpose-built physical activity intervention app. The primary outcome, MVPA, was measured using a wearable fitness tracker.

## Methods

The Medical Ethics Committee of Erasmus MC University Medical Center (hereinafter, Erasmus MC) approved this RCT. Details of the experimental design are presented in the trial protocol in Supplement 1.^28^ This study followed the Consolidated Standards of Reporting Trials (CONSORT) reporting guideline.

### Participant Recruitment and Randomization

We aimed to recruit 630 participants (n = 210 per group) for sufficient statistical power (α = .05, power = 0.8) to detect a small-to-medium MVPA effect size.^28^ Email invitations were sent to more than 1200 patients of the Follow-Up Pre-Eclampsia Outpatient Clinic (FUPEC) at Erasmus MC in Rotterdam, the Netherlands,^29^ and to more than 900 other Erasmus MC patients with prior HPD. Additionally, the study was promoted by the HELLP Foundation, the Dutch patient organization for women with HELLP (hemolysis, elevated liver enzymes, low platelet count) syndrome, preeclampsia, or both. Recruitment began in October 2021 and closed in March 2022, with 663 participants (540 FUPEC patients, 73 non-FUPEC Erasmus MC patients, and 50 women recruited through the HELLP Foundation). The inclusion criterion was having prior HPD. Individuals were excluded if they were aged younger than 18 years, were pregnant, were less than 3 months post partum, had physical limitations preventing MVPA, were unable to speak Dutch or English, or did not own a smartphone. Eligible participants were enrolled online by Avegen, the developer of the purpose-built physical activity intervention app, i2be, after they provided informed consent. Participants received a wearable fitness tracker (Fitbit Inspire 2; Fitbit Inc) and a link to download the app.

After participants logged into the physical activity intervention app, they were automatically randomized in-app to 1 of 3 groups: control, motivation, or action (allocation ratio 1:1:1). Permuted block randomization was used (variable block sizes of 6 or 9), stratified on self-reported prior-month MVPA and being less than 12 months post partum. Allocation was concealed from participants, and they were not informed about the intervention content of the other groups. The app developers held the allocation data until after the first postintervention end point and so the researchers remained blinded to allocation during the intervention.

### Experimental Design

Patient involvement in the design included getting FUPEC patient input through 2 qualitative studies.^30,31^ Patients expressed a need for mHealth MVPA interventions, preferably incorporating health metric tracking, interactivity, behavior change strategies, information provision, and personalization—all of which were incorporated in our interventions. The factors that patients identified as determining their MVPA aligned closely with the IBC model’s 3 processes, supporting our use of this model.

#### Study Timeline

Two weeks after logging in to the app, participants’ sociodemographic data and baseline outcome measures were collected via the app. Then an 8-week intervention was delivered through the app. The primary end points for analysis were week 9 (first week post intervention), week 21 (3-month follow-up), and week 61 (12-month follow-up). Additionally, MVPA was measured in week 0 (the baseline or preintervention week) and weekly during the intervention, and MVPA was assessed at the intervention midpoint (week 5) (eFigure 1 in Supplement 2). The study started in October 2021 and ended in May 2023.

#### Intervention Groups

The interventions consisted of behavior change techniques (BCTs) from behavioral sciences (psychology and behavioral economics) delivered in-app. The techniques were systematically selected based on the IBC model, guided by evidence on the relationship between the techniques and theoretical variables featured in the IBC model^32,33^ and by empirical evidence on the effectiveness of the techniques in spurring MVPA change.^34,35,36,37,38,39,40^

The interventions were packaged in weekly modules. The motivation group received the Get Motivated module, targeting the motivational processes of intrinsic motivation and intention using content-based motivational interviewing techniques.^41^ The action group received the Get Motivated, Get Activated, and Get Energized modules. The Get Activated module targeted volitional processes using action planning, coping planning, and commitment^42,43^ and used additional personalized features including self-set MVPA goals, the option to commit to them, and tailored action plan reminders. The Get Energized module targeted the automatic processes of affect and stress using mindfulness-based stress reduction and positive psychology.^38,44^ Further details are provided in eFigures 2 and 3 in Supplement 2.^45^

All 3 groups received a weekly information module, Get Informed, providing information on MVPA, HPD, and CVD risk. Participants could self-monitor in-app their weekly MVPA and resting heart rate. App engagement was encouraged through gamification elements. Participants earned app points for completing modules, which yielded virtual and real rewards (eg, entry into weekly raffles for €25 sports vouchers).^46^ The modules did not prescribe exercise routines: it was left to participants to decide how to accumulate MVPA minutes. The eAppendix in Supplement 2 provides more detail on the app and the implementation of the BCTs.

#### Outcome and Control Variables

The primary outcome, MVPA (in minutes per week), was measured with a wearable fitness tracker, meaning all types of MVPA were tracked (eg, running, swimming, strength training).^47^ Secondary outcome measures were weekly mean wearable fitness tracker–measured daily resting heart rate^48^ and self-reported measures such as body mass index (BMI; calculated as weight in kilograms divided by height in meters squared), waist-to-hip ratio, cardiorespiratory fitness level, and subjective well-being.^49,50,51,52^

Tertiary outcomes were the psychological variables that the BCTs targeted (measured with Likert scales). These included the motivational process variables intrinsic motivation and intention; the volitional process variables action planning, coping planning, and commitment; and the automatic process variables affect and stress (eFigures 2 and 3 in Supplement 2).^53,54,55,56,57^ Control variables were baseline MVPA and self-reported age, trait self-control, habit, household composition, educational level, and type of prior HPD (eTable 1 in Supplement 2).^58,59^ All data were collected via the physical activity intervention app.

### Statistical Analysis

The analysis plan was preregistered with the protocol.^28^ Within-individual change in outcome variables was analyzed for descriptive purposes. For the primary analysis, the estimand was the adjusted mean difference in MVPA in a given week between the action intervention and the control intervention, regardless of the level of engagement with the app modules or any postrandomization changes in physical capacity for MVPA (eg, pregnancy), in women with prior HPD, excluding those with missing MVPA data in the relevant week (ie, available case analysis).^60^ The estimand was calculated using ordinary least-squares (OLS) regression with control variables, and a separate estimand was calculated with separate regression models for each of weeks 5, 9, 21, and 61. OLS regression and its underlying assumptions were deemed appropriate (eMethods in Supplement 2).

In a similar manner, we also calculated estimands for the treatment effect on MVPA of the action intervention relative to the motivation intervention and of the motivation intervention relative to the control. Although these were not our primary estimands, the motivation intervention was included in our study, and these 2 additional estimands were calculated to determine whether targeting all 3 IBC processes was more effective than targeting motivational processes alone.

Subgroup analyses were performed based on educational level and baseline MVPA (baseline MVPA analysis was exploratory, as it was not preregistered). Sensitivity analyses were performed on a per-protocol basis, including only compliant participants (ie, completed at least 75% of each module available to them in at least 7 weeks of the 8-week intervention). Several other sensitivity analyses were also performed: one excluded participants who were not FUPEC patients, another excluded those who became pregnant during the trial, and several used imputation methods (multiple imputation by chained equations, best-worst analysis, and worst-best analysis).^61,62^

A process evaluation examined program fidelity (ie, compliance, time spent on modules) and program acceptability (ie, easy to use, stimulating for MVPA, helpful in attaining MVPA goals, and appealing, evaluated on a 5-point Likert scale). Statistical significance was determined at *P* < .05 (2-tailed). Data analysis was conducted using Stata, version 18 (StataCorp). Data were analyzed from March 31, 2022, to June 9, 2024.

## Results

### Sample Characteristics

This study included 619 women (205 in the control group, 209 in the motivation group, and 205 in the action group), with a mean (SD) age of 38.9 (7.3) years. The Table presents baseline characteristics for the full sample. A total of 386 of 577 participants (67%) had a bachelor’s degree or more, and 550 of 577 (95%) were living with a child or children. At week 0, the 481 participants for whom we had week 0 MVPA data engaged in MVPA for a mean (SD) of 235 (191) minutes. The mean (SD) resting heart rate was 66.7 (7.4) bpm (normal range).^63^ Participants had a mean (SD) BMI of 26.4 (5.4) (overweight) and a mean (SD) waist-to-hip ratio of 0.86 (0.11) (increased risk of disease).^51,52^ Participants had a mean (SD) cardiorespiratory fitness level of 28.7 (23.5) mL/kg/min, which was below the median for Dutch women aged 20 to 50 years.^64^ Finally, participants had a mean (SD) subjective well-being score of 5.0 (1.1) (scale, 1-7).

**Table.** Baseline Characteristics of Participants^a^

Characteristic	Full sample	Intervention group			No. of participants with nonmissing data^b^
		Control	Motivation	Action	
Educational level					
Primary education at most	15 (3)	7 (4)	3 (2)	5 (3)	577
More than primary education but less than a bachelor’s degree	176 (31)	53 (28)	61 (32)	62 (33)	
Bachelor’s degree or more	386 (67)	132 (69)	132 (66)	122 (65)	
Household composition					
Living with partner	489 (85)	160 (83)	170 (87)	159 (84)	577
Living with a child or children	550 (95)	185 (96)	188 (96)	177 (94)	
Type of prior HPD					
Prior preeclampsia or eclampsia	379 (69)	132 (73)	122 (65)	125 (69)	549
Prior HELLP syndrome	306 (56)	101 (56)	108 (57)	97 (54)	
Other prior HPD	164 (30)	47 (26)	60 (32)	57 (32)	
Lactating	39 (7)	7 (4)	19 (10)	13 (7)	551
<12 mo Post partum	92 (15)	30 (15)	31 (15)	31 (16)	610
Age, mean (SD), y	38.9 (7.3)	39.0 (7.3)	39.0 (7.2)	38.6 (7.6)	577
MVPA at week 0 (measured via a wearable fitness tracker), mean (SD), min/wk	235 (191)	227 (174)	261 (238)	218 (152)	481
Resting heart rate, mean (SD), bpm	66.7 (7.4)	66.5 (7.4)	67.2 (8.0)	66.3 (6.7)	467
BMI, mean (SD)	26.4 (5.4)	26.7 (5.1)	26.5 (5.8)	26.1 (5.2)	555
Waist-to-hip ratio, mean (SD)	0.86 (0.11)	0.86 (0.12)	0.86 (0.12)	0.85 (0.07)	486
Cardiorespiratory fitness level, mean (SD), mL/kg/min	28.7 (23.5)	28.4 (25.0)	31.6 (21.8)	25.7 (23.6)	336
Subjective well-being score (scale, 1-7), mean (SD)	5.0 (1.1)	4.9 (1.1)	5.1 (1.2)	5.0 (1.1)	512
Trait self-control score (scale, 1-5), mean (SD)	3.2 (0.7)	3.1 (0.7)	3.2 (0.7)	3.2 (0.6)	510
Habit score (scale, 1-6), mean (SD)	3.5 (0.7)	3.5 (0.7)	3.5 (0.8)	3.5 (0.7)	472

Abbreviations: BMI, body mass index (calculated as weight in kilograms divided by height in meters squared); HELLP, hemolysis, elevated liver enzymes, low platelet count; HPD, hypertensive pregnancy disorder; MVPA, moderate to vigorous physical activity.

^a^ Unless otherwise indicated, values are presented as No. (%) of participants.

^b^ Total number of participants for which a value for the relevant variable was recorded (ie, is nonmissing).

### Attrition

**Figure 1.**
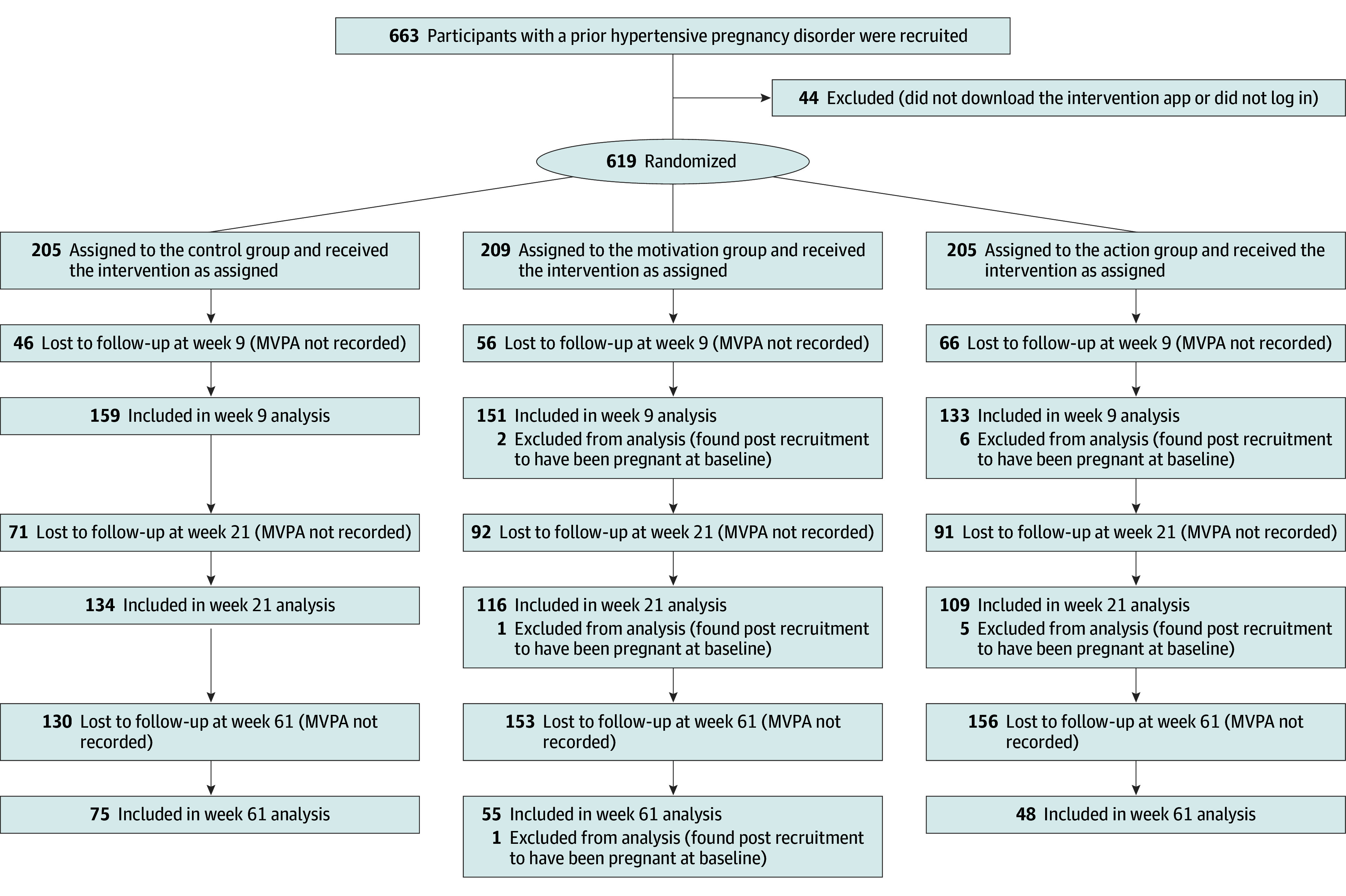
Participant Recruitment Flow The primary end points for analysis were week 9 (first week post intervention), week 21 (3-month follow-up), and week 61 (12-month follow-up). MVPA indicates moderate to vigorous physical activity.

Overall attrition was 27% by week 9 (168 participants lost to follow-up), 41% by week 21 (254 participants lost to follow-up), and 71% by week 61 (439 participants lost to follow-up) (Figure 1). Attrition rates immediately post intervention were 22% (n = 46) for the control group, 27% (n = 56) for the motivation group, and 32% (n = 66) for the action group. However, attrition was neither associated with observable personal characteristics nor significantly different across groups (eTables 2-7 in Supplement 2). The week 9 and week 21 attrition rates were in the same range as reported in previous meta-analyses (20%-40%).^65,66^ The final sample size at week 9 was 443 vs the protocol estimate of 504.

### Primary Outcome

#### Within-Individual Changes in MVPA

**Figure 2.**
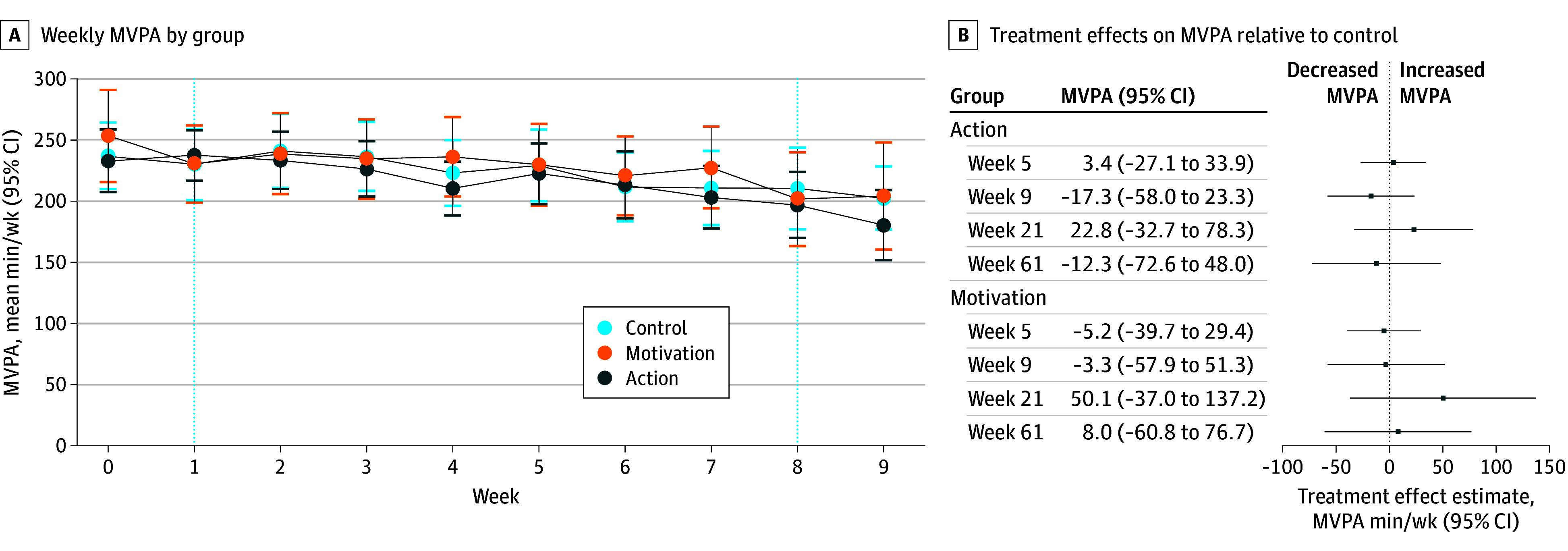
Within-Individual Mean Changes in and Treatment Effects on Moderate to Vigorous Physical Activity (MVPA) A, Mean weekly MVPA values are presented with 95% CIs. The sample includes participants for whom MVPA data for each of weeks 0 to 9 were nonmissing (n = 435). Vertical dashed lines indicate the first week (week 1) and the final week (week 8) of the intervention period. B, Treatment effect estimates are presented with 95% CIs. Ordinary least-squares regression was used to determine treatment effects of MVPA at weeks 5 (n = 471), 9 (n = 443), 21 (n = 359), and 61 (n = 178) on indicators for being in the motivation and action groups. Control variables were baseline MVPA, age, trait self-control, habit, household composition, educational level, and type of prior hypertensive pregnancy disorder.

Among the 435 participants for whom we had both week 0 (baseline) and week 9 MVPA data, mean (SD) MVPA was 242 (190) minutes at week 0 but declined in subsequent weeks, reaching 197 (208) minutes by week 9 (Figure 2A). This trend was similar across groups. Unadjusted *t* tests (not preregistered) showed that mean MVPA was significantly lower at week 9 than at week 0 (difference, 45 [95% CI, 24-65] minutes). However, mean (SD) MVPA at week 9 remained well above the WHO-recommended minimum of 150 min/wk. A total of 289 of 435 participants (66%) exceeded 150 minutes in week 0, with 222 (51%) doing so at week 9. Between weeks 9 and 21, MVPA was relatively stable among participants who remained in the study, with decreases observed by week 61 (eFigures 4 and 5 in Supplement 1).

#### Treatment Effects on MVPA

Estimated treatment effects on MVPA at weeks 5, 9, 21, and 61 showed no significant differences for the action group (week 9 treatment effect, −17 [95% CI, −58 to 23] min/wk) or motivation group (week 9 treatment effect, −3 [95% CI, −58 to 51] min/wk) compared with the control group (Figure 2B). Subgroup analysis showed a significant positive interaction at weeks 5 and 9 between the action group and having below-median baseline MVPA, indicating that the action intervention worked better for those with low baseline MVPA than those with high baseline MVPA (interaction effect, 74 [95% CI, 8 to 140] min/wk at week 5 and 86 [95% CI, 11 to 162] min/wk at week 9) (eFigure 6 in Supplement 2). This week 5 finding was robust to running the interaction analysis on an intention-to-treat basis, rather than available case, but the week 9 finding decreased to being only significant at the 10% level (interaction effect, 70 [95% CI, −10 to 150] min/wk) (eFigure 7 in Supplement 2). Neither interaction remained significant when subjected to a multiple testing correction.

There were no differences in treatment effects between educational level subgroups, and none of the sensitivity analyses yielded any significant results. No significant differences were found between the action and motivation groups.

### Secondary Outcomes

**Figure 3.**
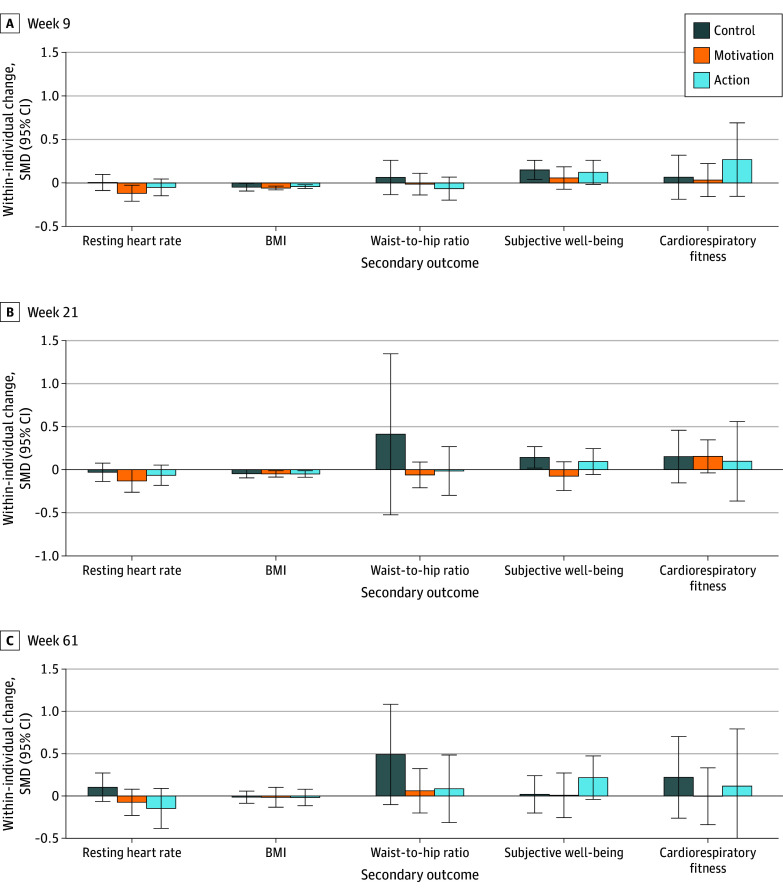
Within-Individual Mean Changes in Secondary Outcomes A to C, Standardized mean differences (SMDs) with 95% CIs from baseline (week 0) at each of weeks 9 (A), 21 (B), and 61 (C). In each case, the sample is all participants for whom the relevant variable is observed both at week 0 and the relevant end-point week. Secondary outcomes included resting heart rate (week 9, n = 377; week 21, n = 271; and week 61, n = 132); body mass index (BMI; measured in weight in kilograms divided by height in meters squared) (week 9, n = 332; week 21, n = 206; and week 61, n = 131), waist-to-hip ratio (week 9, n = 265; week 21, n = 150; and week 61, n = 83), subjective well-being (week 9, n = 283; week 21, n = 177; and week 61, n = 94), and cardiorespiratory fitness level (week 9, n = 165; week 21, n = 93; and week 61, n = 53).

Figure 3 illustrates mean within-individual changes in secondary outcomes. In general, we observed improvements in most outcomes across the 3 groups. We do not report hypothesis testing for these outcomes, due to the absence of treatment effects for the primary outcome (MVPA).

### Tertiary Outcomes: Treatment Effects on Psychological Process Variables

**Figure 4.**
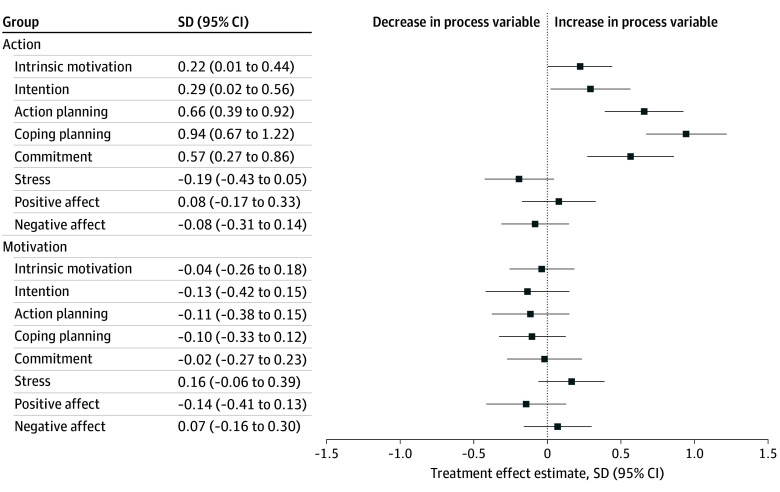
Treatment Effects on Psychological Process Variables at Week 9 Ordinary least-squares regressions of integrated behavior change (IBC) model process variables (standardized) at week 9 on indicators for being in the motivation and action groups. Control variables were baseline moderate to vigorous physical activity, age, trait self-control, habit, household composition, educational level, and type of prior hypertensive pregnancy disorder. Treatment effect estimates are presented with 95% CIs. The sample size for each regression included all those for whom the relevant IBC model process variable was not missing. Variables included intrinsic motivation (n = 295), intention (n = 306), action planning (n = 302), coping planning (n = 297), commitment (n = 307), stress (n = 289), positive affect (n = 293), and negative affect (n = 303).

Figure 4 shows that the action intervention significantly enhanced motivational and volitional process variables at week 9 compared with the control. For example, action group participants demonstrated a 0.9-SD (95% CI, 0.7-1.2) higher increase in coping planning, a 0.7-SD (95% CI, 0.4-0.9) higher increase in action planning, and a 0.6-SD (95% CI, 0.3-0.9) higher increase in commitment. However, the effects of the action intervention diminished by week 21, with only intrinsic motivation showing a significant effect, which also faded by week 61 (eFigures 8 and 9 in Supplement 2). Although the coefficient estimates for the influence of the action intervention on automatic process variables aligned with expectations, they were insignificant (Figure 4). Notably, the motivation intervention did not significantly boost motivational process variables relative to the control group.

### Process Evaluation of Program Fidelity and Acceptability

In terms of program fidelity, the proportion of participants satisfying module compliance ranged from two-thirds for the Get Informed (294 of 443 [66%]), Get Activated (88 of 133 [66%]), and Get Energized (84 of 133 [63%]) modules to nearly three-quarters for the Get Motivated module (218 of 284 [77%]) (eTable 8 in Supplement 2). In most weeks, the majority of participants spent less than 5 minutes on each module (eFigures 10 and 11 in Supplement 2); this was less time than expected from piloting. We ran sensitivity analyses excluding the noncompliers and those who spent less time on modules but still found no MVPA treatment effects. With regard to program acceptability, mean ratings for the wearable fitness tracker and each module were consistently above the scale midpoint (3) for all criteria (eFigure 12 in Supplement 2).

## Discussion

Relative to a high baseline level of MVPA, women with prior HPD participating in this trial showed modest within-individual reductions in MVPA. The action intervention positively influenced motivational and volitional processes, and the app and intervention components were rated positively by all groups. However, neither the action intervention nor the motivation intervention spurred a significant difference in MVPA relative to the control. Several factors contribute to the implications of these findings.

### Engagement and Attrition

Intervention engagement was relatively high, and participants positively evaluated the content. Many participants spent less time completing the weekly intervention modules than expected, potentially pointing toward superficial engagement. However, excluding participants with little time spent on modules did not meaningfully alter the results. The study may have been slightly underpowered due to higher-than-expected attrition (final sample size of 443 vs protocol estimate of 504). However, negative treatment effect point estimates at week 9 suggested that a larger sample also would not have yielded treatment effects.

### Benchmarking Against Usual Care

All participants received a wearable fitness tracker and the basic purpose-built physical activity intervention app with features including gamification, which may already have had an influence on participants compared with usual care.^9^ For instance, these may have facilitated self-monitoring, a BCT that targets volitional processes.^67^ This hypothesis is supported by the high level of MVPA at baseline and the suggestive evidence that secondary health outcomes improved for all 3 groups during the trial. This may have created a ceiling effect, making it challenging for the motivation and action interventions to provide additional benefits. Nevertheless, if MVPA reached its peak at baseline, there could have been potential for treatment effects from the motivation and action interventions when MVPA was below that peak in later weeks.

### Automatic Processes

The action intervention significantly influenced motivational and volitional processes relative to the control intervention, but this did not translate into a significant effect on MVPA: our intervention could not bridge the intention-behavior gap. Interestingly, the action intervention did not significantly influence automatic processes. Hence, it remains an open question whether the positive effect on motivational and volitional processes would have translated into higher MVPA if accompanied by a positive impact on automatic processes. This is an important avenue for future research, because previous observational studies suggest that these processes are crucial for bridging the intention-behavior gap.^24,26^

### Highly Active Participants

At baseline, 298 of 435 participants (66%) exceeded the WHO guideline of 150 minutes of MVPA per week; in contrast, only 54% to 59% of the Dutch female population in this age group self-report doing so.^68^ The true proportion of women in the general population reaching these levels is likely even lower, because self-reported MVPA tends to be substantially higher than activity tracker–measured MVPA.^69^ Highly active participants may have found the suggestion to increase their MVPA unnecessary, and referencing the WHO guideline in module content may have unintentionally discouraged them. Our subgroup analysis supports this hypothesis, in that the action intervention worked better for participants with low baseline MVPA. Some studies restrict recruitment to individuals with low levels of MVPA.^70,71^ We did not do so because there is evidence that, even for individuals with high levels of MVPA, increasing MVPA can reduce CVD risk.^72^

### Strengths and Limitations

This study has several strengths. It adds to the evidence base on MVPA interventions by (1) conducting a relatively large RCT with women at increased risk of CVD due to prior HPD, (2) facilitating patient involvement in the intervention design, (3) using 2 treatment groups based on the IBC model and delivering the interventions through an app, (4) linking theoretical constructs to evidence-based BCTs from behavioral sciences, and (5) measuring MVPA with a wearable fitness tracker. To date, only 3 other RCTs have attempted to reduce CVD risk in women with prior HPD, none of which linked theoretical constructs to evidence-based BCTs.^73,74^ App-based interventions offer numerous advantages over face-to-face ones, including cost-effectiveness, wider reach, flexibility, and scalability.^75^ Moreover, a particular strength of this study is that the action group combined the psychological techniques of action and coping planning with commitment as inspired by behavioral economics.

This study also has limitations. One limitation is that the BCTs were not directly connected to increasing MVPA. That is, the modules were successful in boosting motivation and the planning skills required to engage in MVPA but did not provide direct encouragement or concrete exercises to boost the number of minutes spent engaging in MVPA. Future studies should consider creating a tighter link between the app activities and the final goal of the intervention. Other empirical limitations include the following: the relatively large, albeit not unusual, attrition rates; the lack of an objective measure of participant baseline MVPA before receiving the wearable fitness tracker and app; the risk of bias in available case analysis (although we found no evidence of selective attrition, and our findings are robust to per-protocol and imputation-based sensitivity analyses); and the failure of the intervention to meaningfully change automatic processes, which are plausibly important in driving MVPA.

## Conclusions

In this RCT of 2 app-based MVPA interventions (action and motivation) among women with prior HPD, no treatment effects on MVPA were observed. The action intervention positively influenced motivational and volitional psychological processes. The app and intervention components were rated positively by participants, and intervention compliance rates were reasonable. The action intervention exhibited a greater effect on MVPA for those with low baseline MVPA. Potential reasons for the lack of treatment effects were the presence of highly active participants before the trial, a failure to influence automatic processes, a possible disconnect between intervention activities and final outcomes, and unanticipated effects of the control group. These are important considerations for those designing future MVPA interventions and RCTs.

## Notes

Supplement 1.
Trial Protocol

Supplement 2. **eFigure 1.** Study Timeline **eFigure 2.** The Integrated Behavior Change (IBC) Model and the Intention-Behavior Gap **eFigure 3.** Acyclic Behavior Change Diagram **eFigure 4.** Weekly Moderate to Vigorous Physical Activity (MVPA) by Treatment Group for Week 21 Sample **eFigure 5.** Weekly Moderate to Vigorous Physical Activity (MVPA) by Treatment Group for Week 61 Sample **eFigure 6.** Treatment Effects on Moderate to Vigorous Physical Activity (MVPA) Relative to Control: Baseline Week MVPA Interaction Analysis **eFigure 7.** Effects on Moderate to Vigorous Physical Activity (MVPA) Relative to Control: Baseline Week MVPA Interaction Analysis (Intention-to-Treat Analysis) **eFigure 8.** Treatment Effects on Psychological Process Variables Relative to the Control at Week 21 **eFigure 9.** Treatment Effects on Psychological Process Variables Relative to the Control at Week 61 **eFigure 10.** Box Plots of Time Spent on Each Module in Each Intervention Week **eFigure 11.** Box Plots of Time Spent on All Modules in Each Intervention Week **eFigure 12.** Satisfaction Survey Results **eTable 1.** Schematic Overview of Data Collection During the Trial **eTable 2.** Week 9 Between-Group Attrition Check: Test of Differences in Mean Baseline Characteristics of Nonattritors at Week 9 Between Groups **eTable 3.** Week 21 Between-Group Attrition Check: Test of Differences in Mean Baseline Characteristics of Nonattritors at Week 21 Between Groups **eTable 4.** Week 61 Between-Group Attrition Check: Test of Differences in Mean Baseline Characteristics of Nonattritors at Week 61 Between Groups **eTable 5.** Within-Group Attrition Check: Test of Differences Between Mean Baseline Characteristics of Baseline Sample (Includes Attritors) and Week 9 Sample (Excludes Attritors) by Treatment Group **eTable 6.** Within-Group Attrition Check: Test of Differences Between Mean Baseline Characteristics of Baseline Sample (Includes Attritors) and Week 21 Sample (Excludes Attritors) by Treatment Group **eTable 7.** Within-Group Attrition Check: Test of Differences Between Mean Baseline Characteristics of Baseline Sample (Includes Attritors) and Week 61 Sample (Excludes Attritors) by Treatment Group **eTable 8.** Module Completion Compliers by Module (Sample of Nonattritors) **eMethods**. Appropriateness of OLS Regression Model **eFigure 13.** Plots of Observed vs Fitted Values for Moderate to Vigorous Physical Activity (MVPA) Regressions **eFigure 14.** Distribution of Residuals in Moderate to Vigorous Physical Activity (MVPA) Regressions **eAppendix.** i2be App Overview and Module Scripts
eReferences

Supplement 3.
Data Sharing Statement
