# Supplementary material for: App-Based Physical Activity Intervention Among Women With Prior Hypertensive Pregnancy Disorder: A Randomized Clinical Trial
Source: JAMA Netw Open. 2025 Apr 2;8(4):e252656. doi: 10.1001/jamanetworkopen.2025.2656 (PMC11966332; doi:10.1001/jamanetworkopen.2025.2656)
Supplement: Supplement 1. — Trial Protocol [file jamanetwopen-e252656-s001.pdf]

Protocol Title: **From intention to behavior with i2be:**

a randomized controlled trial protocol for an app-based physical activity intervention

Short Title: i2be

Version: 2

Date: 29-03-2021

Project leader: Anne I. Wijtzes, PhD (a.wijtzes@erasmusmc.nl)

Sponsor: This study was commissioned by the Erasmus Medical Center and the Erasmus University Rotterdam through the Smarter Choices for Better Health Initiative (Action Line Prevention).

Subsidizing party: Funding for the study was by the Smarter Choices for Better Health Initiative (Action Line Prevention) and the Erasmus Trustfonds.

Summary

Rationale: Physical activity is an important determinant of cardiovascular health. Therefore, health behavior interventions targeting physical activity are highly warranted. Most interventions have limited and short-term effects, likely because they fail to bridge the intention-behavior gap.

Objective: The current study will test a dual system theory- and evidence-based e-health intervention, and additionally, provide important insight into the contribution of volitional and automatic processes in bridging the intention-behavior gap and achieving sufficient and sustained physical activity.

Study design: The effectiveness of the intervention will be tested using a three-arm randomized controlled trial implemented through an app.

Study population: Women with a prior hypertensive disorder of pregnancy

Intervention: Following i2be app registration and stratification on baseline factors, participants will be randomly allocated (1:1:1) in-app to the control group, treatment 1, or treatment 2. The control group receives the module ‘Get Informed’ consisting of usual care, i.e. knowledge provision. In addition to the module ‘Get Informed’, treatment 1 receives the module ‘Get Motivated’ (targeting motivational processes), consisting of the behavior change technique (BCT) motivational interviewing-based counselling. In addition to the modules ‘Get Informed’ and ‘Get Motivated’, treatment 2 receives the module ‘Get Activated’ (targeting volitional processes), consisting of the BCTs action planning training and reminders, coping planning training, and commitment training, and the module ‘Get Energized’ (targeting automatic processes), consisting of the BCTs mindfulness-based stress reduction and positive psychology.

Main study parameters/endpoints: The primary outcome is objectively measured weekly minutes of moderate-to-vigorous physical activity. Secondary outcomes include

objectively measured (resting heart rate) and self-reported physiology measurements (body mass index, waist-hip-ratio, cardiorespiratory fitness), as well as subjective well-being. Tertiary outcomes include self-reported mechanism of action variables in order to assess the mechanisms underlying the effects of BCTs. Objectively measured outcomes will be captured by Fitbit activity trackers (Fitbit Inspire 2), and all other outcomes measures will be self-reported into the i2be app. Outcome measures will be assessed at baseline, immediately post-intervention, at 3 months follow-up, and at 12 months follow-up. Physical activity will additionally be assessed at the intervention midpoint. Effectiveness will be determined by a modified intention-to-treat analysis. Additionally, a process evaluation will be performed immediately post-intervention.

Nature and extent of the burden and risks associated with participation, benefit and group relatedness: Not applicable.

1. Introduction and rationale

The reduction of cardiovascular disease (CVD) is a public health priority, as CVDs are among the leading causes of poor health worldwide [1]. Insufficient physical activity, poor dietary habits, tobacco, and alcohol use are some of the most important behavioral risk factors for CVD [1]. Cardio metabolic risk factors for CVD such as hypertension, overweight and obesity often go hand in hand with behavioral risk factors for CVD [2, 3]. Some cardio metabolic risk factors for CVD are specific to women, including hypertensive pregnancy disorders [4, 5]. For example, preeclampsia is a hypertensive pregnancy disorder complicating two to eight percent of pregnancies worldwide, leading to acute clinical consequences of maternal and neonatal morbidity and mortality, and a two to seven fold risk for CVD later in life in comparison to women with a normotensive pregnancy [6, 7].

A substantial proportion of CVD morbidity and mortality can be prevented by individuals leading healthy lifestyles [5, 8]. International guidelines advise adults to accumulate at least 150 minutes of moderate physical activity, 75 minutes of vigorous physical activity, or an equivalent combination of moderate- and vigorous-intensity physical activity (MVPA) per week [9]. Most people, and especially women, fail to meet these guidelines [1]. Therefore, the implementation of health behavior change interventions targeting MVPA are highly warranted [3]. Such interventions are likely to provide an especially high social return in women with hypertensive disorders of pregnancy, primarily due to the importance of optimizing the cardiovascular health of this population [5, 10-12].

Health behavior change interventions targeting MVPA are widely applied in the general population, rarely achieving large and long-term effects [13]. A potential explanation for the limited effects of these interventions are their lack of theoretical basis, and no systematic linking of evidence-based behavior change techniques (BCTs) to specific mechanism of action (MOA) variables [14]. Another explanation is their use of traditional socio-cognitive theories (e.g., self-determination theory, theory of planned behavior), which describe behavior as solely the result of deliberative (also called rational or conscious) psychological processes, with underlying MOA variables such as knowledge,

attitudes, or intention [15]. Indeed, while well-established deliberative theories reliably succeed at predicting behavioral intention, they regularly fail to predict actual behavior, leading to the infamous 'intention-behavior gap' [16]. A potential explanation for this shortcoming is their underestimation of automatic (also called impulsive or non-conscious) processes involved in health behavior, with underlying MOA variables such as affect, stress, and habit [15, 17].

The notion that behavior, at least in part, is influenced by factors beyond individual awareness is not new – the school of thought of behaviorism is a prominent example [18]. However, interest in the integration of both types of processes into one theory is relatively new, partly brought about by deliberative theories' inability to provide a full account of human behavior, and partly by developments in the understanding of automatic processes [15]. Dual-system theories account for both types of processes, i.e. automatic processes generating impulses, and deliberative processes controlling these impulses [15, 19-21]. The present intervention is based on a specific dual-system theory, i.e. the integrated behavior change (IBC) model [22].

The IBC model integrates insights of well-established health behavior theories to explain the workings of deliberative processes and automatic processes involved in physical activity [22-24]. The IBC model separates deliberative processes into two sub-types, i.e. motivational and volitional processes, thereby differentiating between three main types of processes in total [22]. This clear distinction between pre-intentional (motivational) and post-intentional (volitional) processes, as well as accounting for automatic processes, may be key in bridging the 'intention-behavior gap' [16]. Since the development of the first IBC model, several observational studies have updated the core model and used it to explain a plethora of health behaviors, from fat and sugar intake to sunscreen use [25-33].

The current intervention is among the very first interventions to use the IBC model as its theoretical framework [34]. Furthermore, we have systematically selected BCTs linking to the MOA variables described by the IBC model [14, 35]. The selection of BCTs was

guided by systematic literature reviews, meta-analyses and evidence syntheses examining the association between BCTs and theory [36] and theoretical domains [37], intervention effect evaluations [38, 39], intervention process evaluations [40], and previous research showing the effectiveness of these BCTs [41-49].

When it comes to the delivery of interventions, e-health interventions have several advantages over face-to-face interventions, i.e. they are comparatively low cost, have a wide reach, and provide flexibility in intervention location and time [50, 51]. Previous e-health interventions that used a tailored design, i.e. provided participants with primary task support, dialogue support and social support, showed superior effects in fostering user engagement [52, 53] and in increasing MVPA [51, 54] over interventions that took a non-tailored approach. Due to the considerations above we chose to deliver the current intervention in e-health format, more specifically, as a tailored mobile phone application (app) providing primary task support and dialogue support that can be downloaded to any smartphone.

The aim of the study is to determine the effectiveness of a dual system theory- and evidence-based e-health intervention using a three-arm randomized controlled trial (RCT). Furthermore, our study design will allow us to gain important scientific knowledge regarding the theoretical framework on which the intervention was based (i.e. the IBC model) by providing insight into the contribution of motivational, volitional, and automatic processes in leaping across the intention-behavior gap and achieving sufficient and sustained physical activity.

2. Objective

Primary objective: The objective of the study is to determine the effectiveness of a dual system theory- and evidence-based e-health intervention to sustainably increase physical activity using a three-arm randomized controlled trial (RCT).

Secondary objective: Our study design will allow us to gain important scientific knowledge regarding the theoretical framework on which the intervention was based (i.e. the IBC

model) by providing insight into the contribution of motivational, volitional, and automatic processes in leaping across the intention-behavior gap and achieving sufficient and sustained physical activity.

3. Study design

The randomized controlled trial (RCT) will have a duration of eight weeks. All participant interactions (e.g. sign up, intervention, data collection, and participation incentives) occur via the purpose-built i2be app. The user-interface of i2be shows a diverse range of women of ethnic backgrounds and ages (20-50 years) representative of the population, and uses female voice-overs in the instruction video and audio clips. The modules ‘Get Informed’, ‘Get Motivated’, ‘Get Activated’ and ‘Get Energized’ consist of interactive, fully automated activities each week, i.e. participants are prompted to reflect and engage with the activities, without interaction with the researcher.

4. Study population

4.1. Population base

The population base will consist of women who have experienced a hypertensive disorder of pregnancy [4-6]. Given that the associated CVD risk is largest for women with a history of preeclampsia, especially severe preeclampsia [4, 6], we first draw participants from this population. If necessary, we will further include women with other hypertensive disorders of pregnancy in a stepwise manner (see Recruitment).

4.2. Inclusion criteria

In order to be eligible for participation in the trial, a participant must meet the following criteria:

- Experienced a hypertensive disorder of pregnancy (e.g. preeclampsia, eclampsia, HELLP syndrome) in the past.

4.3. Exclusion criteria

A person who meets any of the following criteria will not be eligible for participation in this study:

- <18 years of age
- Pregnant at time of inclusion
- <3 months post-partum
- Any physical health limitations preventing physical activity (e.g. illness, injury, surgery, rehabilitation)
- No working knowledge of Dutch or English language
- No possession of a smartphone

4.4. Sample size calculation

A meta-analyses of recent smartphone-based physical activity interventions found a small to medium average effect size (standardized mean difference) of 0.31 on daily minutes of physical activity [55]. The effect sizes in individual studies ranged between -0.29 to 0.70. We expect the main treatment effect in our study (T2 v C) to be towards the higher end of this range. Our second treatment effect (T2 versus T1) is expected to be somewhat smaller. Given this, we aim to recruit 600 participants to the trial, randomized in a 1:1:1 allocation ratio to the three arms (200:200:200 participants). The average attrition rate in previous web-based physical activity interventions was 20% [56]. However, as we incentivize participation throughout the duration of our study using both psychological and tangible rewards, we estimate that attrition will be 15% in our study. This would leave us with a final total sample size of 510, once attrition is accounted for. Taking into account the potential maximum deviation from a 1:1:1 allocation ratio which may arise due to the stratified block randomization procedure, our intended sample size (n=600) will give us power to detect effect sizes greater than or equal to the average effect found in the meta-analysis (0.31), which should be adequate to detect our main treatment effect of T2 versus C.

4.5. Recruitment

In the Netherlands, unique cardiovascular follow-up and care is provided to women with prior severe preeclampsia at the multidisciplinary Follow-Up Pre-Eclampsia Outpatient Clinic (FUPEC) of the Erasmus MC, the only clinic of its kind in the country [57]. There are currently around 1000 women involved in the clinic with an additional 100 to 150

women enrolling each year. An email to participate in the RCT will be sent to all FUPEC participants by their health care professionals, containing a short description of the study, an introduction video to the i2be app, and a link to an eligibility questionnaire (for more detail, see section on Implementation). In case the intended sample size (n=600) cannot be fully recruited from the FUPEC clinic, further recruitment will take place through the national patient organization for women with a history of preeclampsia (Hellp Stichting). Subsequent venues for recruitment include recruitment through the Department of Gynecology and Obstetrics (Erasmus MC), first among women with prior (non-severe) preeclampsia, and second among women with other hypertensive disorders of pregnancy [4], as well as similar departments of regional hospitals.

Invited women are informed that participation in the trial is voluntary, and that declining to participate does not affect their care from the Erasmus MC (or other hospitals). Women who choose to participate will be asked to sign an informed consent for trial participation and use of data for research purposes. Participants are informed that they may leave the trial at any point in time without having to provide a reason and without consequences for their care.

5. Treatment of subjects

All participants receive the components specified under standard treatment. Only participants in treatment 1 and treatment 2 receive the relevant investigational treatments. Participants are not prescribed, but are solely advised physical activity and all other activities, and participants are free to deviate from this advice.

5.1. Standard treatment

5.1.1. Get Informed

All participants use the i2be app and their Fitbit MVPA tracker. Participants will need to sync their Fitbit with the i2be app on a weekly basis for optimal data collection. All participants receive the module 'Get Informed' consisting of usual care. More specifically, information is provided on a) the international guidelines, examples, and health effects of physical activity, b) the relationship between hypertensive disorders of pregnancy,

increased risk of cardiovascular events, and physical activity, and c) support available for women with a prior hypertensive disorder of pregnancy.

5.1.2. Incentives

For all participants, the completion of modules and outcome measures and syncing of the Fitbit (i.e. trial participation) are incentivized with a virtual point system (i2be points) resulting in psychological rewards, i.e. progress though virtual levels of achievement and the receipt of digital trophies through the app. The virtual i2be points are further used to qualify for tangible rewards. For every week of the intervention and follow up measurement points, participants qualify to enter into a weekly raffle for a self-selected physical activity-related voucher (i.e. Decathlon.nl, Sportenfitcadeu.nl, Fysiosupplies.nl), under the condition that they earn the maximum i2be points for that week. The value of these vouchers increases from 25€ during the intervention to 30€ for the two final follow-up measurement points (i.e. 3- and 12-months post intervention) to encourage participants to engage in long-term follow up. Lastly, conditional on reaching a threshold of accumulated i2be point, participants qualify to keep the Fitbit at the end of the trial.

5.1.3. Objective measures

The primary outcome of this research project is objectively measured MVPA, as measured by the Fitbit. One of the secondary outcomes is objectively measured resting heart rate, also measured by the Fitbit.

5.1.4. Home measurements manually entered into app

See appendix F1 for home measurements manually entered into app.

5.1.5. Questionnaires filled in within app

See appendix F1 for questionnaires.

5.2. Investigational treatment

5.2.1. Treatment 1

In addition to receiving standard treatment, treatment 1 receives the module 'Get Motivated' (targeting motivational processes), consisting of the behavior change technique (BCT) motivational interviewing-based counselling. Participants receive an interactive, fully automated activity each week, consisting of content-based motivational interviewing techniques, namely: Running Head Start, Importance Ruler, Confidence Ruler, Looking Forward, Hypothetical Thinking, Query Extremes, Identifying Past Successes, Identifying Strengths, Values Exploration, Normalizing, and Goal Attainment Scaling [58].

5.2.2. Treatment 2

In addition to receiving standard treatment and the module 'Get Motivated', treatment 2 receives the module 'Get Activated' (targeting volitional processes), consisting of the BCTs action planning training and reminders, coping planning training, and commitment training. Participants receive interactive, fully automated activities each week: drop-down menus aid them in making action plans and coping plans [45]; they are reminded of their action plans by a reminder; they are asked to make a weekly MVPA goal, and offered the option to commit to that goal with their i2be points; and they are taught how to make their own commitments outside of i2be [59].

Furthermore, treatment 2 receives the module 'Get Energized' (targeting automatic processes), consisting of the BCTs mindfulness-based stress reduction and positive psychology. Participants receive four-minute audio clips of mindfulness-based stress reduction, namely Introduction to Mindfulness, Loving Kindness, Breath Awareness, Body Scan, Validation, Sensations, Gratitude, and Humor Therapy [49, 60]; and interactive, fully automated positive psychology exercises, namely Three Good Things, Kindness, Three Beautiful Things, Gratitude, Three Amusing Things, Savoring, Validation, and Power Posing [46, 47].

6. General considerations

6.1. Handling of dropouts

Participants can leave the trial at any time following consent without any consequences. Participants dropping out of the study will not be substituted. Following drop-out no further data will be collected.

7. Outcomes

7.1. Primary outcome

Outcome measures are collected at baseline, immediately post-intervention, 3 months follow-up, and 12 months follow-up (see appendix F1 for schematic overview of data collection during the trial). Physical activity will additionally be assessed at the intervention midpoint. The primary outcome of this research project is objectively measured MVPA, as measured by the Fitbit.

7.2. Secondary outcomes

Secondary outcomes consist of physiological and well-being measures. Resting heart rate will be measured by the Fitbit. Other physiological measures are taken at home and self-reported in i2be by participants, including body mass index, waist-to-hip ratio, and cardiorespiratory fitness. Text instructions on the correct performance of home measurements will be provided to participants in-app. A soft measuring tape will be sent to participants along with the Fitbit. Subjective well-being is self-reported in-app.

7.3. Tertiary outcomes

Tertiary outcomes include self-reported MOA variables in order to assess the mechanisms underlying the effects of BCTs.

7.4. Confounders

To adjust data-analyses for potential confounders, information on type of blood pressure condition, age, lactation status, household composition, education, trait self-control, and habit is self-reported in-app at baseline.

8. Interim analysis and stopping rules

We anticipate no safety issues with this study. As such, no interim analysis is planned, and no stopping rules are defined.

9. Randomization

9.1. Stratification and randomization

Stratification and randomization will be carried out automatically by the app.

9.2. Allocation concealment mechanism

Inherent to the intervention and study design, participants will not be blinded to allocation. The research team will be blinded to the allocation of participants at the randomization stage and throughout the study.

9.3. Implementation

Following email invitation from health care professionals (in case of hospital recruitment) or the research team (in case of recruitment through patient organizations), interested participants click on a link in the email that will lead them to an eligibility questionnaire hosted on the researcher platform of Avegen (digital health company responsible for developing and hosting the researcher platform and participant app). Once eligibility has been established, the participant will be asked for informed consent. Following informed consent, participants will be asked their name, email address, and postal address to send the Fitbit and a soft measuring tape. Next, participants will receive an email from Avegen to download the i2be and Fitbit apps from the Google Play Store (for Android phones) or App Store (for iPhones), a pdf of the Welcome Pack, and a message that the Fitbit will be sent in the post mail. The Welcome Pack consists of instructions on the configuration of the Fitbit app and the i2be app, an explanation of the Fitbit device, and an explanation on the i2be points and reward system.

When using the i2be app for the first time, participants will be asked to choose their preferred language (Dutch or English) and register with their email address. Subsequently, participants will be stratified on two factors (i.e. on time since pregnancy [< 12 months post-partum versus ≥ 12 months post-partum] and self-reported baseline MVPA [low, medium, high]), and randomly allocated (1:1:1) in-app to one of the three intervention arms, i.e. the control group (C), treatment 1 (T1), or treatment 2 (T2). The

participants will be shown a 2 minute video that introduces i2be. The 8 week intervention period will then start.

10. Statistical analysis

10.1. Primary analysis

The primary analysis will be a modified intention-to-treat analysis, the deviation from a full intention-to-treat analysis being that we exclude participants with missing dependent variable data (available case analysis). Linear regression will be used to assess differences between groups. The main test of i2be is the test T2 versus C in order to gain insight into the effectiveness of targeting the three types of processes hypothesized to impact MVPA. Second, we aim to test differences between T2 and T1 to gain insight into the contribution of volitional and automatic processes over motivational processes only in bridging the intention behavior gap. Third, we will test the difference between T1 and C to gain insight into the differential effectiveness of targeting motivational processes compared to treatment as usual. Variation in MOA variables will be investigated to gain insight into the mechanisms underlying the effects of BCTs. Sensitivity analyses will be carried out to assess the robustness of results to the missing data strategy adopted.

10.2. Subgroup analyses

To assess whether the e-health intervention has differential effects across educational level, subgroup analysis by educational level will be performed.

10.3. Additional analyses

A test of the IBC model will be conducted using structural equation modelling on the relationships between MOA variables as hypothesized by the model. Process evaluation of the intervention will entail fidelity measures (i.e. measures of participants' interaction with the app), as well as qualitative research among participants on their experiences with and feelings regarding the app.

11. Safety considerations

Given the nature of the intervention participant safety issues are not anticipated in this study. As such no recording of adverse events will be undertaken as part of the study.

12. Ethical considerations

Staff at the research site will adhere most strictly to all applicable legal, ethical, and safety provisions of the Netherlands and the EU. The study will be conducted in accordance with the principles of the Declaration of Helsinki [61]. No minors and/or incapacitated adults will participate in this study. To obtain dispensation from the statuary obligation to provide insurance, because participating in the study is without risks, we will submit a reasoned request to the accredited METC.

13. Administrative aspects, monitoring and publication

13.1. Handling and storage of data and documents

Erasmus MC (Department of Public Health) is owner ("eigenaar") of all data generated in this study. Digital health company Avegen will act as a trusted third party, i.e. they are responsible for data capture and encryption of data. During the duration of the study, Avegen will safely capture and separately store personal data and research data. Their data capture systems (i.e. platform for researchers and app for participants) comply with strict international guidelines on data safety and privacy and undergo frequent pen testing.

Pseudonomized research data, containing only a unique participant ID (not based on participant data), will be transferred into the safe Research Environment of the Erasmus MC in three data dumps, i.e. post intervention, after 3 months follow-up, and after 12 months follow-up. The Research Environment is a safe work place by being able to invite researchers to work in the workspace, allocate different roles to these researchers, and using a 2-factor authentication log in procedure. Following the final data dump of research data, Avegen will delete all research data on their end, making Erasmus MC the sole owner (i.e. "eigenaar en bezitter") of the research data.

During the study, the identification key linking unique participant ID with personal data will be held by Avegen. Only in the case of necessary participant communication (e.g. sending out Fitbits), i2be researchers will receive the necessary personal information, not including the unique participant ID. Following the final research data dump (i.e. end of project) Avegen will safely transfer the informed consent forms and the identification key to the Erasmus MC, after which they delete all personal data on their end, making Erasmus MC the sole owner (i.e. "eigenaar en bezitter") of personal data (i.e. informed consent forms). These personal data will be saved in an ID-project folder on the secure V-drive of one of the i2be researchers (Erasmus MC).

Pseudonomized research data and personal data will be archived in separate folders for a minimum of 10 years after completion of the study (X-drive). In light of open access research, pseudonomized research data collected during this study will be made available to internal and external researchers upon request, only for those participants who have proved informed consent to do so.

The research team works closely with the IT specialists/ Information Security Officers of the Erasmus MC and Avegen to ensure that all data are handled confidentially and with upmost care. So far, a classification report of the app has been drafted and the data processing agreement has been approved. Pending on the final technical requirements of the researcher platform and participant app, a document on security requirements and measures will be drafted and shared with the Information Security Officers.

13.2. Monitoring and quality assurance

Not applicable.

13.3. Amendments

Amendments are changes made by the project leader after a favorable opinion by the accredited ethics committee has been given.

13.4. End of the study report

The project leader will notify the accredited METC of the end of the study within a period of 8 weeks after the last participant's last measurement. In the case the study is ended prematurely, the project leader will notify the accredited METC within 15 days, including the reasons for premature termination. Within one year after the end of the study, the project leader will submit a final study report with the results of the study, including any publications/ abstracts of the study, to the accredited METC.

13.5. Public disclosure and publication policy

The trial will be registered with the Netherlands Trial Register prior to start of inclusion. Findings from the study will be presented at national and international scientific conferences. Furthermore, articles reporting on these findings will be submitted for publication in leading international peer-reviewed journals. Results will be communicated to the general public through general conferences, meetings, and with publication in the newsletter(s) of relevant patient organizations.

References

1. Salim, S.V., et al., *Heart Disease and Stroke Statistics—2020 Update: A Report From the American Heart Association.* Circulation, 2020. **141**(9): p. e139-e596.
2. Cannon, C.P., *Cardiovascular disease and modifiable cardiometabolic risk factors.* Clinical cornerstone, 2007. **8**(3): p. 11-28.
3. Thomas, H., et al., *Global Atlas of Cardiovascular Disease.* Global heart, 2018. **13**(3).
4. Heida, K.Y., et al., *Cardiovascular risk management after reproductive and pregnancy-related disorders: a Dutch multidisciplinary evidence-based guideline.* European journal of preventive cardiology, 2016. **23**(17): p. 1863-1879.
5. Mosca, L., et al., *Effectiveness-based guidelines for the prevention of cardiovascular disease in women—2011 update: a guideline from the American Heart Association.* Journal of the American College of Cardiology, 2011. **57**(12): p. 1404-1423.
6. Bellamy, L., et al., *Pre-eclampsia and risk of cardiovascular disease and cancer in later life: systematic review and meta-analysis.* Bmj, 2007. **335**(7627): p. 974.
7. Ahmed, R., et al., *Pre-eclampsia and future cardiovascular risk among women: a review.* Journal of the American College of Cardiology, 2014. **63**(18): p. 1815-1822.
8. Authors/Task Force, M., et al., *European Guidelines on cardiovascular disease prevention in clinical practice (version 2012) The Fifth Joint Task Force of the European Society of Cardiology and Other Societies on Cardiovascular Disease Prevention in Clinical Practice (constituted by representatives of nine societies and by invited experts) Developed with the special contribution of the European Association for Cardiovascular Prevention & Rehabilitation (EACPR).* European heart journal, 2012. **33**(13): p. 1635-1701.
9. World Health, O., *Information sheet: Global recommendations on physical activity for health 18–64 years old. 2011*. 2011.
10. Magee, L.A. and P. Von Dadelszen, *Pre-eclampsia and increased cardiovascular risk*. 2007, British Medical Journal Publishing Group.
11. Newstead, J., P. Von Dadelszen, and L.A. Magee, *Preeclampsia and future cardiovascular risk.* Expert review of cardiovascular therapy, 2007. **5**(2): p. 283-294.
12. Sattar, N. and I.A. Greer, *Pregnancy complications and maternal cardiovascular risk: opportunities for intervention and screening?* Bmj, 2002. **325**(7356): p. 157-160.
13. Kahn, E.B., et al., *The effectiveness of interventions to increase physical activity: a systematic review.* American journal of preventive medicine, 2002. **22**(4): p. 73-107.
14. Michie, S., et al., *From theory-inspired to theory-based interventions: A protocol for developing and testing a methodology for linking behaviour change techniques to theoretical mechanisms of action.* Annals of Behavioral Medicine, 2017. **52**(6): p. 501-512.
15. Hagger, M.S., *Non-conscious processes and dual-process theories in health psychology*. 2016, Taylor & Francis.

16. Sniehotta, F.F., U. Scholz, and R. Schwarzer, *Bridging the intention–behaviour gap: Planning, self-efficacy, and action control in the adoption and maintenance of physical exercise.* Psychology & Health, 2005. **20**(2): p. 143-160.
17. Kahneman, D., *Thinking, fast and slow*. 2011: Macmillan.
18. Bargh, J.A. and M.J. Ferguson, *Beyond behaviorism: on the automaticity of higher mental processes.* Psychological bulletin, 2000. **126**(6): p. 925.
19. Loewenstein, G., *Out of control: Visceral influences on behavior.* Organizational behavior and human decision processes, 1996. **65**(3): p. 272-292.
20. De Ridder, D.T.D., et al., *Taking stock of self-control: A meta-analysis of how trait self-control relates to a wide range of behaviors.* Personality and Social Psychology Review, 2012. **16**(1): p. 76-99.
21. Hofmann, W., M. Friese, and R.W. Wiers, *Impulsive versus reflective influences on health behavior: A theoretical framework and empirical review.* Health Psychology Review, 2008. **2**(2): p. 111-137.
22. Hagger, M.S. and N.L.D. Chatzisarantis, *An integrated behavior change model for physical activity.* Exercise and Sport Sciences Reviews, 2014. **42**(2): p. 62-69.
23. Ajzen, I., *The theory of planned behavior.* Organizational behavior and human decision processes, 1991. **50**(2): p. 179-211.
24. Deci, E.L.R., R. M. , *Self-determination theory: when mind mediates behavior.* Journal of Mind and Behavior 1980. **1**(1): p. 33-43.
25. Hagger, M.S., et al., *Predicting sugar consumption: Application of an integrated dual-process, dual-phase model.* Appetite, 2017. **116**: p. 147-156.
26. Hamilton, K., et al., *Child sun safety: Application of an Integrated Behavior Change model.* Health Psychology, 2017. **36**(9): p. 916.
27. Brown, D.J., et al., *Predicting fruit and vegetable consumption in long-haul heavy goods vehicle drivers: Application of a multi-theory, dual-phase model and the contribution of past behaviour.* Appetite, 2018. **121**: p. 326-336.
28. Caudwell, K.M., et al., *Reducing alcohol consumption during pre-drinking sessions: Testing an integrated behaviour-change model.* 2018.
29. Galli, F., et al., *Active lifestyles in older adults: an integrated predictive model of physical activity and exercise.* Oncotarget, 2018. **9**(39): p. 25402-25413.
30. Hamilton, K., et al., *Being active in pregnancy: Theory-based predictors of physical activity among pregnant women.* Women & Health, 2018.
31. Shannon, S., et al., *Predicting student-athlete and non-athletes' intentions to self-manage mental health: Testing an integrated behaviour change model.* Mental Health & Prevention, 2019. **13**: p. 92-99.
32. Hamilton, K., et al., *Reasoned and implicit processes in heavy episodic drinking: An integrated dual‐process model.* British journal of health psychology, 2020. **25**(1): p. 189-209.
33. Phipps, D.J., M.S. Hagger, and K. Hamilton, *Predicting limiting 'free sugar'consumption using an integrated model of health behavior.* Appetite, 2020: p. 104668.
34. Kwasnicka, D., et al., *Comparing motivational, self-regulatory and habitual processes in a computer-tailored physical activity intervention in hospital employees-protocol for the PATHS randomised controlled trial.* BMC public health, 2017. **17**(1): p. 1-16.

35. Michie, S., et al., *The behavior change technique taxonomy (v1) of 93 hierarchically clustered techniques: building an international consensus for the reporting of behavior change interventions.* Annals of behavioral medicine, 2013. **46**(1): p. 81-95.
36. Prestwich, A., et al., *Does theory influence the effectiveness of health behavior interventions? Meta-analysis.* Health Psychology, 2014. **33**(5): p. 465.
37. French, S.D., et al., *Developing theory-informed behaviour change interventions to implement evidence into practice: a systematic approach using the Theoretical Domains Framework.* Implementation Science, 2012. **7**(1): p. 1-8.
38. Michie, S., et al., *Effective techniques in healthy eating and physical activity interventions: a meta-regression.* Health Psychology, 2009. **28**(6): p. 690.
39. Dombrowski, S.U., et al., *Optimizing acceptability and feasibility of an evidence-based behavioral intervention for obese adults with obesity-related co-morbidities or additional risk factors for co-morbidities: an open-pilot intervention study in secondary care.* Patient education and counseling, 2012. **87**(1): p. 108-119.
40. Araújo-Soares, V., et al., *Development and exploratory cluster-randomised opportunistic trial of a theory-based intervention to enhance physical activity among adolescents.* Psychology and Health, 2009. **24**(7): p. 805-822.
41. Hagger, M.S., N.L.D. Chatzisarantis, and S.J.H. Biddle, *The influence of autonomous and controlling motives on physical activity intentions within the Theory of Planned Behaviour.* British Journal of Health Psychology, 2002. **7**(3): p. 283-297.
42. Martins, R.K. and D.W. McNeil, *Review of motivational interviewing in promoting health behaviors.* Clinical psychology review, 2009. **29**(4): p. 283-293.
43. Friederichs, S.A.H., et al., *Exploring the working mechanisms of a web-based physical activity intervention, based on self-determination theory and motivational interviewing.* Internet interventions, 2016. **3**: p. 8-17.
44. Carraro, N. and P. Gaudreau, *Spontaneous and experimentally induced action planning and coping planning for physical activity: A meta-analysis.* Psychology of Sport and Exercise, 2013. **14**(2): p. 228-248.
45. Sniehotta, F.F., U. Scholz, and R. Schwarzer, *Action plans and coping plans for physical exercise: A longitudinal intervention study in cardiac rehabilitation.* British journal of health psychology, 2006. **11**(1): p. 23-37.
46. Hendriks, T., et al., *The efficacy of multi-component positive psychology interventions: A systematic review and meta-analysis of randomized controlled trials.* Journal of happiness studies, 2020. **21**(1): p. 357-390.
47. Labarthe, D.R., et al., *Positive cardiovascular health: a timely convergence.* Journal of the American College of Cardiology, 2016. **68**(8): p. 860-867.
48. Chiesa, A. and A. Serretti, *Mindfulness-based stress reduction for stress management in healthy people: a review and meta-analysis.* The journal of alternative and complementary medicine, 2009. **15**(5): p. 593-600.
49. Meyer, J.D., et al., *Benefits of 8-wk mindfulness-based stress reduction or aerobic training on seasonal declines in physical activity.* Medicine and science in sports and exercise, 2018. **50**(9): p. 1850.

50. Lustria, M.L.A., et al., *Computer-tailored health interventions delivered over the Web: review and analysis of key components.* Patient education and counseling, 2009. **74**(2): p. 156-173.
51. Neville, L.M., B. O'Hara, and A. Milat, *Computer-tailored physical activity behavior change interventions targeting adults: a systematic review.* International Journal of Behavioral Nutrition and Physical Activity, 2009. **6**(1): p. 30.
52. Kelders, S.M., et al., *Persuasive system design does matter: a systematic review of adherence to web-based interventions.* Journal of medical Internet research, 2012. **14**(6): p. e152.
53. Mohr, D., P. Cuijpers, and K. Lehman, *Supportive accountability: a model for providing human support to enhance adherence to eHealth interventions.* Journal of medical Internet research, 2011. **13**(1): p. e30.
54. Kroeze, W., A. Werkman, and J. Brug, *A systematic review of randomized trials on the effectiveness of computer-tailored education on physical activity and dietary behaviors.* Annals of behavioral medicine, 2006. **31**(3): p. 205-223.
55. Feter, N., et al., *What is the role of smartphones on physical activity promotion? A systematic review and meta-analysis.* International Journal of Public Health, 2019. **64**(5): p. 679-690.
56. Davies, C.A., et al., *Meta-analysis of internet-delivered interventions to increase physical activity levels.* International Journal of Behavioral Nutrition and Physical Activity, 2012. **9**(1): p. 52.
57. Benschop, L., et al., *Blood pressure profile 1 year after severe preeclampsia.* Hypertension, 2018. **71**(3): p. 491-498.
58. Hardcastle, S.J., et al., *Identifying content-based and relational techniques to change behaviour in motivational interviewing.* Health psychology review, 2017. **11**(1): p. 1-16.
59. Gharad, B., K. Dean, and N. Scott, *Commitment Devices.* Annual Review of Economics, 2010. **2**(1): p. 671-698.
60. Grossman, P., et al., *Mindfulness-based stress reduction and health benefits: A meta-analysis.* Journal of psychosomatic research, 2004. **57**(1): p. 35-43.
61. Assembly, W.M.A.G. and O. Seoul, *Declaration of Helsinki.* Proceedings of the 64th WMA General Assembly, Fortaleza, Brazil, 2013: p. 11-14.
