# Supplementary material for: App-Based Physical Activity Intervention Among Women With Prior Hypertensive Pregnancy Disorder: A Randomized Clinical Trial
Source: JAMA Netw Open. 2025 Apr 2;8(4):e252656. doi: 10.1001/jamanetworkopen.2025.2656 (PMC11966332; doi:10.1001/jamanetworkopen.2025.2656)
Supplement: Supplement 2. — eFigure 1. Study Timeline eFigure 2. The Integrated Behavior Change (IBC) Model and the Intention-Behavior Gap eFigure 3. Acyclic Behavior Change Diagram eFigure 4. Weekly Moderate to Vigorous Physical Activity (MVPA) by Treatment Group for Week 21 Sample eFigure 5. Weekly Moderate to Vigorous Physical Activity (MVPA) by Treatment Group for Week 61 Sample eFigure 6. Treatment Effects on Moderate to Vigorous Physical Activity (MVPA) Relative to Control: Baseline Week MVPA Interaction Analysis eFigure 7. Effects on Moderate to Vigorous Physical Activity (MVPA) Relative to Control: Baseline Week MVPA Interaction Analysis (Intention-to-Treat Analysis) eFigure 8. Treatment Effects on Psychological Process Variables Relative to the Control at Week 21 eFigure 9. Treatment Effects on Psychological Process Variables Relative to the Control at Week 61 eFigure 10. Box Plots of Time Spent on Each Module in Each Intervention Week eFigure 11. Box Plots of Time Spent on All Modules in Each Intervention Week eFigure 12. Satisfaction Survey Results eTable 1. Schematic Overview of Data Collection During the Trial eTable 2. Week 9 Between-Group Attrition Check: Test of Differences in Mean Baseline Characteristics of Nonattritors at Week 9 Between Groups eTable 3. Week 21 Between-Group Attrition Check: Test of Differences in Mean Baseline Characteristics of Nonattritors at Week 21 Between Groups eTable 4. Week 61 Between-Group Attrition Check: Test of Differences in Mean Baseline Characteristics of Nonattritors at Week 61 Between Groups eTable 5. Within-Group Attrition Check: Test of Differences Between Mean Baseline Characteristics of Baseline Sample (Includes Attritors) and Week 9 Sample (Excludes Attritors) by Treatment Group eTable 6. Within-Group Attrition Check: Test of Differences Between Mean Baseline Characteristics of Baseline Sample (Includes Attritors) and Week 21 Sample (Excludes Attritors) by Treatment Group eTable 7. Within-Group Attrition Check: Test of Differences Betw [file jamanetwopen-e252656-s002.pdf]

Supplemental Online Content

Kókai LL, Ó Ceallaigh D, Wijtzes AI, et al. App-based physical activity intervention among women with prior hypertensive pregnancy disorder: a randomized clinical trial. *JAMA Netw Open*. 2025;8(4):e252656. doi:10.1001/jamanetworkopen.2025.2656

eFigure 1. Study Timeline

eFigure 2. The Integrated Behavior Change (IBC) Model and the Intention-Behavior Gap

eFigure 3. Acyclic Behavior Change Diagram

eFigure 4. Weekly Moderate to Vigorous Physical Activity (MVPA) by Treatment Group for Week 21 Sample

eFigure 5. Weekly Moderate to Vigorous Physical Activity (MVPA) by Treatment Group for Week 61 Sample

eFigure 6. Treatment Effects on Moderate to Vigorous Physical Activity (MVPA) Relative to Control: Baseline Week MVPA Interaction Analysis

eFigure 7. Effects on Moderate to Vigorous Physical Activity (MVPA) Relative to Control: Baseline Week MVPA Interaction Analysis (Intention-to-Treat Analysis)

eFigure 8. Treatment Effects on Psychological Process Variables Relative to the Control at Week 21

eFigure 9. Treatment Effects on Psychological Process Variables Relative to the Control at Week 61

eFigure 10. Box Plots of Time Spent on Each Module in Each Intervention Week

eFigure 11. Box Plots of Time Spent on All Modules in Each Intervention Week

eFigure 12. Satisfaction Survey Results

eTable 1. Schematic Overview of Data Collection During the Trial

eTable 2. Week 9 Between-Group Attrition Check: Test of Differences in Mean Baseline Characteristics of Nonattritors at Week 9 Between Groups

eTable 3. Week 21 Between-Group Attrition Check: Test of Differences in Mean Baseline Characteristics of Nonattritors at Week 21 Between Groups

eTable 4. Week 61 Between-Group Attrition Check: Test of Differences in Mean Baseline Characteristics of Nonattritors at Week 61 Between Groups

eTable 5. Within-Group Attrition Check: Test of Differences Between Mean Baseline Characteristics of Baseline Sample (Includes Attritors) and Week 9 Sample (Excludes Attritors) by Treatment Group

eTable 6. Within-Group Attrition Check: Test of Differences Between Mean Baseline Characteristics of Baseline Sample (Includes Attritors) and Week 21 Sample (Excludes Attritors) by Treatment Group

© 2025 Kókai LL et al. *JAMA Network Open*.

eTable 7. Within-Group Attrition Check: Test of Differences Between Mean Baseline Characteristics of Baseline Sample (Includes Attritors) and Week 61 Sample (Excludes Attritors) by Treatment Group

eTable 8. Module Completion Compliers by Module (Sample of Nonattritors)

eMethods. Appropriateness of OLS Regression Model

eFigure 13. Plots of Observed vs Fitted Values for Moderate to Vigorous Physical Activity (MVPA) Regressions

eFigure 14. Distribution of Residuals in Moderate to Vigorous Physical Activity (MVPA) Regressions

eAppendix. i2be App Overview and Module Scripts

eReferences

This supplemental material has been provided by the authors to give readers additional information about their work.

© 2025 Kókai LL et al. *JAMA Network Open*.

eFigure 1. Study Timeline

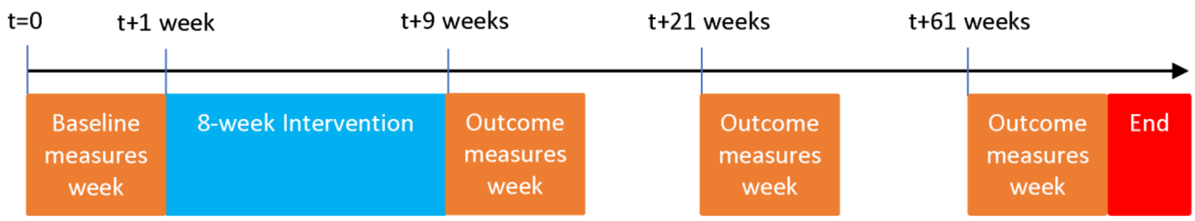

eFigure 2. The Integrated Behavior Change (IBC) Model and the Intention-Behavior Gap

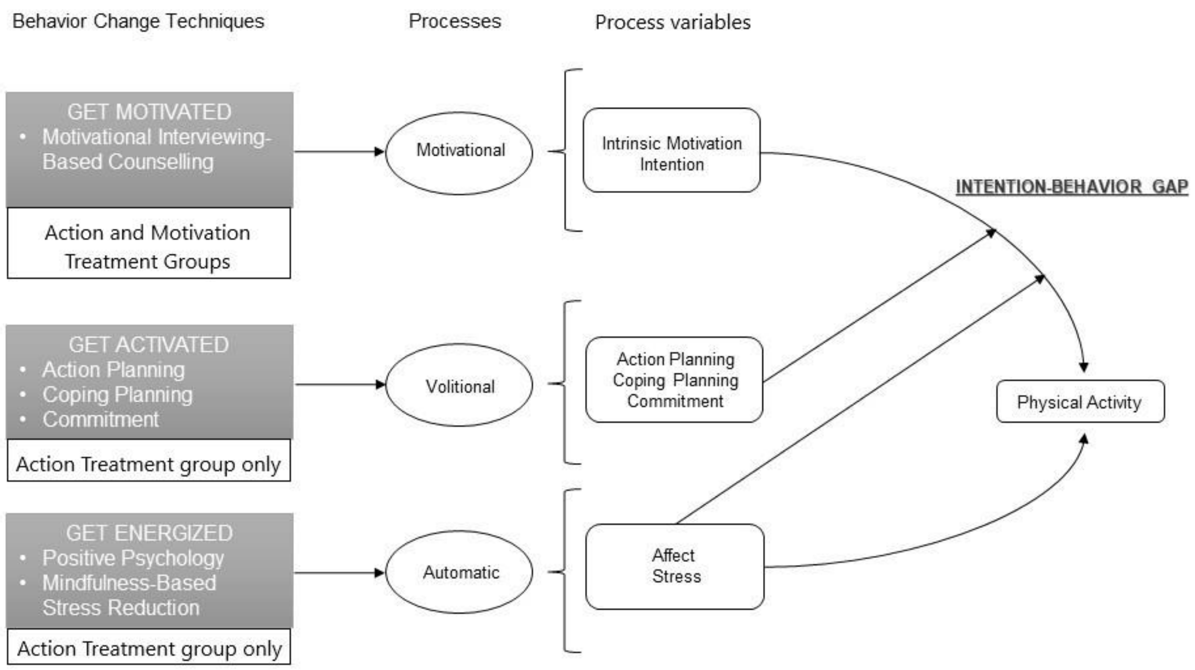

Notes: This figure was previously published as Figure 1 in the protocol for this study (Kókai et al., 2022).

© 2025 Kókai LL et al. *JAMA Network Open*.

eFigure 3. Acyclic Behavior Change Diagram

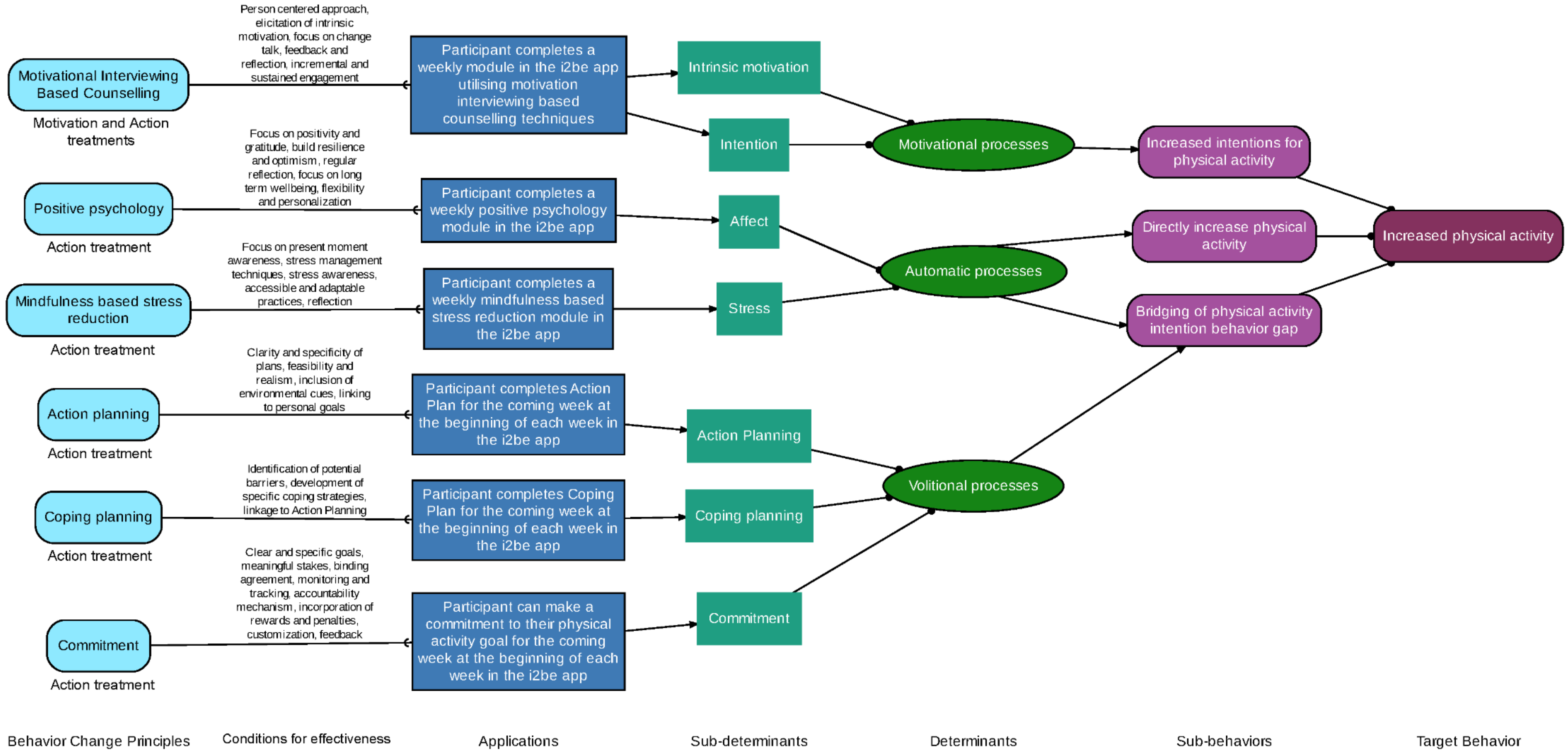

Notes: Acyclic Behavior Change Diagram created following Metz et al. (2022) and using the app provided by the authors of that paper: https://matherion.shinyapps.io/ABCD---Shiny-App/

© 2025 Kókai LL et al. *JAMA Network Open*.

eFigure 4. Weekly Moderate to Vigorous Physical Activity (MVPA) by Treatment Group for Week 21 Sample

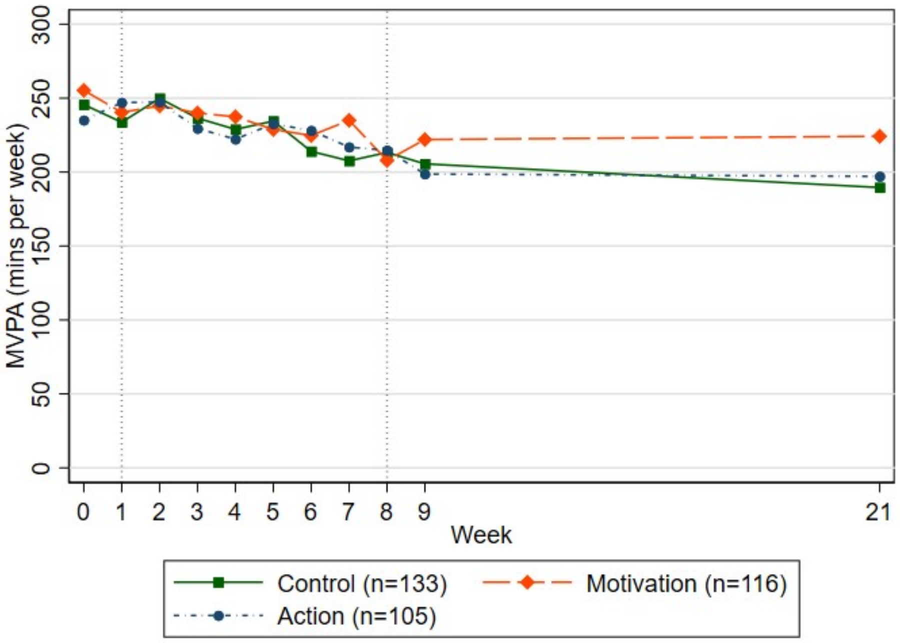

Notes: Graph shows mean weekly MVPA (minutes) for sample of participants for whom MVPA data for each of weeks 0-9 and week 21 is non-missing (n=350).

eFigure 5. Weekly Moderate to Vigorous Physical Activity (MVPA) by Treatment Group for Week 61 Sample

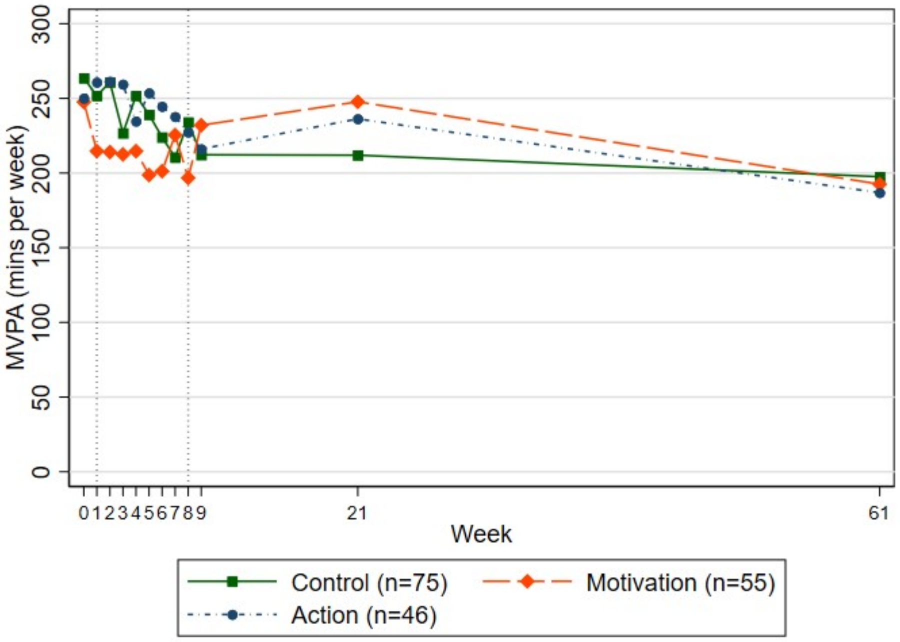

Notes: Graph shows mean weekly MVPA (minutes) for sample of participants for whom MVPA data for each of weeks 0-9, week 21 and week 61 is non-missing (n=135).

© 2025 Kókai LL et al. *JAMA Network Open*.

eFigure 6. Treatment Effects on Moderate to Vigorous Physical Activity (MVPA) Relative to Control: Baseline Week MVPA Interaction Analysis

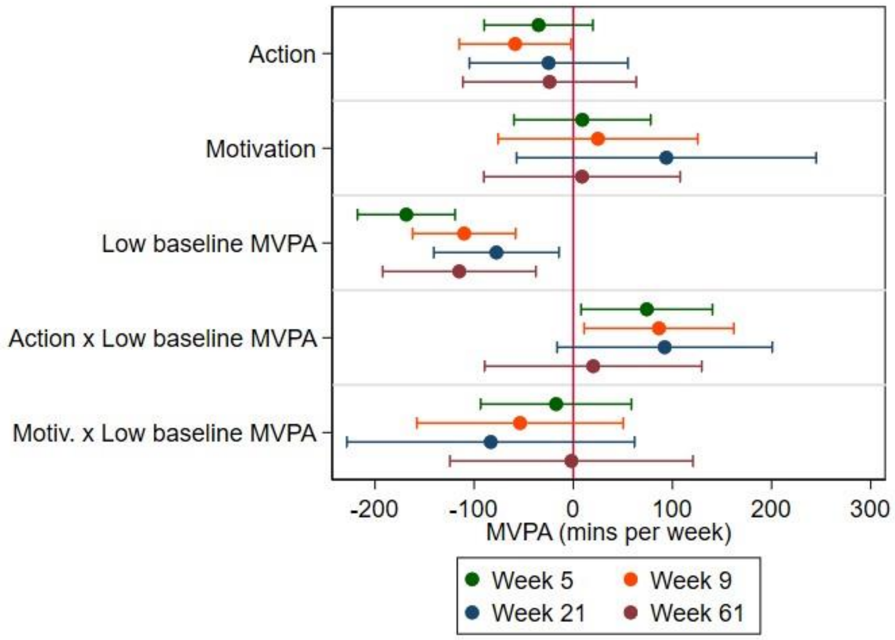

Notes: Linear regression of MVPA at weeks 5, 9, 21 and 61 on indicators for being in Motivation and Action, an indicator for having low baseline week MVPA (i.e., baseline week MVPA at or below the median (210 mins)), and interaction terms for the interactions of each of the Motivation and Action indicators with the low baseline week MVPA indicator. Control variables are baseline week MVPA, age, trait self-control, habit, household composition, education, type of prior hypertensive pregnancy disorder. Graph shows treatment effect estimates with 95% confidence intervals.

eFigure 7. Effects on Moderate to Vigorous Physical Activity (MVPA) Relative to Control: Baseline Week MVPA Interaction Analysis (Intention-to-Treat Analysis)

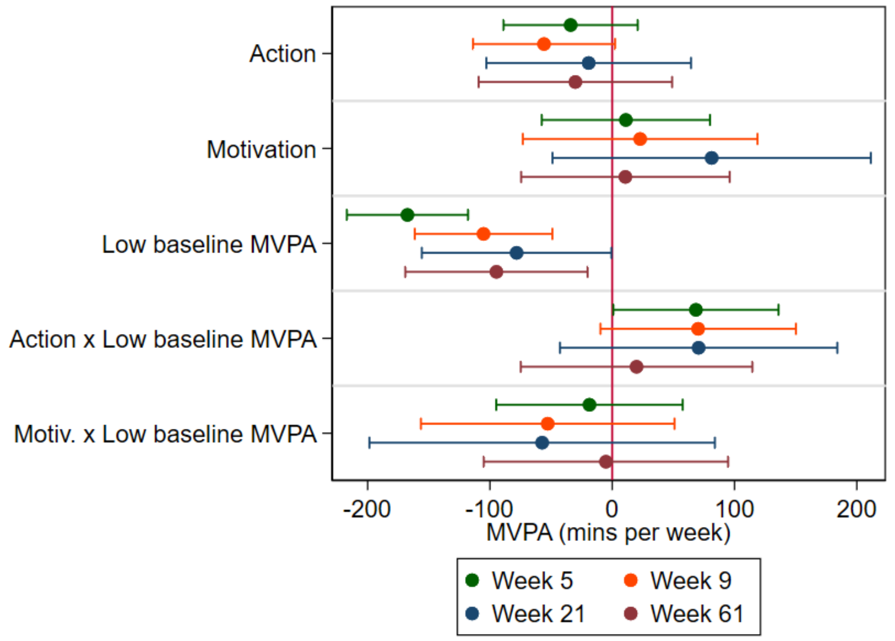

Notes: The same regression as the previous figure, except carried out on intention-to-treat basis (n=610). Multiple Imputation by Chained Equations used to impute missing dependent variable values.

© 2025 Kókai LL et al. *JAMA Network Open*.

eFigure 8. Treatment Effects on Psychological Process Variables Relative to the Control at Week 21

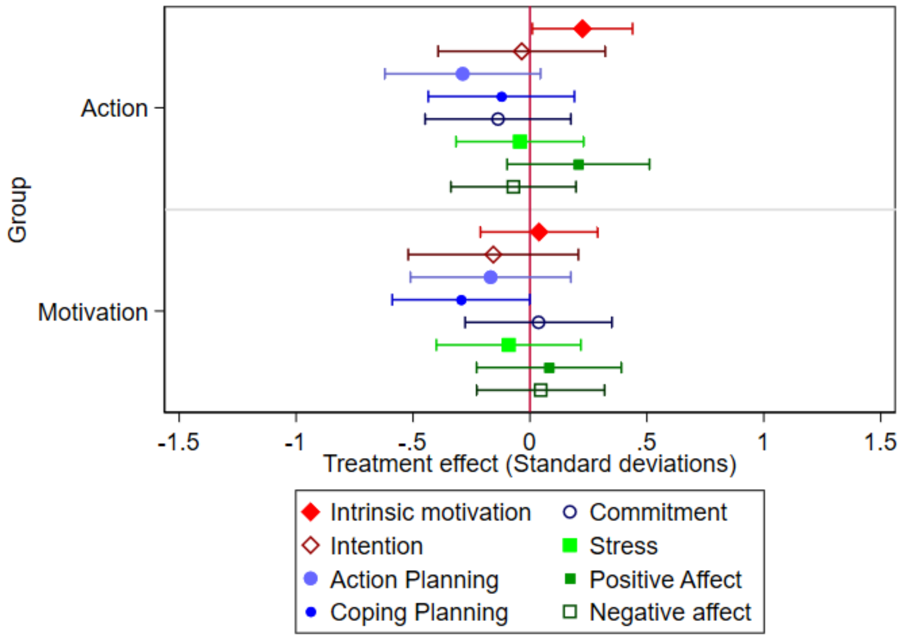

Notes: Linear regression of standardized psychological process variables at week 21 on indicators for being in Motivation and Action. Control variables are baseline MVPA, age, trait self-control, habit, household composition, education, type of prior hypertensive pregnancy disorder. Graph shows treatment effect estimates with 95% confidence intervals.

eFigure 9. Treatment Effects on Psychological Process Variables Relative to the Control at Week 61

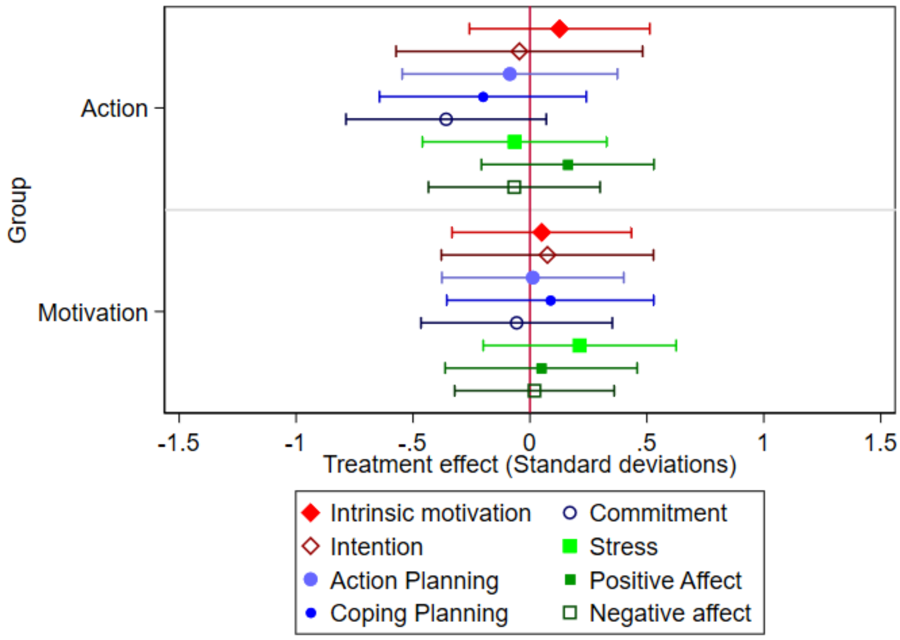

Notes: Linear regression of standardized psychological process variables at week 61 on indicators for being in Motivation and Action. Control variables are baseline MVPA, age, trait self-control, habit, household composition, education, type of prior hypertensive pregnancy disorder. Graph shows treatment effect estimates with 95% confidence intervals.

© 2025 Kókai LL et al. *JAMA Network Open*.

eFigure 10. Box Plots of Time Spent on Each Module in Each Intervention Week

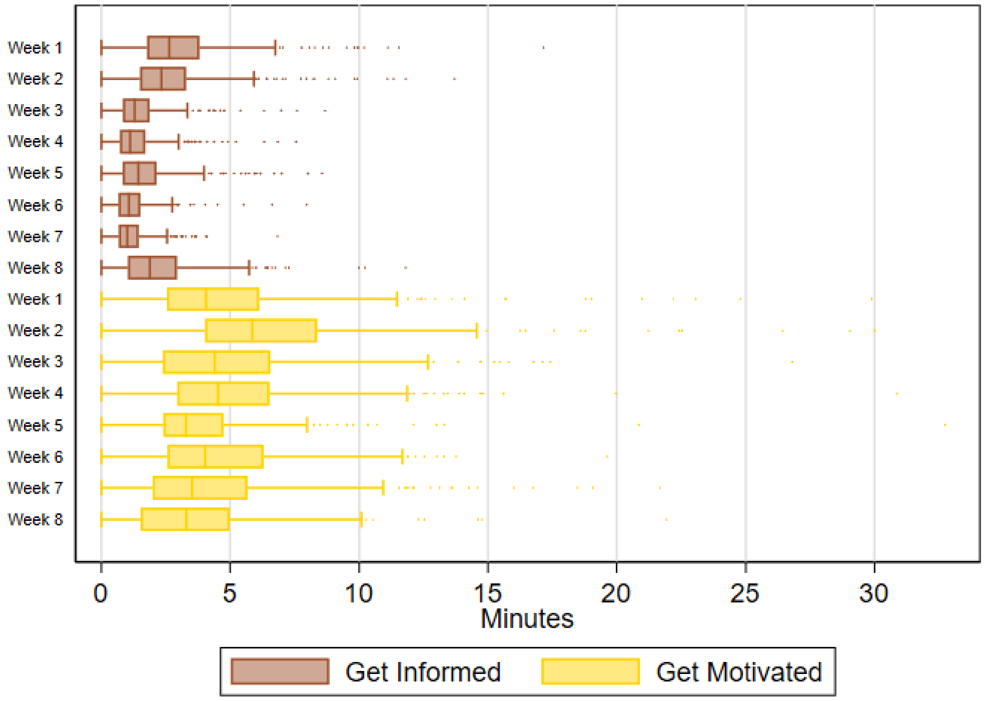

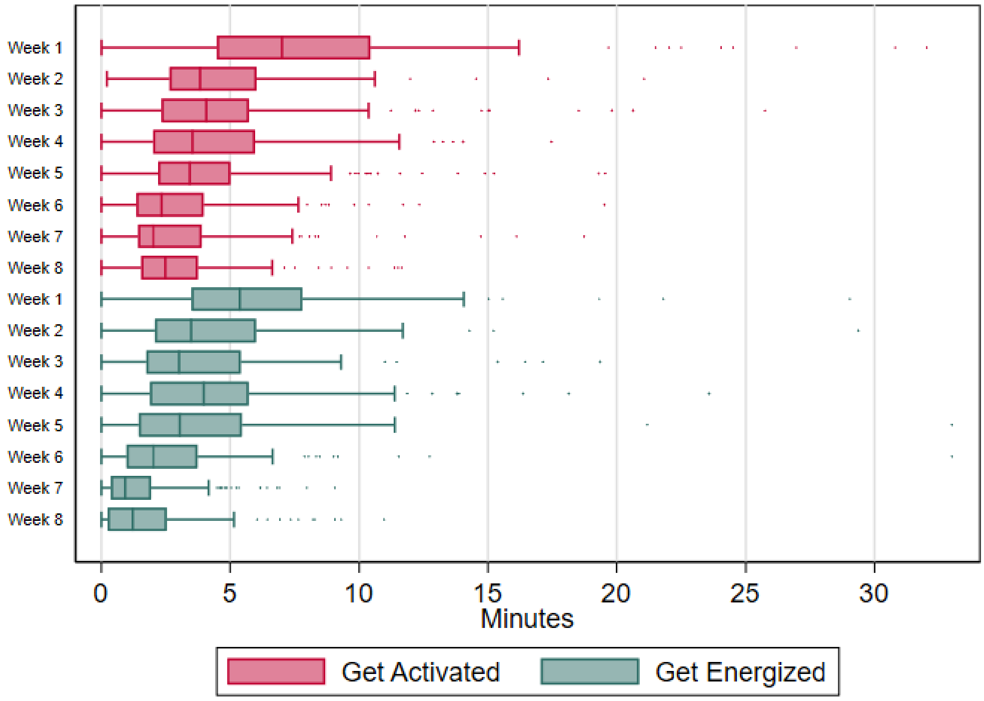

Notes: Sample is all those participants for whom MVPA data was recorded in week 9. Data winsorized at 33 minutes for clearer graphs (there were only two observations above this cut-off).

© 2025 Kókai LL et al. *JAMA Network Open*.

eFigure 11. Box Plots of Time Spent on All Modules in Each Intervention Week

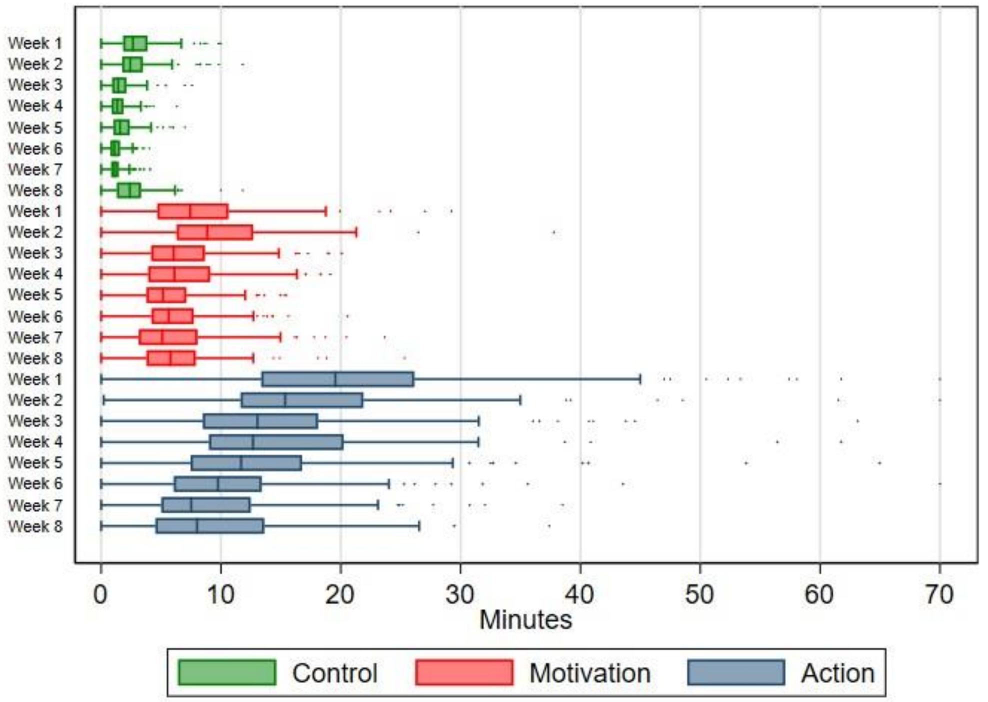

Notes: Sample is all those participants for whom MVPA data was recorded in week 9. Data winsorized at 70 minutes for clearer graphs (there were only three observations above this cut-off).

© 2025 Kókai LL et al. *JAMA Network Open*.

eFigure 12. Satisfaction Survey Results

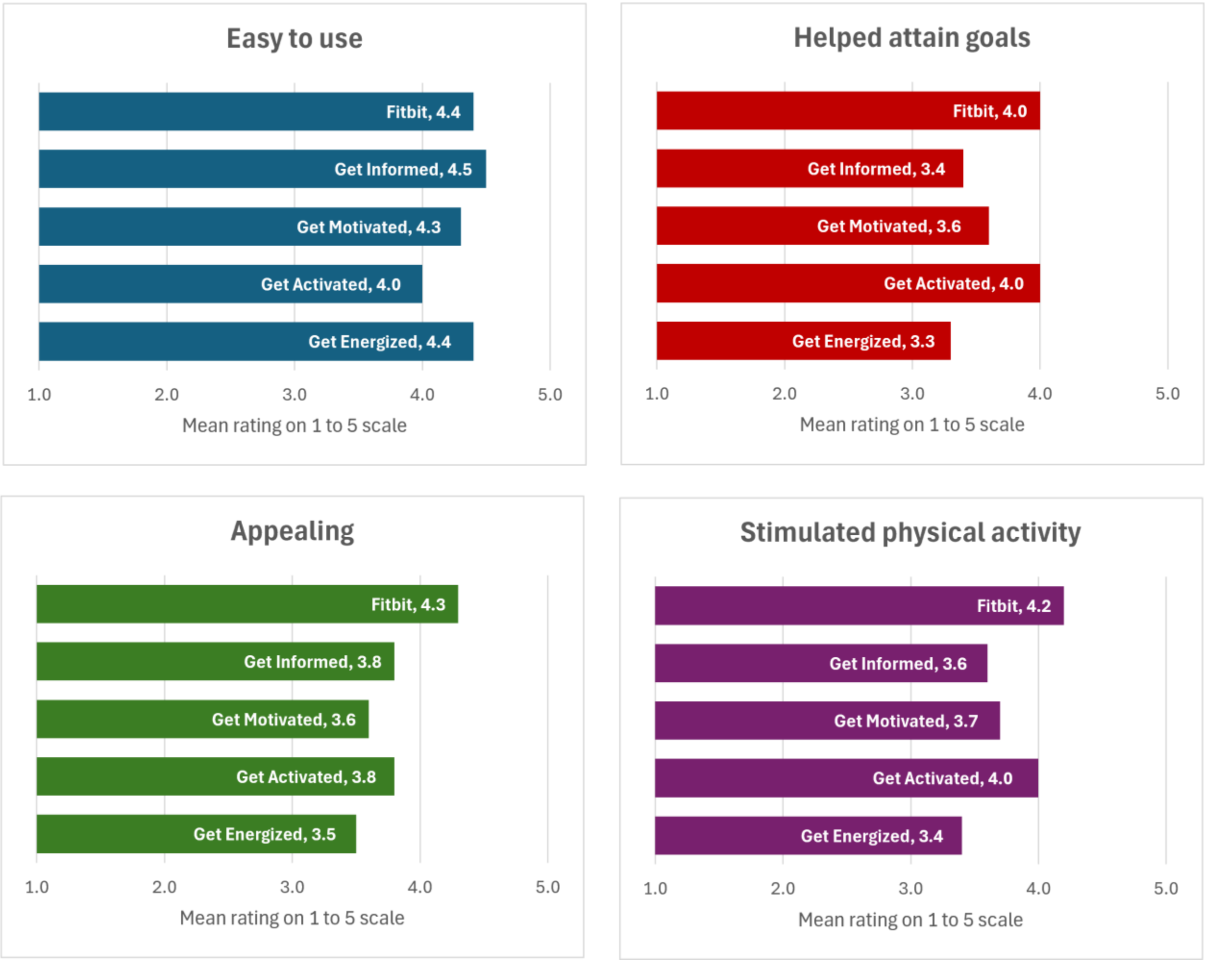

Notes: Results of satisfaction survey given to participants in week 9. Shows mean rating on a scale of 1-5 given by participants to the Fitbit and each of the four i2be modules on four key criteria. Sample sizes are 367 (Fitbit), 348 (Get Informed), 212 (Get Motivated), 99 (Get Activated), and 99 (Get Energized).

© 2025 Kókai LL et al. *JAMA Network Open*.

eTable 1. Schematic Overview of Data Collection During the Trial

Outcomes	Variables	Measurements	Baseline	Follow-up*
**Primary Outcome**				
Objectively measured by Fitbit	Physical activity**	Moderate-to-vigorous intensity physical activity (MVPA) (min / week)	✓	✓
**Secondary Outcomes**				
Objectively measured by Fitbit	Heart rate**	Week mean of daily resting heart rate (beats / min)	✓	✓
Self-reported into app	Body mass index	Weight / length^2 (kg / m^2)	✓	✓
	Waist-hip ratio	Waist circumference/ hip circumference	✓	✓
	Cardiorespiratory fitness	1 mile Rockport walk test	✓	✓
	Subjective well-being	Satisfaction with Life Scale	✓	✓
**Tertiary Outcomes (psychological process variables)**				
Self-reported into app	Motivation	The Behavioral Regulation in Exercise Questionnaire	✓	✓
	Intention	Own design based on Ajzen guidelines †	✓	✓
	Action planning	Own design based on Sniehotta measure ‡	✓	✓
	Coping planning	Own design based on Sniehotta measure ‡	✓	✓
	Commitment	Own design	✓	✓
	Affect	Global Mood Scale	✓	✓
	Stress	Perceived Stress Scale	✓	✓
**Control variables**				
Self-reported into app	Trait self-control	Brief Self-control Scale	✓	
	Habit	Habit Strength	✓	
	Age	Age (years)	✓	
	Education	English version based on ISCED 2011<br>Dutch version based on SOI 2016	✓	
	Household composition	Living situation (Partner, children)	✓	
	Type of disorder	Type of hypertensive pregnancy disorder	✓	
**Stratification variables**				
Self-reported into app	Time since giving birth***	< 12 months post-partum (Yes / No)	✓	
	MVPA	Mean weekly minutes of MVPA in the past month (Low / Mid / High)	✓	
**Process evaluation**				
Self-reported into app	Program acceptability	Component usability, appropriateness, engagement, appeal, satisfactions and dissatisfactions		✓****
Objectively measured by app	Program fidelity	Compliance with program	✓	✓
**Other variables**				
Self-reported into app	Lactation status	Currently lactating (Yes / No)	✓	
	Pregnancy status	Currently pregnant (Yes / No; Due date)	✓	✓
	Voucher preference	Choice from three sports store vouchers	✓	

Notes: This is a modified version of Table 2 previously published in the protocol for this study (Kókai et al., 2022).
* Follow-up measurements at week 9, week 21 and week 61.
** Also measured weekly for the duration of the eight-week intervention.
† Ajzen, Icek. Constructing a theory of planned behavior questionnaire. 2006.
‡ Sniehotta, Falko F., Ralf Schwarzer, Urte Scholz, and Benjamin Schüz. "Action planning and coping planning for long-term lifestyle change: theory and assessment." *European journal of social psychology* 35, no. 4 (2005): 565-576.
*** We stratified on this variable because we expected treatment effects to differ for individuals less than 12 months postpartum. This was based on several reasons: (1) they were more likely to have recently experienced a pre-eclamptic pregnancy, which could heighten their motivation to engage in MVPA to reduce long-term CVD risks; (2) having a child under one year might limit their ability to participate in MVPA due to caregiving demands; and (3) they were likely still in the process of postpartum weight loss, which could influence both their motivation and capacity for MVPA.
****Only measured at week 9.

© 2025 Kókai LL et al. *JAMA Network Open*.

eTable 2. Week 9 Between-Group Attrition Check: Test of Differences in Mean Baseline Characteristics of Nonattritors at Week 9 Between Groups

	(1) Obser-vations	(2) Full sample	(3) Con-trol	(4) Motiv-ation	(5) Action	(6) C vs M	(7) C vs A	(8) M vs A
Lower education	437	3%	4%	1%	3%	0.158	0.676	0.327
Mid-level education	437	29%	26%	31%	29%	0.338	0.550	0.743
Higher education	437	68%	70%	68%	68%	0.655	0.681	0.983
Living with partner	437	85%	84%	87%	86%	0.449	0.632	0.800
Living with child(ren)	437	95%	95%	96%	93%	0.796	0.423	0.295
Prior (pre)eclampsia	424	67%	70%	59%	71%	0.051	0.907	0.044
Prior HELLP	424	58%	61%	58%	54%	0.654	0.278	0.513
Other hypertensive preg. disorder	424	29%	26%	30%	31%	0.422	0.289	0.777
Lactating	427	8%	5%	10%	9%	0.110	0.149	0.902
<12 months postpartum	443	16%	13%	16%	18%	0.504	0.256	0.631
Age (years)	437	39.23	39.26	39.54	38.83	0.736	0.619	0.407
MVPA (Fitbit – mins/week)	435	242	237	254	233	0.482	0.842	0.394
Resting Heartrate (BPM)	424	66.7	66.3	67.2	66.6	0.321	0.735	0.500
BMI (kg/m^2)	427	26.1	26.2	25.9	26.1	0.567	0.805	0.764
Waist-Hip ratio	389	0.85	0.85	0.85	0.85	0.983	0.810	0.737
Cardiorespiratory. Fitness (ml/kg/min)	290	29.2	28.6	31.5	27.1	0.315	0.604	0.148
Subjective well-being (7-point scale)	400	5.03	4.93	5.13	5.06	0.140	0.347	0.618
Trait self-control (5-point scale)	404	3.19	3.12	3.29	3.16	0.042*	0.636	0.120
Habit (6-point scale)	374	3.56	3.56	3.55	3.56	0.944	0.993	0.956

Notes: Columns 2-5 show mean values of variables in the full sample and in each of the intervention groups among those for whom we have both the relevant baseline data and week 9 MVPA data (i.e., non-attritor). For the top panel, means of binary variables expressed as percentages. Columns 6-8 show p-values from t-tests of equality of means between Control (C), Motivation (M) and Action (A). * p < 0.05, ** p < 0.01, *** p < 0.001.

eTable 3. Week 21 Between-Group Attrition Check: Test of Differences in Mean Baseline Characteristics of Nonattritors at Week 21 Between Groups

	(1) Obser-vations	(2) Full sample	(3) Con-trol	(4) Motiv-ation	(5) Action	(6) C vs M	(7) C vs A	(8) M vs A
Lower education	355	2%	3%	1%	3%	0.221	0.883	0.286
Mid-level education	355	29%	26%	32%	29%	0.323	0.583	0.681
Higher education	355	69%	71%	67%	68%	0.552	0.632	0.918
Living with partner	355	86%	85%	87%	87%	0.584	0.577	0.985
Living with child(ren)	355	95%	96%	97%	93%	0.869	0.237	0.196
Prior (pre)eclampsia	344	67%	70%	63%	66%	0.272	0.564	0.621
Prior HELLP	344	58%	60%	60%	54%	0.936	0.364	0.416
Other hypertensive preg. disorder	344	28%	28%	28%	30%	0.942	0.706	0.765
Lactating	346	8%	4%	12%	10%	0.029*	0.063	0.754
<12 months postpartum	359	14%	12%	15%	17%	0.529	0.309	0.702
Age (years)	355	39.52	39.05	39.95	39.61	0.328	0.554	0.731
MVPA (Fitbit – mins/week)	355	246	247	255	235	0.729	0.590	0.403
Resting Heartrate (BPM)	347	67.0	66.7	67.2	67.1	0.621	0.688	0.914
BMI (kg/m^2)	349	25.9	26.0	25.7	26.1	0.644	0.933	0.614
Waist-Hip ratio	322	0.85	0.85	0.85	0.86	0.930	0.576	0.377
Cardiorespiratory. Fitness (ml/kg/min)	244	29.5	29.3	31.4	27.6	0.512	0.573	0.243
Subjective well-being (7-point scale)	331	5.05	4.9	5.19	5.07	0.053	0.251	0.464
Trait self-control (5-point scale)	333	3.21	3.15	3.34	3.16	0.040*	0.892	0.061
Habit (6-point scale)	311	3.55	3.58	3.52	3.56	0.526	0.826	0.726

Notes: Columns 2-5 show mean values of variables in the full sample and in each of the intervention groups among those for whom we have both the relevant baseline data and week 21 MVPA data (i.e., non-attritor). For the top panel, means of binary variables expressed as percentages. Columns 6-8 show p-values from t-tests of equality of means between Control (C), Motivation (M) and Action (A). * p < 0.05, ** p < 0.01, *** p < 0.001.

eTable 4. Week 61 Between-Group Attrition Check: Test of Differences in Mean Baseline Characteristics of Nonattritors at Week 61 Between Groups

	(1) Obser-vations	(2) Full sample	(3) Con-trol	(4) Motiv-ation	(5) Action	(6) C vs M	(7) C vs A	(8) M vs A
Lower education	177	3%	4%	2%	2%	0.473	0.554	0.923
Mid-level education	177	26%	27%	29%	21%	0.798	0.442	0.341
Higher education	177	71%	69%	69%	77%	0.983	0.330	0.368
Living with partner	177	89%	85%	89%	94%	0.515	0.147	0.409
Living with child(ren)	177	95%	95%	98%	92%	0.300	0.527	0.127
Prior (pre)eclampsia	173	67%	76%	63%	58%	0.114	0.041	0.637
Prior HELLP	173	57%	59%	48%	65%	0.224	0.555	0.097
Other hypertensive preg. disorder	173	25%	23%	31%	21%	0.265	0.827	0.228
Lactating	173	7%	4%	11%	6%	0.142	0.624	0.393
<12 months postpartum	178	15%	13%	15%	17%	0.845	0.613	0.770
Age (years)	177	39.42	39.46	39.45	39.33	0.997	0.927	0.928
MVPA (Fitbit – mins/week)	177	254	264	248	246	0.630	0.619	0.976
Resting Heartrate (BPM)	172	66.9	67.3	67.2	65.8	0.904	0.272	0.374
BMI (kg/m^2)	174	25.8	25.9	26.3	25.1	0.693	0.353	0.261
Waist-Hip ratio	164	0.85	0.85	0.86	0.85	0.508	0.990	0.359
Cardiorespiratory. Fitness (ml/kg/min)	128	29.7	29.0	31.3	29.1	0.527	0.992	0.553
Subjective well-being (7-point scale)	169	5.2	5.06	5.33	5.26	0.171	0.332	0.737
Trait self-control (5-point scale)	169	3.25	3.2	3.34	3.22	0.258	0.888	0.348
Habit (6-point scale)	159	3.55	3.57	3.45	3.65	0.371	0.591	0.203

Notes: Columns 2-5 show mean values of variables in the full sample and in each of the intervention groups among those for whom we have both the relevant baseline data and week 61 MVPA data (i.e., non-attritor). For the top panel, means of binary variables expressed as percentages. Columns 6-8 show p-values from t-tests of equality of means between Control (C), Motivation (M) and Action (A). * p < 0.05, ** p < 0.01, *** p < 0.001.

© 2025 Kókai LL et al. *JAMA Network Open*.

eTable 5. Within-Group Attrition Check: Test of Differences Between Mean Baseline Characteristics of Baseline Sample (Includes Attritors) and Week 9 Sample (Excludes Attritors) by Treatment Group

	Full Sample			Control			Motivation			Action		
	(1) Baseline	(2) Week 9	(3) P-value	(4) Baseline	(5) Week 9	(6) P-value	(7) Baseline	(8) Week 9	(9) P-value	(10) Baseline	(11) Week 9	(12) P-value
Lower education	3%	3%	0.886	4%	4%	0.894	2%	1%	0.874	3%	3%	0.847
Mid-level education	31%	29%	0.565	28%	26%	0.762	31%	31%	0.999	33%	29%	0.509
Higher education	67%	68%	0.608	69%	70%	0.813	67%	68%	0.968	65%	68%	0.563
Living with partner	85%	85%	0.789	83%	84%	0.936	87%	87%	0.996	84%	86%	0.697
Living with child(ren)	95%	95%	0.794	96%	95%	0.665	96%	96%	0.960	94%	93%	0.882
Prior (pre)eclampsia	69%	67%	0.448	73%	70%	0.552	65%	59%	0.340	69%	71%	0.769
Prior HELLP	56%	58%	0.524	56%	61%	0.407	57%	58%	0.859	54%	54%	0.957
Other hypertensive preg. disorder	30%	29%	0.709	26%	26%	0.904	32%	30%	0.692	32%	31%	0.945
Lactating	7%	8%	0.700	4%	5%	0.685	10%	10%	0.872	7%	9%	0.559
<12 months postpartum	15%	16%	0.826	15%	13%	0.698	15%	16%	0.813	16%	18%	0.568
Age (years)	38.9	39.2	0.456	39.0	39.3	0.737	39.0	39.5	0.497	38.6	38.8	0.811
MVPA (Fitbit – mins/week)	235	242	0.619	227	237	0.590	261	254	0.771	218	233	0.396
Resting Heartrate (BPM)	66.66	66.66	0.999	66.47	66.27	0.818	67.17	67.15	0.974	66.33	66.56	0.774
BMI (kg/m^2)	26.4	26.1	0.353	26.7	26.2	0.455	26.5	25.9	0.366	26.1	26.1	0.963
Waist-Hip ratio	0.86	0.85	0.641	0.86	0.85	0.730	0.86	0.85	0.509	0.85	0.85	0.667
Cardiorespiratory. Fitness (ml/kg/min)	28.7	29.2	0.804	28.4	28.6	0.962	31.7	31.5	0.953	25.7	27.1	0.656
Subjective well-being (7-point scale)	5.02	5.03	0.819	4.92	4.93	0.963	5.09	5.13	0.762	5.05	5.06	0.939
Trait self-control (5-point scale)	3.19	3.19	0.923	3.15	3.12	0.787	3.25	3.29	0.589	3.17	3.16	0.877
Habit (6-point scale)	3.53	3.56	0.572	3.55	3.56	0.875	3.51	3.55	0.604	3.53	3.56	0.777
Observations	481	443	-	172	159	-	157	151	-	152	133	-

Notes: Columns (1), (4), (7), (10) show the mean of each variable for all participants with non-missing values for that variable at baseline for the full sample, Control, Motivation and Action respectively. Columns (2), (5), (8), (11) shows the same only for participants who have non-missing values for MVPA at week 9 (i.e., non-attritors). For the top panel, means of binary variables expressed as percentages. Columns (3), (6), (9), (12) show the p-value for t-test of baseline mean = week 9 mean. * $p < 0.05$, ** $p < 0.01$, *** $p < 0.001$. In the final row, the total observations at baseline and week 9 for which MVPA data is not missing are shown.

© 2025 Kókai LL et al. *JAMA Network Open*.

eTable 6. Within-Group Attrition Check: Test of Differences Between Mean Baseline Characteristics of Baseline Sample (Includes Attritors) and Week 21 Sample (Excludes Attritors) by Treatment Group

	Full Sample			Control			Motivation			Action		
	(1) Baseline	(2) Week 21	(3) P-value	(4) Baseline	(5) Week 21	(6) P-value	(7) Baseline	(8) Week 21	(9) P-value	(10) Baseline	(11) Week 21	(12) P-value
Lower education	3%	2%	0.741	4%	3%	0.784	2%	1%	0.613	3%	3%	0.956
Mid-level education	31%	29%	0.630	28%	26%	0.775	31%	32%	0.887	33%	29%	0.539
Higher education	67%	69%	0.562	69%	71%	0.700	67%	67%	0.985	65%	68%	0.560
Living with partner	85%	86%	0.545	83%	85%	0.760	87%	87%	0.933	84%	87%	0.479
Living with child(ren)	95%	95%	0.939	96%	96%	0.926	96%	97%	0.779	94%	93%	0.743
Prior (pre)eclampsia	69%	67%	0.442	73%	70%	0.517	65%	63%	0.808	69%	66%	0.588
Prior HELLP	56%	58%	0.481	56%	60%	0.485	57%	60%	0.670	54%	54%	0.959
Other hypertensive preg. disorder	30%	28%	0.659	26%	28%	0.768	32%	28%	0.502	32%	30%	0.756
Lactating	7%	8%	0.473	4%	4%	0.938	10%	12%	0.693	7%	10%	0.388
<12 months postpartum	15%	14%	0.711	15%	12%	0.480	15%	15%	0.938	16%	17%	0.845
Age (years)	38.9	39.5	0.195	39.0	39.1	0.943	39.0	40.0	0.266	38.6	39.6	0.274
MVPA (Fitbit – mins/week)	235	246	0.403	227	247	0.319	261	255	0.821	218	235	0.377
Resting Heartrate (BPM)	66.66	66.95	0.584	66.47	66.67	0.813	67.17	67.16	0.987	66.33	67.05	0.398
BMI (kg/m^2)	26.4	25.9	0.198	26.7	26.0	0.267	26.5	25.7	0.264	26.1	26.1	0.997
Waist-Hip ratio	0.86	0.85	0.846	0.86	0.85	0.776	0.86	0.85	0.519	0.85	0.86	0.320
Cardiorespiratory. Fitness (ml/kg/min)	28.7	29.5	0.687	28.4	29.3	0.806	31.7	31.4	0.942	25.7	27.6	0.560
Subjective well-being (7-point scale)	5.02	5.05	0.714	4.92	4.9	0.883	5.09	5.19	0.471	5.05	5.07	0.847
Trait self-control (5-point scale)	3.19	3.21	0.591	3.15	3.15	0.955	3.25	3.34	0.302	3.17	3.16	0.885
Habit (6-point scale)	3.53	3.55	0.628	3.55	3.58	0.678	3.51	3.52	0.890	3.53	3.56	0.785
Observations	481	359	-	172	134	-	157	116	-	152	109	-

Notes: Columns (1), (4), (7), (10) show the mean of each variable for all participants with non-missing values for that variable at baseline for the full sample, Control, Motivation and Action respectively. Columns (2), (5), (8), (11) shows the same only for participants who have non-missing values for MVPA at Week 21 (i.e., non-attritors). For the top panel, means of binary variables expressed as percentages. Columns (3), (6), (9), (12) show the p-value for t-test of baseline mean = Week 21 mean. * $p < 0.05$, ** $p < 0.01$, *** $p < 0.001$. In the final row, the total observations at baseline and Week 21 for which MVPA data is not missing are shown.

© 2025 Kókai LL et al. *JAMA Network Open*.

eTable 7. Within-Group Attrition Check: Test of Differences Between Mean Baseline Characteristics of Baseline Sample (Includes Attritors) and Week 61 Sample (Excludes Attritors) by Treatment Group

	Full Sample			Control			Motivation			Action		
	(1) Baseline	(2) Week 61	(3) P-value	(4) Baseline	(5) Week 61	(6) P-value	(7) Baseline	(8) Week 61	(9) P-value	(10) Baseline	(11) Week 61	(12) P-value
Lower education	3%	3%	0.871	4%	4%	0.876	2%	2%	0.881	3%	2%	0.826
Mid-level education	31%	26%	0.250	28%	27%	0.925	31%	29%	0.774	33%	21%	0.108
Higher education	67%	71%	0.286	69%	69%	0.979	67%	69%	0.808	65%	77%	0.100
Living with partner	85%	89%	0.190	83%	85%	0.722	87%	89%	0.645	84%	94%	0.086
Living with child(ren)	95%	95%	0.825	96%	95%	0.520	96%	98%	0.427	94%	92%	0.626
Prior (pre)eclampsia	69%	67%	0.625	73%	76%	0.659	65%	63%	0.831	69%	58%	0.147
Prior HELLP	56%	57%	0.731	56%	59%	0.662	57%	48%	0.243	54%	65%	0.186
Other hypertensive preg. disorder	30%	25%	0.204	26%	23%	0.558	32%	31%	0.971	32%	21%	0.144
Lactating	7%	7%	0.949	4%	4%	0.884	10%	11%	0.822	7%	6%	0.809
<12 months postpartum	15%	15%	0.876	15%	13%	0.784	15%	15%	0.937	16%	17%	0.864
Age (years)	38.9	39.4	0.385	39.0	39.5	0.646	39.0	39.5	0.683	38.6	39.3	0.557
MVPA (Fitbit – mins/week)	235	254	0.260	227	264	0.134	261	248	0.696	218	246	0.285
Resting Heartrate (BPM)	66.66	66.89	0.729	66.47	67.34	0.402	67.17	67.17	0.999	66.33	65.84	0.665
BMI (kg/m^2)	26.4	25.8	0.192	26.7	25.9	0.273	26.5	26.3	0.825	26.1	25.1	0.236
Waist-Hip ratio	0.86	0.85	0.727	0.86	0.85	0.639	0.86	0.86	0.936	0.85	0.85	0.845
Cardiorespiratory. Fitness (ml/kg/min)	28.7	29.7	0.649	28.4	29.0	0.879	31.7	31.3	0.927	25.7	29.1	0.415
Subjective well-being (7-point scale)	5.02	5.2	0.072	4.92	5.06	0.390	5.09	5.33	0.177	5.05	5.26	0.253
Trait self-control (5-point scale)	3.19	3.25	0.328	3.15	3.2	0.575	3.25	3.34	0.422	3.17	3.22	0.675
Habit (6-point scale)	3.53	3.55	0.689	3.55	3.57	0.795	3.51	3.45	0.671	3.53	3.65	0.361
Observations	481	178	-	172	75	-	157	55	-	152	48	-

Notes: Columns (1), (4), (7), (10) show the mean of each variable for all participants with non-missing values for that variable at baseline for the full sample, Control, Motivation and Action respectively. Columns (2), (5), (8), (11) shows the same only for participants who have non-missing values for MVPA at Week 61 (i.e., non-attritors). For the top panel, means of binary variables expressed as percentages. Columns (3), (6), (9), (12) show the p-value for t-test of baseline mean = Week 61 mean. * $p < 0.05$, ** $p < 0.01$, *** $p < 0.001$. In the final row, the total observations at baseline and Week 61 for which MVPA data is not missing are shown.

© 2025 Kókai LL et al. *JAMA Network Open*.

eTable 8. Module Completion Compliers by Module (Sample of Nonattritors)

	Total	Compliers	Proportion of compliers
Get Informed Module			
Full Sample	443	294	0.664
Control group	159	115	0.723
Motivation group	151	91	0.603
Action group	133	88	0.662
Get Motivated Module			
Motiv. & Action groups	284	218	0.768
Motivation group	151	123	0.815
Action group	133	95	0.714
Get Activated			
Action group	133	88	0.662
Get Energized			
Action group	133	84	0.632

Notes: Sample is all those who have a non-missing week 9 value for MVPA. Module completion compliance is defined as completing at least 75% of a given module in at least 7 weeks of the 8-week program.

© 2025 Kókai LL et al. *JAMA Network Open*.

eMethods. Appropriateness of OLS Regression Model

We used ordinary least squares regression (OLS) in our primary analysis, where the independent variables of interest (i.e. the treatment dummies) are exogenous by randomization. Here we consider the appropriateness of our use of OLS given key assumptions underlying OLS in relation to linearity, homoscedasticity, normality of the error distribution, multicollinearity, and autocorrelation.

Linearity: eFigure 13 shows plots of observed vs fitted values for each of the regressions in our primary analysis. We see no evidence here of substantial non-linearity.

Homoscedasticity: eFigure 13 shows some evidence of heteroscedasticity. This does not affect our point estimates, but may influence the standard errors. Therefore, we use Huber–White standard errors in our regressions, which allow for the fitting of an OLS model with heteroscedasticity.

Normality of error term: eFigure 14 shows that the residuals in the regressions approximate well a normal distribution.

Multicollinearity: Variance Inflation Factors (VIF) for each regression show that multicollinearity is not an issue. The VIF for the treatment dummies is <1.5 for the week 5, 9 and 21 regressions, and <1.7 for the week 61 regression. The VIF for the control variables, while not that important in our case as we are not interpreting coefficient estimates for the control variables, are still in an acceptable range. For the week 5, 9 and 21 regressions, the VIF for all control variables is below 2.3 except for the dummies for having a missing value for the age and household composition variables, which are below the commonly-used rule of thumb threshold of 5 and so still in an acceptable range. For week 61 regression, all VIFs for control variables are below 3.

Autocorrelation: As the regression models we run use cross-sectional data (i.e. each participant has only one observation at the same timepoint) autocorrelation is not an issue.

© 2025 Kókai LL et al. *JAMA Network Open*.

eFigure 13. Plots of Observed vs Fitted Values for Moderate to Vigorous Physical Activity (MVPA) Regressions

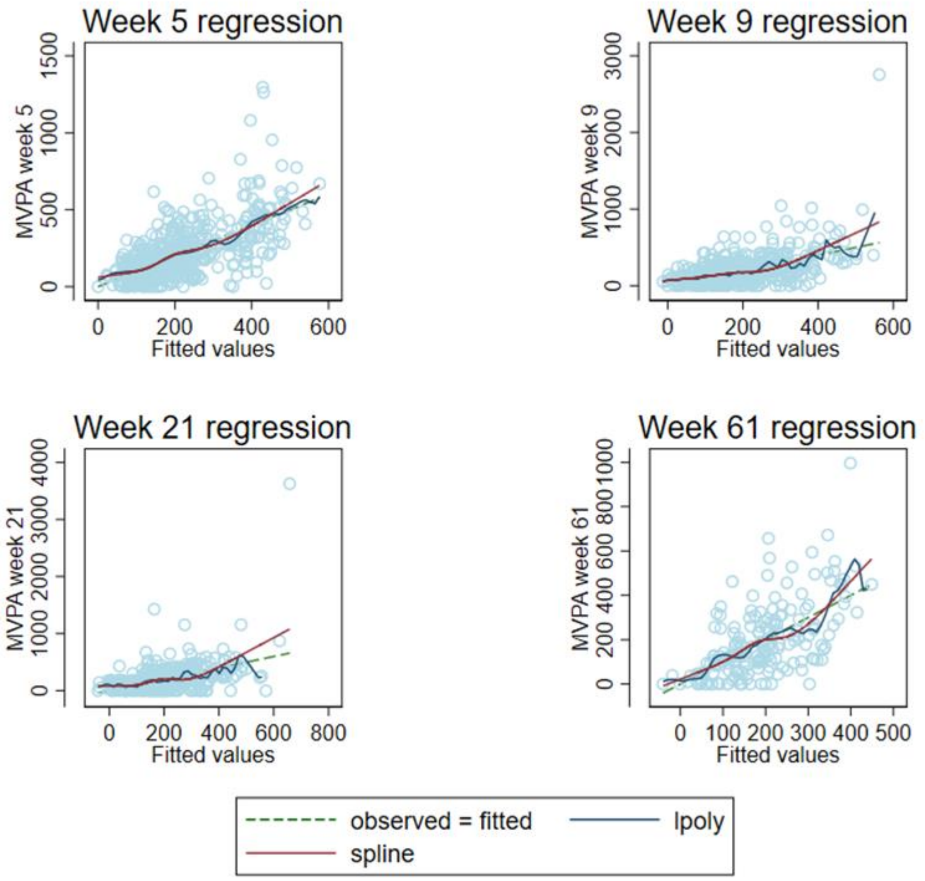

Notes: Lpoly is a trace of local polynomial smoothing of observed given predicted. Spline is a trace of restricted cubic spline smoothing of observed given predicted.

© 2025 Kókai LL et al. *JAMA Network Open*.

eFigure 14. Distribution of Residuals in Moderate to Vigorous Physical Activity (MVPA) Regressions

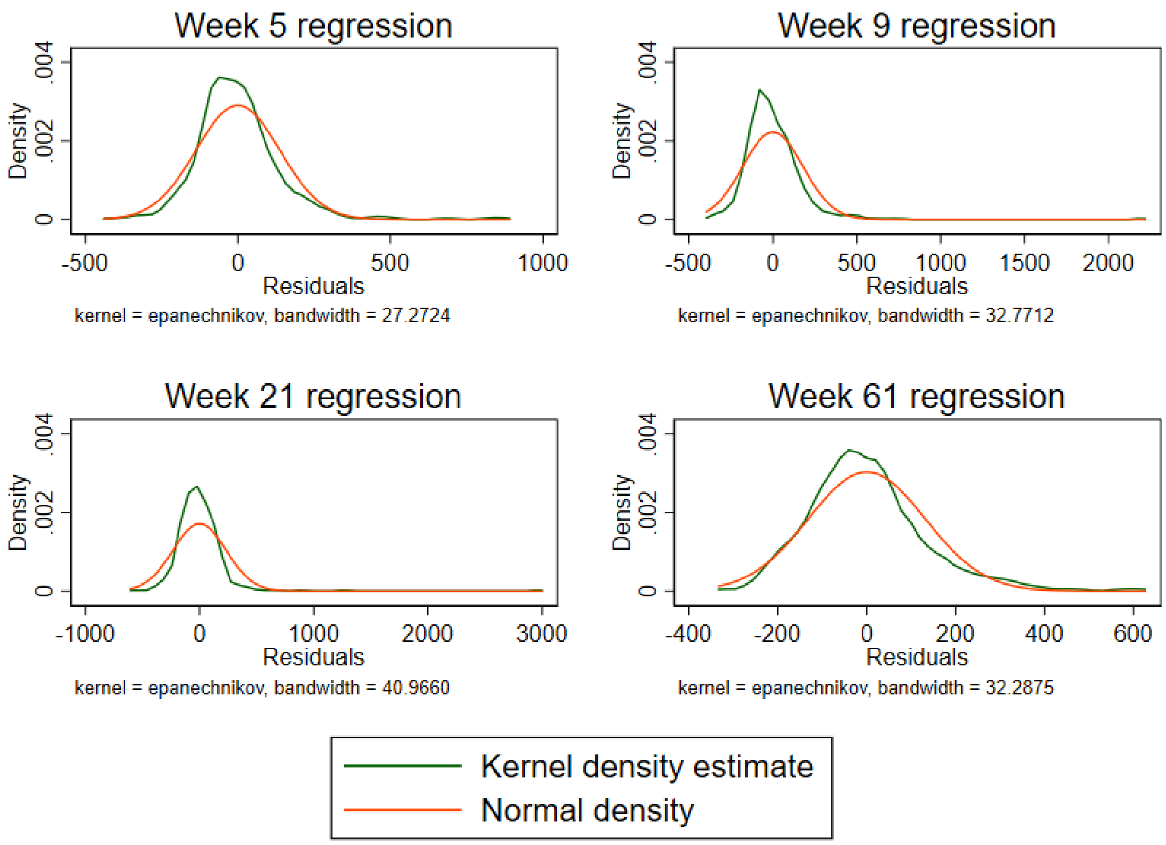

© 2025 Kókai LL et al. *JAMA Network Open*.

eAppendix. i2be App Overview and Module Scripts

Implementation of behavior change techniques via i2be modules

As noted in the main text, the behavior change techniques used in the interventions were implemented via the weekly *Get Motivated*, *Get Activated* and *Get Energized* modules that participants received in the i2be app over the 8-week intervention period. Details on the weekly content of these modules are given in the table on the next page and all behavior change techniques and their assumed working mechanism are presented in eFigure 3 in Supplement 1. All groups received a *Get Informed* module each week, providing information on the link between MVPA, HPDs, and CVD risk. Aside from some very basic tips on exercising in the Get Informed module, the modules did not provide examples of exercise sessions or programs that participants could do – it was left up to participants to decide themselves how best to accumulate MVPA minutes each week.

Modules received in a given week could be completed in any order, and could be completed separately from each other at any time during the week. The only exception was the Get Activated module, which had to be completed early in the week as it involved setting goals and making Action and Coping Plans for the week. If participants exited an incomplete module, their progress was saved and they could return to it later. Modules could only be completed during the week to which they related and could not be edited in advance or retrospectively. Participants were, however, able to read back over module content and their own responses in those modules retrospectively.

Participants in all groups received in-app virtual points, called *i2be points*, for each module they completed. Each time a participant accumulated an additional 100 i2be points they progressed to the next *Bee Level*, giving them a psychological reward. i2be points could also lead to tangible rewards. If a participant earned all available i2be points in a given week, they were entered into a weekly raffle for a sports store voucher (€25-30). If a participant accumulated 300 i2be points, they earned the right to keep the Fitbit device after the study has ended. Hence, i2be points were used mainly to incentivize in-app module completion.

There was one way in which i2be points were used to incentivize MVPA. As part of the Get Activated activity module, each week Action group participants could deposit 40 of their accumulated i2be points to make a commitment to their weekly MVPA goal. The points were refunded to them if they reached their MVPA goal – otherwise they lost the points and consequently the possibility of entering that week's raffle. This feature of Action is somewhat akin to a self-funded deposit contract that has previously been shown effective in similar experiments.[1]

---

[1] Giné, X., D. Karlan, and J. Zinman, *Put your money where your butt is: a commitment contract for smoking cessation*. American Economic Journal: Applied Economics, 2010. **2**(4): p. 213-35.
Royer, H., M. Stehr, and J. Sydnor, *Incentives, commitments, and habit formation in exercise: evidence from a field experiment with workers at a fortune-500 company.* American Economic Journal: Applied Economics, 2015. **7**(3): p. 51-84.

© 2025 Kókai LL et al. *JAMA Network Open*.

Table A: Weekly overview of behavior change techniques by i2be module.

**Behavior change technique**	**Week 1**	**Week 2**	**Week 3**	**Week 4**	**Week 5**	**Week 6**	**Week 7**	**Week 8**
Get Motivated module (received by Motivation and Action groups)								
Motivational Interviewing-Based Counselling	Identifying past successes	Running head start; Normalizing	Values exploration	Importance ruler; Confidence ruler	Hypothetical thinking; Goal attainment scaling	Query extremes; Looking forward	Identifying strengths	Recap of weeks 1-7
Get Activated (received by Action group)								
Action Planning	Action planning	Action planning	Action planning	Action planning	Action planning	Action planning	Action planning	Action planning
Coping Planning					Coping planning	Coping planning	Coping planning	Coping planning
Commitment	Commitment with i2be points; Self-designed commitment	Commitment with i2be points; Self-designed commitment	Commitment with i2be points; Self-designed commitment	Commitment with i2be points; Self-designed commitment	Commitment with i2be points	Commitment with i2be points	Commitment with i2be points	Commitment with i2be points
Get Energized (received by Action group)								
Mindfulness-Based Stress Reduction	Introduction to mindfulness	Loving kindness	Sensations	Gratitude	Humor therapy	Breath awareness	Validation	Body Scan
Positive Psychology	Three Good Things	Kindness	Three Beautiful Things	Gratitude	Three Amusing Things	Savoring	Validation	Power Posing

Notes: This table was previously published as Table 1 in the protocol for this study (Kókai et al., 2022).

© 2025 Kókai LL et al. *JAMA Network Open*.

Other i2be app features

The i2be app flow is depicted in Figure A on the next page. The user flow when logging into the app for the first time is shown in the very left-hand column. Once logged into the app, the participant was brought to the *Home* tab, which showed the modules they had to complete in the current week. Figure B below shows examples of screens from the i2be app – the top-right screen is an example of the Home tab.

In weeks 0, 9, 21 and 61 participants had an outcome measures module to complete. In weeks 0-10, 21, 22, 61 and 62 they had to complete a module reminding them to sync the Fitbit app with their Fitbit device, which was necessary for us to collect their MVPA data. In weeks 1-8 they had the activity modules relevant to their condition to complete (Get Informed, Get Motivated, Get Activated, and Get Energized). The user interface of the app utilized animated images of women from a diversity of ages and ethnic backgrounds. Female voice-overs were used in the introduction video and the audio clips.

There were also a number of other tabs which all participants could navigate to from the Home tab – *Notifications*, *My Health*, and *My Progress*. In the *Notifications* tab, participants could see notifications received informing them at the beginning of the week that the current week's modules were available and reminders later in the week to complete those modules. For participants in the Action group, they also received reminder notifications of their weekly action plans. These notifications were also received as push notifications directly to the home screen of the participant's phone.

In the *My Health* tab (bottom-middle of Figure B), participants could see a graph of their Fitbit-measured weekly minutes of MVPA and daily resting heart rate since the start of the baseline week, and also see their self-reported BMI and waist-hip ratio. In the *My Progress* tab (bottom-right of Figure B) all participants could see their i2be points and participation rewards progress and Action participants could see their progress towards their weekly commitment.

© 2025 Kókai LL et al. *JAMA Network Open*.

Figure A: i2be app flow

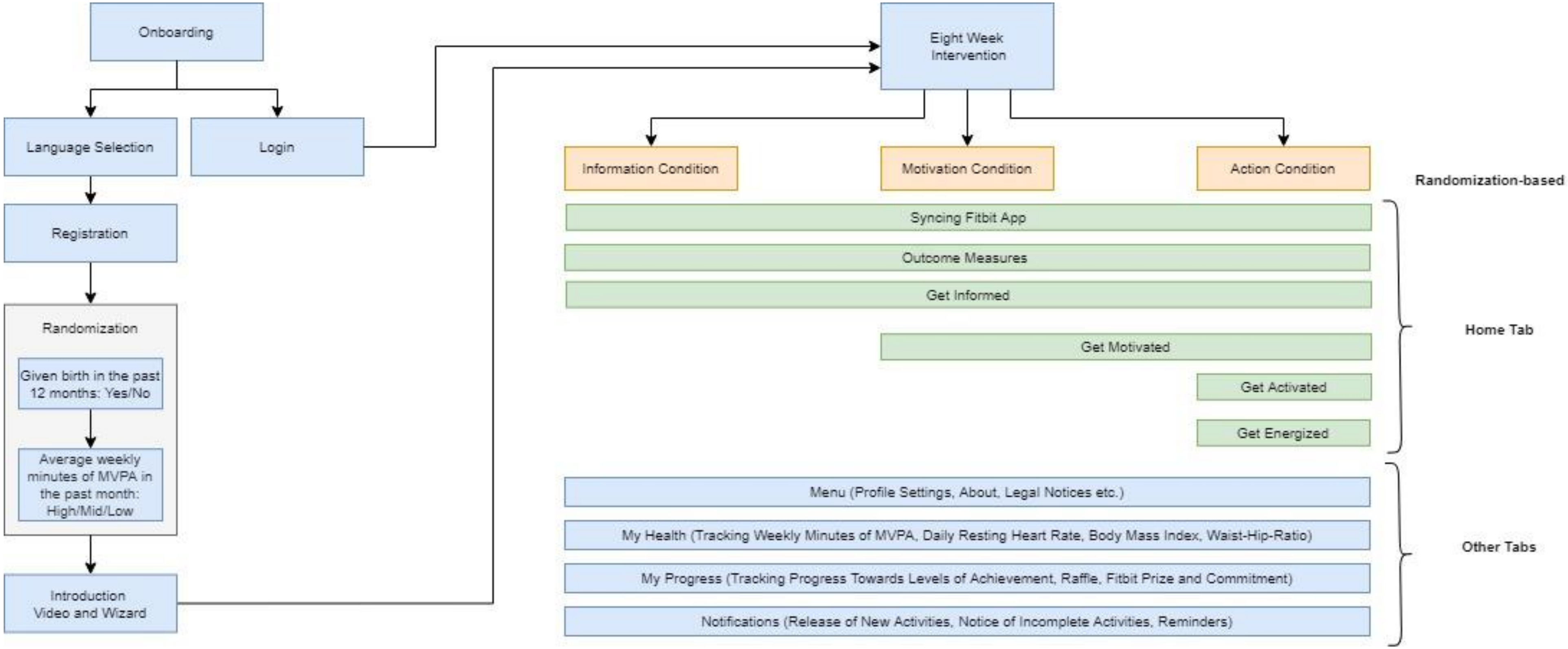

Notes: This figure was previously published as Figure 2 in the protocol for this study (Kókai et al., 2022).

© 2025 Kókai LL et al. *JAMA Network Open*.

Figure B: Examples of screens from the i2be app

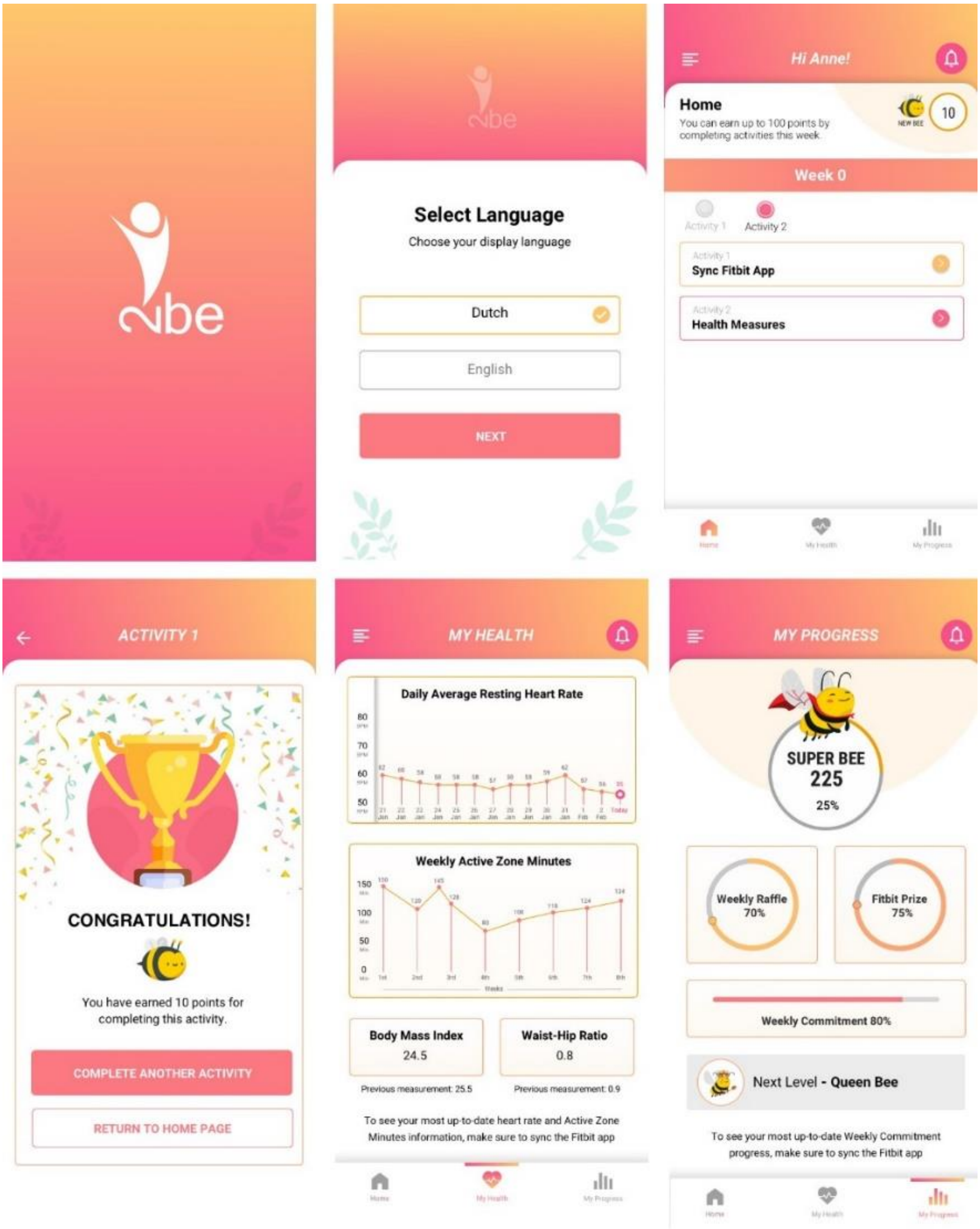

© - Avegen Limited. This figure was previously published as Figure 3 in the protocol for this study (Kókai et al., 2022).

Module “scripts”

This section provides the “script” of the intervention modules that delivered the behavior change techniques to participants via the i2be app. It does not recreate the design of these app modules.

Get Informed module content

Week 1

Relationship between high blood pressure conditions during pregnancy, risk of cardiovascular events, and physical activity
Welcome to your first Get Informed activity! Each week, we will present you with information that will allow you to make the most of the eight-week i2be program. You can always access and re-read the Get Informed activities of past weeks.
Let’s get started!

Some women have high blood pressure conditions during pregnancy, also called hypertensive disorders of pregnancy.

What are examples of high blood pressure conditions during pregnancy?

- Chronic hypertension

Chronic hypertension means having high blood pressure before you get pregnant or before 20 weeks of pregnancy. Women who have chronic hypertension can also get a condition called preeclampsia in the second or third trimester of pregnancy.

- Gestational hypertension

This condition happens when a woman has high blood pressure only during pregnancy and does not have protein in the urine or other heart or kidney problems. It is typically diagnosed after 20 weeks of pregnancy or close to delivery. Gestational hypertension usually goes away after you give birth. However, some women with gestational hypertension have a higher risk of developing chronic hypertension in the future.

- Preeclampsia/eclampsia

Preeclampsia happens when a woman who previously had normal blood pressure suddenly develops high blood pressure and protein in her urine or other problems after 20 weeks of pregnancy.
Some women with preeclampsia can develop seizures. This is called eclampsia, which is a medical emergency.

- HELLP syndrome

© 2025 Kókai LL et al. *JAMA Network Open*.

HELLP syndrome is a severe variant of preeclampsia. It can be difficult to diagnose, especially when high blood pressure and protein in the urine aren't present. HELLP syndrome is a disorder of the liver and blood that can be fatal if left untreated.
High blood pressure conditions during pregnancy increase your risk of cardiovascular events (i.e. related to heart and blood vessels) later in life.

What cardiovascular events are you at risk for after high blood pressure conditions during pregnancy?

- If your blood pressure is/was too high, it put(s) extra strain on your blood vessels, heart and other organs, such as the brain, kidneys and eyes.
- Persistent high blood pressure increases your risk of a number of serious and potentially life-threatening health conditions, such as:
    - Heart disease
    - Heart attacks
    - Strokes
    - Heart failure
    - Peripheral arterial disease
    - Aortic aneurysms
    - Kidney disease
    - Vascular dementia

Luckily, a healthy lifestyle can lower the risk of cardiovascular events in later life. Physical activity is one of the most important ways you can lower your risk of cardiovascular events after having had a high blood pressure condition during pregnancy.

How can physical activity lower risk factors, and thereby lower the risk of cardiovascular events in later life?

- Regular physical activity makes your heart stronger. A stronger heart can pump more blood with less effort. If your heart can work less to pump, the force on your arteries decreases, lowering your blood pressure.
- Becoming more active can lower your systolic blood pressure — the top number in a blood pressure reading — by an average of 4 to 9 millimeters of mercury (mmHg).

- If your blood pressure is at a desirable level — less than 120/80 mmHg — exercise can help prevent it from rising as you age.
- Exercise can keep your arteries clear by raising ‘good’ (HDL) cholesterol and lowering ‘bad’ (LDL) cholesterol levels in the blood.
- Regular exercise also helps you maintain a healthy weight — another important way to control blood pressure.
- To keep your blood pressure low, you need to keep exercising on a regular basis. It takes about one to three months for regular exercise to have an impact on your blood pressure. The benefits last only as long as you continue to exercise.

© 2025 Kókai LL et al. *JAMA Network Open*.

Knowledge check

What are some of the health benefits through which physical activity prevents and treats high blood pressure?

Select all that apply:

- Maintaining a healthy weight
- Increasing blood sugar
- Strengthening the heart

SUBMIT

Sources

Center for Disease Control – High blood pressure during pregnancy

National Health Service United Kingdom – High blood pressure

Blood Pressure United Kingdom – High blood pressure

RETURN TO HOME SCREEN

© 2025 Kókai LL et al. *JAMA Network Open*.

Week 2

Guidelines for physical activity
Physical activity can be divided into different levels of intensity, namely sitting, light physical activity, moderate physical activity and vigorous physical activity.
Physical activities at moderate and vigorous intensity have health enhancing effects. Therefore, activities at these intensity levels are the ones recommended per international guidelines and your Fitbit device.

How can I tell a moderate activity from a vigorous one?
Vigorous activities take more effort than moderate activities. Here are just a few moderate and vigorous physical activities.

Moderate activities
You can talk while you do them, but you couldn't sing; your heart rate is somewhat high.

- Walking briskly
- Cycling briskly
- General gardening (raking, trimming shrubs)
- Sports where you catch and throw (baseball, volleyball)
- Tennis (doubles)
- Using your manual wheelchair
- Using hand cyclers - also called ergometers
- Water aerobics

Vigorous activities
You can only say a few words without stopping to catch your breath while doing them; your heart rate is high.

- Jogging or running
- Hiking uphill
- Cycling faster than 20 km/h
- Heavy gardening (digging, hoeing)
- Sports with a lot of running (basketball, hockey, soccer)
- Tennis (singles)
- Aerobic dance
- Jumping rope
- Martial arts (such as karate)
- Swimming laps

Yoga, washing dishes and tidying up are examples of light physical activity: you could still sing while performing these activities, and your heart rate does not go up very high.

International physical activity guidelines
The World Health Organization recommends that adults do at least 150 minutes of moderate-intensity physical activity, 75 minutes of vigorous intensity physical activity, or an equivalent combination of moderate- and vigorous-intensity activity throughout the week.

© 2025 Kókai LL et al. *JAMA Network Open*.

Some physical activity is (much) better than none! If you are not currently meeting these recommendations, doing some physical activity will already bring benefits to your health. You can start with small amounts of physical activity and gradually increase duration, frequency, and intensity over time.

How do the guidelines link with the Active Zone Minutes of Fitbit?

- Every minute that you spend doing moderate or vigorous activities, as recommended by the guidelines, will be registered by Fitbit as Active Zone Minutes*.
- The fat burn heart rate zone corresponds to moderate physical activity. The cardio and peak heart rate zones correspond to vigorous physical activity, and are counted double!

*Note that only Fitbit Inspire 2 (the one you got from i2be), Fitbit Charge 4, Fitbit Ionic, Fitbit Sense, and Fitbit Versa series track Active Zone Minutes – so your friend's older generation Fitbit may not have this feature.

Knowledge check
What is the minimum number of Active Zone Minutes you need to earn per week to meet the international guideline level of physical activity?
Choose the correct option:

- ○ 150
- ○ 75
- ○ 150 or 75 or an equivalent combination of both

SUBMIT

Sources
[World Health Organization - Physical Activity Guidelines]
[Fitbit - Help]

RETURN TO HOME SCREEN

© 2025 Kókai LL et al. *JAMA Network Open*.

Week 3

Active Zone Minute exercises – aerobic exercise
In the coming two weeks we will cover two types of exercises that contribute to your levels of moderate to vigorous physical activity: aerobic exercise and anaerobic exercise. This week we start with aerobic exercise.

What is aerobic exercise?
Aerobic exercise is a continuous moderate to vigorous activity where the body's large muscles move in a rhythmic manner for a sustained period of time. Examples include jogging, cycling or swimming. Your breathing and heart rate will increase during aerobic activities. Aerobic exercise helps keep your heart, lungs, and circulatory system healthy.

Benefits of aerobic exercise

- Aerobic exercise is recommended for people with, or at risk of, cardiovascular disease - such as women who have experienced a high blood pressure condition during pregnancy. That's because exercise strengthens your heart and helps it pump blood more efficiently throughout the body.
- Aerobic exercise can also help lower blood pressure, and keep your arteries clear by raising 'good' (HDL) cholesterol and lowering 'bad' (LDL) cholesterol levels in the blood.
- Regular aerobic activity helps regulate insulin levels and lower blood sugar, thereby helping to prevent or regulate type 2 diabetes.
- Regular low-impact aerobic activity, like walking or cycling at a slow pace, can help reduce chronic pain.
- The list goes on: aerobic exercise can lead to improved brain function and mood, better sleep, weight loss, stronger immune function and reduced occurrence of injury and falls.

Examples of aerobic exercise
You don't necessarily need to go to the gym to do aerobic exercise. There are many exercises you can do with little to no equipment, too. For example:

Brisk cycling

Cycle at a brisk pace (faster than you would leisurely, at a speed of about 16 to 20 km/h).

Brisk walking

Walk at a brisk pace (faster than you would walk leisurely, at a speed of about 5 to 8 km/h).

© 2025 Kókai LL et al. *JAMA Network Open*.

Knowledge check

What is an example of aerobic exercise?

Choose the correct option:

- Sit-ups
- Brisk walking
- Yoga

SUBMIT

Further resources

If you want to try other types of aerobic exercise, get inspired by the National Health Service United Kingdom.

Sources

National Health Service United Kingdom – Aerobic Exercises

Kenniscentrumsport

RETURN TO HOME SCREEN

© 2025 Kókai LL et al. *JAMA Network Open*.

Week 4

Active Zone Minute exercises - anaerobic exercise
Last week we have covered one of the two main types of activity recommended by international guidelines: aerobic exercise. In this week, we will cover the second main type of exercise, which is anaerobic exercise. This type of exercise is also called muscle-strengthening exercise.

What is anaerobic exercise?
Anaerobic exercise is quick bursts of intense activity that you can't sustain for very long, with rest periods in between. Examples include weight training and sprinting.

Benefits of anaerobic exercise

- A strong body allows you to exert force and lift weight.
- Muscle tissue is metabolically more active and burns more calories than fat tissue. This means that as your body starts to build muscle tissue, you will burn more calories, even when you are in a resting state (i.e. doing nothing).
- Muscular strength helps to develop good posture and relieve back pain.
- You'll have more stability, balance, and flexibility, making injuries and falls less likely.

Muscle-strengthening exercise examples
You don't necessarily need to go to the gym to do muscle-strengthening exercises. There are many you can do with little to no equipment, too. For example:

Bicep curls

Hold a pair of light weights (filled water bottles will do) and stand with your feet hip-width apart.
Keeping your arms by your side, slowly bend them until the weight in your hand reaches your shoulder.
Slowly lower again.
This can also be carried out while sitting.

Squats

Stand with your feet hip-width apart. Rest your hands on the back of the chair for stability if needed.
Slowly bend your knees as far as is comfortable, as if about to sit down. Don't let your knees sag inwards. Aim to get them over your big toe. Keep your back straight at all times.
Gently come up to standing, squeezing (clenching) your buttocks as you do so.

Knowledge check
What is an example of anaerobic exercise?

© 2025 Kókai LL et al. *JAMA Network Open*.

Choose the correct option:

- Bicep curls
- Cycling
- Washing dishes

SUBMIT

Further resources

If you want to try other types of anaerobic exercise, get inspired by the National Health Service United Kingdom.

Sources

National Health Service United Kingdom – Strength Exercises

Kenniscentrumsport

RETURN TO HOME SCREEN

© 2025 Kókai LL et al. *JAMA Network Open*.

Week 5

Warming up and cooling down
When preparing for any kind of exercise, it's important to ease your muscles into exercise mode. Try to spend 5 to 10 minutes warming up.
It's equally important to ease yourself out of strenuous activity at the end of your workout. Dedicate 5 to 10 minutes to cooling down.
Please note that as warming up and cooling down may not get your heart rate up, they most likely will not count towards your Active Zone Minutes.

What are the benefits of warming up before a workout?

- Warming up increases your flexibility, making it easier to move and exercise correctly.
- Warming up your muscles can help them relax which, in turn, can lead to less injury.
- Warming up increases your blood flow, helping your muscles get the nourishment they need before launching into more intense work.
- Warmed up muscles can help you work out more effectively.
- Warming up gives you greater range of motion, which can help you move your joints more fully.
- Muscles that are warm and relaxed may help you move more easily and with less pain or stiffness.

A dynamic warmup is a common way to warm up. A dynamic warmup focuses on actions similar to the movements you'll do while you work out.

Example of a dynamic warmup
March on the spot

Start off marching on the spot and then march forwards and backwards. Pump your arms up and down in rhythm with your steps, keeping the elbows bent and the fists soft.

What are the benefits of cooling down after a workout?

- Cooldown exercises start the recovery process, increase flexibility, and promote relaxation.
- A gradual cooldown keeps your blood circulating and prevents it from pooling in your veins, which can cause you to feel lightheaded or dizzy.
- Cooling down allows your body temperature, blood pressure, and heart rate to return to their normal levels.
- Stretching your muscles while they're still warm can help to reduce lactic acid buildup, reducing your chance of muscle cramps and stiffness.
- Stretches elongate the connective tissue around your joints, increase mobility, and improve range of motion.

© 2025 Kókai LL et al. *JAMA Network Open*.

Static stretching is a common way to cool down. Static stretching consists of stretches that are held for a period of time to help lengthen and loosen your muscles and connective tissue.

Hamstring stretch

Lie on your back and raise your right leg.
Hold your right leg with both hands, below your knee.
Keeping your left leg bent with your foot on the floor, pull your right leg towards you keeping it straight.
Repeat with the opposite leg.

Knowledge check
What is an example of static stretch?
Choose the correct option:

- Hamstring stretch
- March on the spot
- Warm up

SUBMIT

Further resources
If you want to try other types of warming up exercises, get inspired by the National Health Service United Kingdom.
If you want to try other types of cooling down exercises, get inspired by the National Health Service United Kingdom.

Sources
National Health Service United Kingdom – Warming up
National Health Service United Kingdom – Cooling down
Allesoversport

RETURN TO HOME SCREEN

© 2025 Kókai LL et al. *JAMA Network Open*.

Week 6

Listen to your body

One of the primary causes of injury is not listening to your body during physical activity. Not listening to your body is popularized by some widespread, damaging myths about exercise, such as *"no pain, no gain."* You can significantly lower your risk of injury by following these simple tips:

Build up gradually

- Always warm up before and cool down after your workout.
- Start slowly, and gradually build up the intensity and duration of your workouts.
- Use caution if you're new to exercise or have any injuries or medical concerns. For instance, consult your health care provider before resuming high intensity exercise after a cesarean (C-section).
- If you want to lose weight, aim to lose no more than 1 kilogram per week. This is especially important if you are breastfeeding: rapid weight loss may alter the volume or composition of your breast milk.

Take enough rest

- While breathing more heavily is a normal part of exercise, avoid being short of breath, as this could cause dangerous spikes in blood pressure.
- Stop anytime you need a break, start to feel pain, dizziness or nausea.
- Allow for one day of recovery between high intensity workouts, especially if they are working the same muscle group.

Use proper form

- Always use proper form (such as keep you back straight). Check information by reliable sources (such as the resources we have presented in previous weeks) before attempting a new move yourself.
- Use steady, controlled movements, especially if you're doing heavy lifting.
- Find a training partner. You can help each other use proper form.
- If it's an option, ask your health care provider to refer you to an expert to provide opinion on your form.

Knowledge check

Which of the following is a myth?

Choose the correct option:

- ○ Don't lose more than 1 kilogram per week if you are breastfeeding
- ○ Always cool down after a workout
- ○ No pain, no gain

SUBMIT

Sources

[National Health Service United Kingdom - Common exercise mistakes]

© 2025 Kókai LL et al. *JAMA Network Open*.

Summary of International Guidelines for Physical Activity Following Pregnancy
Randomized trial of the short-term effects of dieting compared with dieting plus aerobic exercise on lactation performance

RETURN TO HOME SCREEN

© 2025 Kókai LL et al. *JAMA Network Open*.

Week 7

A healthy lifestyle

Sufficient physical activity is just one component of a healthy lifestyle. Everyone can improve their physical health status by establishing and maintaining a healthy lifestyle.

A healthy lifestyle is especially important for women who have experienced high blood pressure during pregnancy, as they are already at higher risk for cardiovascular events.

The main components of a healthy lifestyle are to:

- *Not have overweight or obesity:* An indicator of overweight or obesity is a high body mass index (BMI>25, and BMI>30, respectively). Losing even a small amount of weight if you're overweight or obese can help reduce your blood pressure. Cutting your calorie intake from food and increasing your calorie burn through physical activity are both important in losing weight.
- *Eat a healthy diet:* Eat more vegetables and fruits; eat foods high in fiber and low in saturated and trans-fats; and reduce your salt and sugar intake.

- *Do not smoke:* Each cigarette you smoke increases your blood pressure for many minutes after you finish, and increases your risk for cardiovascular disease by damaging the blood vessels.
- *Exercise at least 150 minutes each week:* Physical activity levels that make your breathing heavier and your heart rate higher count towards these minutes, i.e. Active Zone Minutes. Regularity is key! But you know this already.

The behaviors described above are all called 'ideal health behaviors'. The more ideal health behaviors you do, the more chance that your risk for cardiovascular events will go down. Start with baby steps – small successes every day add up to a big impact!

Knowledge check

Which of the following are 'ideal health behaviors'?

Select all that apply:

- ○ Having a BMI of 30
- ○ Smoking only 5 cigarettes a day
- ○ Reducing salt intake

SUBMIT

Sources

[Heart.org – Life's Simple 7]

RETURN TO HOME SCREEN

© 2025 Kókai LL et al. *JAMA Network Open*.

Week 8

Well-being

Well-being is the perception that your life is going well. There is general agreement that at minimum, well-being includes:

- *Physical well-being,* which relates to vigor and vitality, feeling healthy and energetic, often as a result of leading a healthy lifestyle.
- *Mental well-being,* which includes being satisfied with one's life; balancing positive and negative emotions; accepting one's self; finding purpose and meaning in one's life; seeking personal growth, autonomy, and competence; believing one's life and circumstances are under one's control; and generally experiencing optimism.
- *Social well-being,* which involves providing and receiving quality support from family, friends, and others.

When people think of the benefits of physical activity, they generally think of improvements in physical well-being. However, physical activity can improve your mental and social well-being too! For instance, physical activity can reduce stress and help you stay connected with others. Of course, there are other ways to reduce stress and stay connected as well.

Reduce stress

- *Identify your sources of stress*: What events or situations trigger stressful feelings? Are they related to your family, health, finances, work, relationships, or something else?
- *Recognize your own stress signals:* People experience stress in different ways. You may have a hard time concentrating or making decisions, feel angry, irritable or out of control, or experience headaches, muscle tension, or a lack of energy.

- *Eliminate unhealthy ways of managing stress:* Try to eliminate unhealthy ways of managing stress one at a time, such as smoking or over/under eating. Don't take on too much at once, be patient with yourself.
- *Find healthy ways to manage stress:* Consider healthy, stress-reducing activities such as meditation, physical activity, or talking things out with friends or family.
- *Take care of yourself:* Lead a healthy lifestyle as much as you can, including being physically active. In addition, no matter how hectic life gets, make time for yourself — even if it's just to read a good book or listen to your favorite music.

- *Reach out for support:* Accepting help from supportive friends and family can improve your ability to manage stress. If you feel overwhelmed by stress, you may want to ask your health care provider to refer you to a qualified counsellor.

Stay connected to others

- *Cast a wide net:* You may not have someone you can confide in about everything — and that's okay. Look to different relationships for different kinds of support.

© 2025 Kókai LL et al. *JAMA Network Open*.

- *Be proactive:* Make time for friends and family. Reach out to lend a hand or just say hello. If you're there for others, they're more likely to be there for you.
- *Take advantage of technology:* It's nice to meet face-to-face, but it isn't always possible. Luckily, technology makes it easier than ever before to stay connected with loved ones, or to join an online workout session.

- *Follow your interests:* You're more likely to connect with people who like the things you like. Join a sports club, sign up for an (online) class, or take on a volunteer position that will allow you to meet others who share your interests.
- *Seek out support:* If you're dealing with a specific stressful situation — such as having experienced a high blood pressure condition during pregnancy — you may not find the support you need from your current network. Consider joining a support group to meet others who are dealing with similar challenges.

Knowledge check
What are some ways to improve your well-being?
Select all that apply:
- ○ Being physically active
- ○ Reducing stress
- ○ Staying connected

SUBMIT

We hope that you have enjoyed the Get Informed activities of the past eight weeks! You will continue to have access to these activities until the final follow-up questionnaire of i2be 12 months from now, although the knowledge checks will not open up again. However, we encourage you to:

- Read through the activities again
- Visit all the links leading to additional resources online
- Discuss Get Informed with a friend or family member!

Further resources
Heart.org – High blood pressure in women
HELLP Syndrome Patient Organization (Netherlands)
Preeclampsia, Eclampsia & HELLP Syndrome Facebook Group (Netherlands and Belgium)
Preeclampsia Foundation's Community Forum (Worldwide)
Preeclampsia, Eclampsia & HELLP Syndrome Facebook Group (Worldwide)

Sources
Center for Disease Control – Well-being
American Psychological Association – Social support
American Psychological Association – Stress reduction

RETURN TO HOME SCREEN

© 2025 Kókai LL et al. *JAMA Network Open*.

Get Motivated app module content

Week 1

Successes
Welcome to your first Get Motivated activity! We are so excited that you have decided to commit to i2be for the coming eight weeks. Discovering your motivations will be an insightful journey for sure! You can always access the Get Motivated activities of past weeks if you would like to re-read your answers.

Let's get started!

A good starting point for mobilizing your resources is to think of successes that you have experienced in the past. This may seem strange, but reflecting on past events when you felt like you have succeeded can feed into future successes.

Think of a time in the past when you have made positive changes to your physical activity on your own initiative – a time when you have decided to take charge. Describe what happened.
Write here...

What obstacles (whether in yourself, or in your environment) did you have to overcome to make the change?
Write here...

How did you overcome the obstacle (whether in yourself, or in your environment) to make the change?
Write here...

When thinking about how you overcame the obstacle, can you think of some personal strengths of yours that were especially important to your success?
Write here...

Did you use these strengths of yours again to achieve something else (whether related to physical activity, or not) that you wanted?
Write here...

Hopefully this activity gave you some insight into how you have already experienced successes with regard to physical activity in the past. Furthermore, it is important to realize that your success was likely dependent on some of your most important personal strengths.

Tip

© 2025 Kókai LL et al. *JAMA Network Open*.

It is important to know your personal strengths, so that you can apply them again and again in various contexts.

Great job! You have completed week one of Get Motivated with flying colors.

RETURN TO HOME SCREEN

© 2025 Kókai LL et al. *JAMA Network Open*.

Week 2

The way things are
Welcome back! This week we would like to explore with you the *good things* about the way things currently are with regard to your physical activity.

Think of three things that you are satisfied with when thinking about your current physical activity. For example: I walk regularly with my dog. I stretch multiple times a day. I work in the garden as often as I can.

First thing I am satisfied with
Write here...

Second thing I am satisfied with
Write here...

Third thing I am satisfied with
Write here...

Next, we would like to explore with you the *not so good things* about the way things currently are with regard to your physical activity.

Think of three things that you are not satisfied with when thinking about your current physical activity. For example: I have a yoga mat at home, but it just gathers dust. I am less active than I was five years ago. I am out of breath when I join my friends for a walk.

First thing I am not satisfied with
Write here...

Second thing I am not satisfied with
Write here...

Third thing I am not satisfied with
Write here...

Thank you for your answers! Here is a summary of your contradictory feelings about your current physical activity.
#Cue recap response 2; cue recap response 5
#Cue recap response 3; cue recap response 6
#Cue recap response 4; cue recap response 7

Both feelings are equally valid! While having contradictory feelings may feel uncomfortable, it is only natural to experience them.

How does it make you feel to see your contradictory feelings about your current physical activity next to each other?

© 2025 Kókai LL et al. *JAMA Network Open*.

Write here…

Do the things that you are satisfied with motivate you to engage in (more) physical activity?
☐ Yes ☐ No
Because…
Write here…

Do the things that you are not satisfied with motivate you to engage in (more) physical activity?
☐ Yes ☐ No
Because…
Write here…

Name two things that motivate you most to work on your physical activity in the coming eight weeks (and beyond).

First motivator
Write here…

Second motivator
Write here…

Thank you for your responses! We hope that you have enjoyed this activity – perhaps you had some novel insights about your satisfaction with your current physical activity, as well as about your biggest personal motivators and demotivators.

You revealed to us last week that you have several personal strengths that have helped you achieve your physical activity goals in the past:
#Cue recap response 1

Tip
You might consider using your personal strengths to change the things you are not so happy with when thinking of your current physical activity.

Stay positive, you are on track for i2be success!

RETURN TO HOME SCREEN

© 2025 Kókai LL et al. *JAMA Network Open*.

Week 3

Values

Values are the things that we believe are worthy in life, and influence how we spend our time and energy. While some of your values may be obvious to you, others may be so deeply seated that you are not aware of them day-to-day. Knowing your values can help you live your life in a worthy way.

Let's determine your values. Select at least ten values that resonate with you. Don't take too much time to make your selection. If there are values that are important to you that are not on the list, please write them down as well.

Accountability Achievement Advancement Adventure Ambition Boldness Calmness Caring Cheerfulness Cleverness Community Commitment Compassion Cooperation Consistency Creativity Credibility Curiosity Decisiveness Dependability Empathy Enthusiasm Fairness Family Friendships Flexibility Freedom Generosity Grace Happiness Health Honesty Humility Humor Independence Inspiration Intelligence Intuition Joy Kindness Knowledge Loyalty Optimism Open-Mindedness Originality Passion Safety Spirituality Stability Playfulness Proactivity Professionalism Reliability Resilience Resourcefulness Responsibility Safety Self-Control Selflessness Success Thankfulness Thoughtfulness Traditionalism Trustworthiness Wisdom

Additional value

Please see your values below. From all your values, please select your five most important values.

Most important value
Add here...

Second important value
Add here...

Third important value
Add here...

Fourth important value
Add here...

Fifth important value
Add here...

Thank you for your input!

Do you feel like you live your life according to your values?
Write here...

© 2025 Kókai LL et al. *JAMA Network Open*.

What changes could you make to your life in general to live an even more valuable life?
Write here...

How do your physical activity practices fit in with your values?
Write here...

What changes could you make to your physical activity to live an even more valuable life?
Write here...

Tip
If physical activity is part of a worthy life for you, then you might consider moving a little more the coming week than you did in the last week. Every little step is valuable and will bring you closer to your goals.

Our values make us into who we are, and living our lives in accordance with our values makes a worthy life.

RETURN TO HOME SCREEN

© 2025 Kókai LL et al. *JAMA Network Open*.

Week 4

Importance and confidence
This week we will first explore your motivation in terms of how *important* it is for you to make a change with regard to your physical activity. To do so we will use the *Importance Ruler*.

On a scale of 0 to 10, where 0 means *Not at all important,* and 10 means *The most important thing for me right now*, how important would you say it is for you to reach your desired level of physical activity within the next year?

Importance Ruler
0=Not at all important, 1, 2, 3, 4, 5, 6, 7, 8, 9, 10=The most important thing for me right now

---

Next, we will explore your motivation in terms of how *confident* you are to make a change with regard to your physical activity. To do so we will use the *Confidence Ruler.*

On a scale of 0 to 10, where 0 means *Not at all confident*, and 10 means *The most confident I can be*, how would you rate your confidence that you can reach your desired level of physical activity within the next year?

Confidence Ruler
0= Not at all confident, 1, 2, 3, 4, 5, 6, 7, 8, 9, 10=The most confident I can be

---

Thank you for your input! Here is how important you think it is for you to reach your desired level of physical activity in the next year:
#Cue recap response 15

Before thinking about this on a scale, would you have expected it to be more/less important to you to reach your desired level of physical activity in the next year?
☐ More ☐ Less ☐ I expected this
Because...
Write here...

Two weeks ago you told us that this would motivate you to work on your physical activity in the coming eight weeks (and beyond):
#Cue recap response 8

Does seeing your personal motivator increase your rating on the *Importance Ruler*?
☐ Yes ☐ No
#If Yes:
Importance Ruler
0=Not at all important, 1, 2, 3, 4, 5, 6, 7, 8, 9, 10=The most important thing for me right now
Because...
Write here...

© 2025 Kókai LL et al. *JAMA Network Open*.

What (else) specifically could increase your rating on the *Importance Ruler*?
Write here…

And here is how confident you are that you can reach your ideal level of physical activity in the next year:
#Cue recap response 16

Before thinking about this on a scale, would you have expected to be more/less confident that you can reach your ideal level of physical activity in the next year?
☐ More ☐ Less ☐ I expected this
Because…
Write here…

Two weeks ago you told us that this would motivate you to work on your physical activity in the coming eight weeks (and beyond):
#Cue recap response 9

Does seeing your personal motivator increase your rating on the *Confidence Ruler*?
☐ Yes ☐ No
#If Yes:
Confidence Ruler
By 1/2/3/4/5/6/7/8/9/10 scale points
0= Not at all confident, 1, 2, 3, 4, 5, 6, 7, 8, 9, 10=The most confident I can be
Because…
Write here…

What (else) specifically could increase your rating on the *Confidence Ruler*?
Write here…

Tip
The importance you attach to your physical activity, and your confidence in reaching your physical activity goals is not static: they can be increased through experience. As you progress towards your goals, they will grow with you!

You are already rocking the i2be program! Keep going!

RETURN TO HOME SCREEN

© 2025 Kókai LL et al. *JAMA Network Open*.

Week 5

Goals
Meaningful goals, in other words goals that are in line with your values, can give direction to your life. You have identified your most important values to be:
#Cue recap response 10, 11, 12, 13, 14

Today, we will first start with the big picture of what you would like to achieve with regard to physical activity.

What is your one-month goal with regard to physical activity?
Write here...

What is your one-year goal with regard to physical activity?
Write here...

What is your five-year goal with regard to physical activity?
Write here...

You have set some great goals – well done. However, it is common to set the bar too high, especially when looking at the big picture of our lives. Having difficulties with change is actually very common. Not succeeding immediately does not mean that you are failing to achieve your goals; rather, it means that you are still learning how to set the right goals for yourself.

Try to predict the future as realistically as you can. Using a -3 to +3 scale, where 0 is The way things were at the outset of the i2be program, -3 is A big step back from the way things were, and +3 is A big step ahead from the way things were.

What do you think you will have achieved in one month from now with regard to your one-month goal?
-3= A big step back from the way things were, -2, -1, 0=The way things were at the outset of the i2be program, 1, 2, 3=A big step ahead from the way things were

What do you think you will have achieved in one year from now with regard to your one-year goal?
-3= A big step back from the way things were, -2, -1, 0=The way things were at the outset of the i2be program, 1, 2, 3=A big step ahead from the way things were

What do you think you will have achieved in five years from now with regard to your five-year goal?
-3= A big step back from the way things were, -2, -1, 0=The way things were at the outset of the i2be program, 1, 2, 3=A big step ahead from the way things were

---

© 2025 Kókai LL et al. *JAMA Network Open*.

If you have honestly given yourself ratings of 1 or higher, pat yourself on the back: you have set realistic goals for the future. In this case, please *reconfirm* your previously stated five-year goal for physical activity by writing it down *again*.

If you have given yourself ratings of 0 or lower, praise yourself for excellent self-reflection abilities – it is difficult to realize that you have set the bar too high! Make sure to scale back your goals. Please *adjust* your previously stated five-year goal for physical activity, and write down this *revised* goal.

My (revised) five-year physical activity goal is...
Write here...

Tip
Set goals that are right for you: just at the edge of discomfort. Be kind to yourself, and acknowledge that you are putting in the effort to live your best life!

You have a vision of where you want to be in life. Keep direction every day!

RETURN TO HOME SCREEN

© 2025 Kókai LL et al. *JAMA Network Open*.

Week 6

Extreme consequences
This week we will imagine the best possible consequences of changing the way things are with regard to your physical activity - as well as the worst possible consequences of *not* changing the way things are with regard to your physical activity! Don't be afraid of thinking of extreme consequences - the point of this activity is to stretch your imagination.

Also, we will recap and give you some tips on how to use all the skills that you have learned in the past five weeks.

To get you started, we would like to remind you of your five-year goal with regard to physical activity:
#Cue recap response 19

Imagine that you were *completely successful* in making the changes necessary to achieve your five-year goal.

How would things be (much) better in your life?
Write here...

What is it that you could be doing more (or doing again) that you like?
Write here...

How would you feel then?
Write here...

Now, imagine that you were *completely unsuccessful* in making the changes necessary to achieve your five-year goal.

How would things be (much) worse in your life?
Write here...

What is it that you could not do much (or at all) that you like?
Write here...

How would you feel then?
Write here...

Tip
When you are low on motivation, think about the best and the worst possible consequences of (not) achieving your five-year physical activity goals.

© 2025 Kókai LL et al. *JAMA Network Open*.

Thinking about the possible negative consequences of your physical inactivity can feel uncomfortable. However, remember that all growth is uncomfortable, and that good things happen when you dare to grow!

You have discovered so much about yourself in the past six weeks! We would like to give you some fun tips on how to use your discoveries to fuel your motivation.

Are you ready?

You have used some personal strengths in the past to successfully overcome an obstacle to physical activity:
#Cue recap response 1

Use this strength again and again! One day you won't even have to think of it, and you will overcome obstacles like it is wired into you.

Will you use your strength to overcome an obstacle with regard to physical activity today?
☐ Yes ☐ No

You told us that you find these two things especially motivating in your goal striving for physical activity in the these eight weeks (and beyond):
#Cue recap response 8
#Cue recap response 9

It is so important to learn how to motivate yourself – it doesn't have to be one size fits all. You can write your biggest motivators on a sticky note, and stick it on the fridge!

Will you put up a sticky note of your biggest motivators on your fridge today?
☐ Yes ☐ No

You have recently identified your most important values:
#Cue recap response 10, 11, 12, 13, 14

You will feel fulfilled if you strive every day to live your life according to your values. And leading a healthy lifestyle with regard to physical activity fits right in with that! To remind yourself, you could write your most important values on a sticky note, and stick it on the inside of your front door.

Will you put up a sticky note of your most important values on the inside of your front door today?
☐ Yes ☐ No

Tip
The decision to lead a healthy lifestyle is in your hands every single moment of every single day.

© 2025 Kókai LL et al. *JAMA Network Open*.

We hope that you are motivated to realize the best possible consequences of being physically active! Be confident, you can do it!

RETURN TO HOME SCREEN

© 2025 Kókai LL et al. *JAMA Network Open*.

Week 7

Strengths
Those who know their strengths tend to use them more frequently, and therefore succeed in many areas of life. While some of your strengths may be obvious to you, others might feel so natural to you that you don't even realize what a unique strongpoint you have.

We know some of your strengths already:
#Cue recap response 1

Surely, there are many more!

Determine your (additional) strengths. Read through the list below, and select at least ten strengths that you possess. Don't take too much time to make your selection. If there are strengths that you possess that are not on the list, make sure to write them down as well.

Action-oriented Adventurous Ambitious Analytical Artistic Assertive Athletic Authentic Caring Clever Compassionate Charming Confident Courageous Creative Curious Decisive Detail-oriented Determined Disciplined Empathetic Energetic Enthusiastic Fair Focused Friendly Generous Grateful Helpful Honest Hopeful Humble Humorous Independent Industrious Inquisitive Inspirational Intelligent Kind Knowledgeable Modest Moral Motivated Optimistic Open-minded Original Organized Patient Peaceful Persuasive Persistent Resourceful Respectful Responsible Self-assured Self-controlled Spiritual Spontaneous Social Straightforward Strategic Team-oriented Thoughtful Trustworthy Welcoming Wise

Additional strength

Please see your strengths below. Select your five most important strengths.

Most important strength
Add here...

Second important strength
Add here...

Third important strength
Add here...

Fourth important strength
Add here...

Fifth important strength
Add here...

Thank you for your input!

© 2025 Kókai LL et al. *JAMA Network Open*.

How do you use your personal strengths in daily life?
Write here...

In what additional ways could you use your strengths in daily life?
Write here...

How do you use your personal strengths to be (more) physically active?
Write here...

In what additional ways could you use your strengths to be (more) physically active?
Write here...

Tip
Keep applying your strengths to work on your physical activity.

You have enough resources in stock!

RETURN TO HOME SCREEN

© 2025 Kókai LL et al. *JAMA Network Open*.

Week 8

Future directions
This is the final activity of the eight-week i2be program – we hope that you have found it enjoyable and useful, and that you have learned some life-long lessons about your deepest motivations! Now it's time to look to the future, with your discoveries and techniques in your back-pocket.

We would like to give you some fun tips on how to use your discoveries to fuel your motivation.

Previously you have told us that this reason could make the importance of reaching your physical activity goals even higher:
#Cue recap response 17

Whenever you are low on motivation, it would be nice if someone could remind you of this special motivator. Tell your loved ones about it, so that they can give you a boost when you need it most!

Will you tell a loved one about this important reason for physical activity for you today?
☐ Yes ☐ No

You also told us that this could make you even more confident in reaching your physical activity goals:
#Cue recap response 18

Confidence is so important in helping you stick to your physical activity goals. You could remind yourself monthly about what gives you assurance!

Will you set a calendar event in your phone today, repeating monthly, that will remind you of this thing that can make you more confident about reaching your physical activity goals?
☐ Yes ☐ No

Remember your five-year goal with regard to physical activity?
#Cue recap response 19

Visualize this amazing, strong and healthy person that you could be five years from now. Positively visualizing your future can act as a strong motivator for your goal striving.

Write about ten sentences about this amazing future self that you are visualizing. Let optimism take over – there is no reason to think that you cannot live your best life!
Write here...

© 2025 Kókai LL et al. *JAMA Network Open*.

The last activity of today is about your strengths. You have identified your most important strengths to be:
#Cue recap response 20, 21, 22, 23, 24

You have already brainstormed novel ways to apply your strengths to physical activity:
#Cue recap response 25

Wow, it seems like you have amazing resources to rely on! To remember them, you could write your most important strengths on a sticky note and stick it on the mirror in your bathroom.

Will you put up a sticky note of your biggest strengths on your bathroom mirror today?
☐ Yes ☐ No

Tip
Return to Get Motivated at any time during the next 12 months to re-read or re-do the activities of the past eight weeks!

Even if practice doesn't make perfect, it will still make pretty great.

We hope that you have enjoyed the Get Motivated activities of the past eight weeks! You will continue to have access to these activities until the final follow-up questionnaire of i2be 12 months from now, although the response boxes will not open up again. However, we encourage you to:

- Scroll through all activities again and answer on a sheet of paper
- Discuss your answers with a friend or family member
- Use the fun tips given in week 6 and 8 again and again

"Your visions will become clear only when you can look into your own heart. Who looks outside, dreams; who looks inside, awakes."
Carl Jung, founder of analytical psychology

RETURN TO HOME SCREEN

© 2025 Kókai LL et al. *JAMA Network Open*.

Get Activated module content

Week 1

Getting started
Welcome to your first Get Activated activity! Get ready to revolutionize the way you achieve your physical activity Goals!

Each week in this activity, you:

1. Set a Goal.
2. Can make a Commitment to your Goal.
3. Make Action Plans detailing how you will reach your Goal.

Additionally, in the first four weeks of Get Activated, we will give you guidance on how to design your own Commitments. In the last four weeks, you will get the opportunity to make Coping Plans.

Commitment, Action Planning and Coping Planning are proven tools that will help you achieve your Goal.

Of course, we will explain how to use these tools as we go along to make each step of the way as easy as possible.

Goal Setting

Give *Goal Setting* a try!

At the start of each week, you will set a Goal for your physical activity for that week in terms of Active Zone Minutes.

At least 150 Active Zone Minutes per week is necessary in order to meet the physical activity recommendations of the World Health Organization.

ⓘ Physical activity guidelines and Active Zone Minutes

Physical activity guidelines and Active Zone Minutes
The World Health Organization recommends that adults do at least 150 minutes of moderate physical activity, 75 minutes of vigorous physical activity, or an equivalent combination of moderate- and vigorous activity throughout the week.
Examples of moderate activity are walking or cycling briskly, and water aerobics. Examples of vigorous activity are jogging, jumping rope, and swimming laps.

© 2025 Kókai LL et al. *JAMA Network Open*.

Every minute that you spend doing moderate or vigorous activities will be registered by your Fitbit Inspire 2 as Active Zone Minutes. The fat burn heart rate zone corresponds to moderate activity. The cardio and peak heart rate zones correspond to vigorous activity, and are counted double!

Setting realistic goals

However, if you think 150 Active Zone Minutes is an unrealistic Goal for you, you can set a lower Goal. Just make sure that your Goal is high enough to be both challenging and inspiring!

Here are some examples of Goals you could set, given your current physical activity level:

Your current physical activity level	Example Goal for this week
New to physical activity	30 minutes
Starting to get more active	60 minutes
Somewhat active	120 minutes
Regularly active	180 minutes
Highly active person	300 minutes

Remember that these are just examples and that, in general, the more physical activity you do, the better!

Now you can set your Goal for this week.

How many Active Zone Minutes do you aim to do this week (Monday-Sunday)?
My Goal for this week is:
___ Active Zone Minutes

Commitment Training – i2be Points Commitment

Well done on setting your physical activity Goal for the week!

Now we give you the chance to make a Commitment to help you achieve this Goal.

A Commitment is an agreement you make with yourself, or with others, so that you will get a reward, or avoid suffering a penalty, only if you meet your Goal. A strong Commitment will give you an added incentive to achieve your Goal.

You can make a Commitment to your Goal using 40 of your i2be Points. We call this the i2be Points Commitment.
ⓘ i2be Points and Rewards

© 2025 Kókai LL et al. *JAMA Network Open*.

---

i2be Points and Rewards

You earn *i2be Points* every time you complete an activity in the i2be app.

With every 100 points you earn, you move to a higher *Bee Level*.

You win the *Fitbit prize* if you reach the Queen Bee level (300 i2be Points) by the end of the i2be study. Winning the Fitbit prize means that you get to keep the Fitbit device you have been given! If you don't reach 300 i2be Points by the end of the study, you will have to return the Fitbit device to us by mail.

During the 8-week i2be program, the *i2be raffle* is held every week, giving you the chance to win a €25 shopping voucher. An i2be raffle is also held during the three follow-up weeks. You qualify for the raffle in a given week by gaining all i2be points for that week in the i2be app.

The i2be Points Commitment works as follows

- You pay a deposit of 40 i2be Points now. The points will be automatically deducted from your total.
- If you meet your Goal this week, as verified by your Fitbit data, you will be repaid the deposit. The points will be automatically added back to your total by the end of week 2 (and in time to be counted for the week 1 i2be Raffle which will be held at the end of week 2)
- If you do not meet your Goal, you will not be repaid the deposit. The consequences of this for you would be:
    - You can't enter the week 1 i2be Raffle
    - More difficult for you to earn enough points to receive the Fitbit Prize by the end of the i2be program.

Give the i2be Points Commitment a try!

Do you want to make this commitment?

☐ Yes ☐ No

Make sure to sync your Fitbit app early next week, ideally next Monday, so that we can verify if you have met your week 1 Goal.

Action Planning

Now we give you the chance to make *Action Plans* to help you achieve your physical activity Goal.

Action Plans are very specific plans on how to achieve your physical activity Goals. They specify several aspects of your planned physical activity:

- What type of physical activity will you do?
- How long will you do it for (Active Zone minutes)?
- When will you do it?
- Where will you do it?

© 2025 Kókai LL et al. *JAMA Network Open*.

- With whom will you do it?

Example
Judith has set a Goal of 120 Active Zone Minutes of physical activity for this week. She then makes two Action Plans specifying how she will achieve this goal:

Action Plan 1
- What type of physical activity? Running
- How long? 30 Active Zone Minutes
- When? Tuesday at 09:30
- Where? Gym
- With whom? Friend - Ellen

Action Plan 2
- What type of physical activity? Cycling
- How long? 90 Active Zone Minutes
- When? Saturday at 16:00
- Where? Forest cycling path
- With whom? Alone

Now give Action Plans a try – on the next page you can make your Action Plans for this week (Monday-Sunday)
Note: You should only make Action Plans for activities that count towards your Active Zone Minutes (i.e. activities that increase your heartrate enough to enter the *Fat Burn*, *Cardio* or *Peak* heart-rate zones). For instance, while yoga and stretching have health benefits, they usually do not count towards your Active Zone Minutes.

Action Plan 1
Your Goal: x Active Zone Minutes
Total per your Action Plan(s): y Active Zone Minutes

What type of physical activity?
Cardio, Cycling, HIIT, Running, Strength training, Swimming, Tennis, Walking, Other.

How long (in Active Zone Minutes)?
___ Active Zone Minutes

When?
Monday, Tuesday, Wednesday, Thursday, Friday, Saturday, Sunday
hh:mm

Where?
At home, Gym, Public park, Public streets, Sports field, Swimming pool, Tennis court, Other

© 2025 Kókai LL et al. *JAMA Network Open*.

With whom?
Alone, Partner, Friend(s), Family member(s), With a Class, Other.
Add a new Action Plan

You will receive a notification reminding you of the Action Plans you have made.

How soon before the planned exercise do you want to receive the notification?
15 minutes, 30 minutes, 1 hour, 2 hours, 3 hours, 6 hours, 12 hours, 18 hours, 24 hours.

Commitment training – Design your own Commitment
Well done on making your Action Plans for the week!

You have already had the chance to make the i2be Points Commitment. Now, before we finish Get Activated for this week, you can learn a little about designing your own Commitments.

For the next four weeks, you will be guided on how to design your own Commitments, focusing on a different type of Commitment each week.

Reward Commitment
One popular type of commitment is a *Reward Commitment*. This is where you promise to give yourself a reward if you reach your Goal.

For example, you can promise yourself that you will go to the cinema, visit a spa, or buy yourself some new clothes if you meet your Goal. It can also be a reward that has consequences for not just yourself, but also others. For example, you will do a fun activity with your family *only* if you meet your Goal.

A Reward Commitment is a different type of commitment than the i2be Points Commitment, which is a *Penalty* Commitment. A penalty commitment is where you promise to impose a *penalty* on yourself if you *don't* reach your Goal. We will discuss this type of commitment next week.

Give designing your own Reward Commitment a try!
Even if you have already made the i2be Points Commitment, it is worthwhile doing this to learn how to design your own Commitments.

Think of a reward that is appealing enough so that it spurs you to really make the effort to reach your Goal. Make an agreement with yourself that you will give yourself this reward *only* if you meet your Goal this week.

Please enter here the reward you will get if you reach your Goal:

Wrap-up
Well done on designing your own Reward Commitment!

You have completed week 1 of Get Activated – you are on your way to Goal attainment!

See you next week!

RETURN TO HOME SCREEN

© 2025 Kókai LL et al. *JAMA Network Open*.

Week 2

Goal Setting
Now you can set your physical activity *Goal* for this week.

A good idea is to set a Goal to do more Active Zone Minutes than what you did last week. You can check how many Active Zone Minutes you did last week in the *Goals* section of this app.

How many Active Zone Minutes do you aim to do this week (Monday-Sunday)?
My Goal for this week is:
___ Active Zone Minutes

Commitment Training – i2be Points Commitment
Well done on setting your physical activity Goal for the week!

Now you can make the i2be Points Commitment.
This works as follows:

- You pay a deposit of 40 i2be Points now. The points will be automatically deducted from your total.
- If you meet your Goal this week, as verified by your Fitbit data, you will be repaid the deposit. The points will be automatically added back to your total by the end of week 3 (and in time to be counted for the week 2 i2be Raffle which will be held at the end week 3)
- If you do not meet your Goal, you will not be repaid the deposit. The consequences of this for you would be:
    - You can't enter the week 2 i2be Raffle
    - More difficult for you to earn enough points to receive the Fitbit Prize by the end of the i2be program.

Give the i2be points commitment a try!

Do you want to make this commitment?
☐ Yes ☐ No
Make sure to sync your Fitbit app early next, ideally on Monday, so that we can verify if you have met your week 2 Goal.

Action Planning
Now you can make your *Action Plans* specifying how you will achieve your physical activity Goal for this week (Monday-Sunday).

Action Plan 1

Your Goal: x Active Zone Minutes
Total per your Action Plan(s): y Active Zone Minutes

What type of physical activity?
Cardio, Cycling, HIIT, Running, Strength training, Swimming, Tennis, Walking, Other.

How long (in Active Zone Minutes)?
___ Active Zone Minutes

When?
Monday, Tuesday, Wednesday, Thursday, Friday, Saturday, Sunday
hh:mm

Where?
At home, Gym, Public park, Public streets, Sports field, Swimming pool, Tennis court, Other

With whom?
Alone, Partner, Friend(s), Family member(s), With a Class, Other.
Add a new Action Plan

You will receive a notification reminding you of the Action Plans you have made.

How soon before the planned exercise do you want to receive the notification?
15 minutes, 30 minutes, 1 hour, 2 hours, 3 hours, 6 hours, 12 hours, 18 hours, 24 hours.
.

Commitment Training – Design your own Commitment

Now, before we finish Get Activated for this week, you can learn a little more about *designing your own Commitments*.

Last week, you learned about Reward Commitments. This week, we will focus on Penalty Commitments. This is where you promise to impose a penalty on yourself if you *don't* reach your Goal.

For example, you won't get to watch your favourite show for a week, or you will have to wear an item of clothing you really don't like for a day. It can also be a penalty that has consequences for not just yourself, but also others. For example, you block Netflix for everyone in your family for a day if you *don't* meet your Goal.

The i2be Points Commitment is a great example of a Penalty Commitment – you make an agreement that a penalty of losing 40 points will be imposed on you if you don't reach your goal.

Give designing your own Penalty Commitment a try!

© 2025 Kókai LL et al. *JAMA Network Open*.

Even if you have already made the i2be Points Commitment, it is worthwhile doing this to learn how to design your own Commitments.

Think of a penalty that is strong enough (i.e. you dislike it enough) so that it spurs you to really make the effort to reach your Goal. Make an agreement with yourself that you will impose this penalty on yourself if you do not meet your Goal this week.

Please enter here the penalty you will avoid if you reach your Goal:

Wrap-up
Well done on designing your own Penalty Commitment!

You have completed week 2 of Get Activated – great going!

See you next week!

RETURN TO HOME SCREEN

© 2025 Kókai LL et al. *JAMA Network Open*.

Week 3

Goal Setting
Now you can set your physical activity *Goal* for this week.

A good idea is to set a Goal to do more Active Zone Minutes than what you did last week. You can check how many Active Zone Minutes you did last week in the *Goals* section of this app.

How many Active Zone Minutes do you aim to do this week (Monday-Sunday)?
My Goal for this week is:
___ Active Zone Minutes

Commitment Training – i2be Points Commitment
Well done on setting your physical activity Goal for the week!

Now you can make the i2be Points Commitment.
This works as follows:

- You pay a deposit of 40 i2be Points now. The points will be automatically deducted from your total.
- If you meet your Goal this week, as verified by your Fitbit data, you will be repaid the deposit. The points will be automatically added back to your total by the end of week 4 (and in time to be counted for the week 3 i2be Raffle which will be held at the end week 4)
- If you do not meet your Goal, you will not be repaid the deposit. The consequences of this for you would be:

    - You can't enter the week 3 i2be Raffle
    - More difficult for you to earn enough points to receive the Fitbit Prize by the end of the i2be program.

Give the i2be points commitment a try!

Do you want to make this commitment?
☐ Yes ☐ No
Make sure to sync your Fitbit app early next, ideally on Monday, so that we can verify if you have met your week 3 Goal.

Action Planning
Now you can make your *Action Plans* specifying how you will achieve your physical activity Goal for this week (Monday-Sunday).

Tip: Involve others – make plans together! Besides being more fun, they are also harder to skip.

© 2025 Kókai LL et al. *JAMA Network Open*.

Action Plan 1
Your Goal: x Active Zone Minutes
Total per your Action Plan(s): y Active Zone Minutes

What type of physical activity?
Cardio, Cycling, HIIT, Running, Strength training, Swimming, Tennis, Walking, Other.

How long (in Active Zone Minutes)?
___ Active Zone Minutes

When?
Monday, Tuesday, Wednesday, Thursday, Friday, Saturday, Sunday
hh:mm

Where?
At home, Gym, Public park, Public streets, Sports field, Swimming pool, Tennis court, Other

With whom?
Alone, Partner, Friend(s), Family member(s), With a Class, Other.
Add a new Action Plan

You will receive a notification reminding you of the Action Plans you have made.

How soon before the planned exercise do you want to receive the notification?
15 minutes, 30 minutes, 1 hour, 2 hours, 3 hours, 6 hours, 12 hours, 18 hours, 24 hours.
.

Commitment Training – Design your own Commitment
Well done on making your Action Plans for the week!

Now, before we finish Get Activated for this week, you can learn a little more about *designing your own Commitments*.

Over the last two weeks, you learned about both Reward and Penalty Commitments. This week, you can make your Reward or Penalty Commitments even stronger by making yourself *accountable to others*. We call this *Accountability* Commitment.

For example, make a promise to your partner, family member, or friend that you will only give yourself a specified reward, or that you will only avoid imposing a penalty on yourself, if you meet your Goal.

Being accountable to others

© 2025 Kókai LL et al. *JAMA Network Open*.

In certain circumstances, you can even give your partner, family member, or friend the responsibility for implementing the reward or penalty. For example, you could get your partner to promise to bring you for a nice meal (a reward) if you meet your Goal. Another example: you could tell a family member to hide the TV remote control from you for a day (a penalty) if you don't meet your Goal.

The i2be Points Commitment is a great example of this type of commitment – you have given us responsibility for implementing the penalty of losing 40 points if you don't reach your Goal.

Give designing your own Accountability Commitment a try! Even if you have already made the i2be Points Commitment, it is worthwhile doing this to learn how to design your own Commitment.

Please enter here the reward you will get, or penalty you will avoid, if you meet your Goal

Please enter here how you will be accountable to others (e.g. a promise), and to whom will you be accountable?

Wrap-up

Well done on designing your own Accountability Commitment!

You have completed week 3 of Get Activated – great work!

See you next week!

RETURN TO HOME SCREEN

© 2025 Kókai LL et al. *JAMA Network Open*.

Week 4

Goal Setting
Now you can set your physical activity *Goal* for this week.

A good idea is to set a Goal to do more Active Zone Minutes than what you did last week. You can check how many Active Zone Minutes you did last week in the *Goals* section of this app.

How many Active Zone Minutes do you aim to do this week (Monday-Sunday)?
My Goal for this week is:
___ Active Zone Minutes

Commitment Training – i2be Points Commitment
Well done on setting your physical activity Goal for the week!

Now you can make the i2be Points Commitment.
This works as follows:

- You pay a deposit of 40 i2be Points now. The points will be automatically deducted from your total.
- If you meet your Goal this week, as verified by your Fitbit data, you will be repaid the deposit. The points will be automatically added back to your total by the end of week 5 (and in time to be counted for the week 4 i2be Raffle which will be held at the end week 5)
- If you do not meet your Goal, you will not be repaid the deposit. The consequences of this for you would be:
    - You can't enter the week 4 i2be Raffle
    - More difficult for you to earn enough points to receive the Fitbit Prize by the end of the i2be program.

Give the i2be points commitment a try!

Do you want to make this commitment?
☐ Yes ☐ No
Make sure to sync your Fitbit app early next, ideally on Monday, so that we can verify if you have met your week 4 Goal.

Action Planning
Now you can make your *Action Plans* specifying how you will achieve your physical activity Goal for this week (Monday-Sunday).

Action Plan 1

© 2025 Kókai LL et al. *JAMA Network Open*.

Your Goal: x Active Zone Minutes
Total per your Action Plan(s): y Active Zone Minutes

What type of physical activity?
Cardio, Cycling, HIIT, Running, Strength training, Swimming, Tennis, Walking, Other.

How long (in Active Zone Minutes)?
___ Active Zone Minutes

When?
Monday, Tuesday, Wednesday, Thursday, Friday, Saturday, Sunday
hh:mm

Where?
At home, Gym, Public park, Public streets, Sports field, Swimming pool, Tennis court, Other

With whom?
Alone, Partner, Friend(s), Family member(s), With a Class, Other.
Add a new Action Plan

You will receive a notification reminding you of the Action Plans you have made.

How soon before the planned exercise do you want to receive the notification?
15 minutes, 30 minutes, 1 hour, 2 hours, 3 hours, 6 hours, 12 hours, 18 hours, 24 hours.
.

Commitment Training – Design your own Commitment

Wrapping it all up

Well done on making your Action Plans for the week!

This is the final week in which we give you guidance on designing your own Commitments. If you found Commitments effective to help you reach your Goals, then we encourage you to keep using this technique.

Key elements of Commitment
When designing your own Commitments in the future, remember to include the following elements:

1. Your Goal - this should be measurable and have a specific time frame (e.g. number of Active Zone Minutes per week)
2. How you will monitor progress towards your Goal (e.g. with your Fitbit)
3. The reward you will get, or the penalty you will avoid, if you meet your Goal

© 2025 Kókai LL et al. *JAMA Network Open*.

4. The person or people to whom you will be accountable (e.g. yourself only, partner, family member, friend) and how you will be accountable to them (e.g. a promise).

Tip: Writing down the details of your Commitment (i.e. the four elements above) can help make your Commitment even stronger.

Tip: You can return to the Get Activated activities you have completed in this app any time for the next 12 months to review your previous Commitments and the guidance we have provided (but please note that the answer boxes in these activities will not open up again).

Now design your Commitment for this week below.

Please enter here the reward you will get, or penalty you will avoid, if you meet your Goal

Please enter here how you will be accountable to others (e.g. a promise), and to whom will you be accountable?

Wrap-up
You have completed week 4 of Get Activated – great work!

As mentioned, this is the final week in which we give you guidance you on designing your own Commitments. We wish you the very best of luck in using this technique on your own in the future!

Note though that only the guidance on designing your own Commitments ends this week, and that the i2be Points Commitment will still be available to you in each of the next 4 weeks.

From next week onwards, we will guide you on how to use another proven tool that can help you achieve your goals - *Coping Plans*.

See you next week!

RETURN TO HOME SCREEN

© 2025 Kókai LL et al. *JAMA Network Open*.

Week 5

Goal Setting
Now you can set your physical activity *Goal* for this week.

A good idea is to set a Goal to do more Active Zone Minutes than what you did last week. You can check how many Active Zone Minutes you did last week in the *Goals* section of this app.

How many Active Zone Minutes do you aim to do this week (Monday-Sunday)?
My Goal for this week is:
___ Active Zone Minutes

Commitment Training – i2be Points Commitment
Well done on setting your physical activity Goal for the week!

Now you can make the i2be Points Commitment.
This works as follows:

- You pay a deposit of 40 i2be Points now. The points will be automatically deducted from your total.
- If you meet your Goal this week, as verified by your Fitbit data, you will be repaid the deposit. The points will be automatically added back to your total by the end of week 6 (and in time to be counted for the week 5 i2be Raffle which will be held at the end week 6)
- If you do not meet your Goal, you will not be repaid the deposit. The consequences of this for you would be:

    - You can't enter the week 5 i2be Raffle
    - More difficult for you to earn enough points to receive the Fitbit Prize by the end of the i2be program.

Give the i2be points commitment a try!

Do you want to make this commitment?
☐ Yes ☐ No
Make sure to sync your Fitbit app early next, ideally on Monday, so that we can verify if you have met your week 5 Goal.

Action Planning
Now you can make your *Action Plans* specifying how you will achieve your physical activity Goal for this week (Monday-Sunday).

© 2025 Kókai LL et al. *JAMA Network Open*.

Action Plan 1
Your Goal: x Active Zone Minutes
Total per your Action Plan(s): y Active Zone Minutes

What type of physical activity?
Cardio, Cycling, HIIT, Running, Strength training, Swimming, Tennis, Walking, Other.

How long (in Active Zone Minutes)?
___ Active Zone Minutes

When?
Monday, Tuesday, Wednesday, Thursday, Friday, Saturday, Sunday
hh:mm

Where?
At home, Gym, Public park, Public streets, Sports field, Swimming pool, Tennis court, Other

With whom?
Alone, Partner, Friend(s), Family member(s), With a Class, Other.
Add a new Action Plan

You will receive a notification reminding you of the Action Plans you have made.

How soon before the planned exercise do you want to receive the notification?
15 minutes, 30 minutes, 1 hour, 2 hours, 3 hours, 6 hours, 12 hours, 18 hours, 24 hours.

Coping Planning
Well done on making your Action Plans for the week!

This week we introduce you to another proven tool to help you achieve your Goals – *Coping Plans*.

Coping Plans can help you follow through with your Action Plans and thereby reach your Goals. They do this by getting you to:

- Think of likely setbacks, challenges and obstacles that you will have to cope with while carrying out your Action Plans.
- Think of solutions to such issues in advance.

An example of a set of Coping Plans would be:

- If Ellen cancels on our planned running session at the gym, then I will go by myself.
- If I am too tired to go running at 09:30, then I will go at 16:00 instead.

© 2025 Kókai LL et al. *JAMA Network Open*.

- If it is raining at the time of my planned cycle through the forest, then I will go to a spinning class instead.
- If I find cycling through the forest unpleasant, then (next time) I will choose a more pleasant form of exercise, like swimming.

Give Coping Plans a try!
At the beginning of each week from now until the end of the i2be program, you will make Coping Plans to help you follow through with your Action Plans and thereby reach your Goal for that week.

Give Coping Plans a try for this week (Monday-Sunday)!

Coping Plan 1
Issue that I might have to cope with in order to stick to my Action Plans this week:

The way I Plan to Cope with this issue:

Add a new Coping Plan

Wrap-up
Well done on creating your Coping Plans for the week!

You have completed week 5 of Get Activated – this is what champions are made of!

Don’t forget: If you found designing your own Commitment useful in the first 4 weeks, continue using this tool now on your own to give you an extra boost toward reaching your Goal!

See you next week!

Week 6

Goal Setting
Now you can set your physical activity *Goal* for this week.

A good idea is to set a Goal to do more Active Zone Minutes than what you did last week. You can check how many Active Zone Minutes you did last week in the *Goals* section of this app.

How many Active Zone Minutes do you aim to do this week (Monday-Sunday)?
My Goal for this week is:
___ Active Zone Minutes

Commitment Training – i2be Points Commitment
Well done on setting your physical activity Goal for the week!

Now you can make the i2be Points Commitment.
This works as follows:

- You pay a deposit of 40 i2be Points now. The points will be automatically deducted from your total.
- If you meet your Goal this week, as verified by your Fitbit data, you will be repaid the deposit. The points will be automatically added back to your total by the end of week 7 (and in time to be counted for the week 6 i2be Raffle which will be held at the end week 7)
- If you do not meet your Goal, you will not be repaid the deposit. The consequences of this for you would be:
    - You can't enter the week 6 i2be Raffle
    - More difficult for you to earn enough points to receive the Fitbit Prize by the end of the i2be program.

Give the i2be points commitment a try!

Do you want to make this commitment?
☐ Yes ☐ No
Make sure to sync your Fitbit app early next, ideally on Monday, so that we can verify if you have met your week 6 Goal.

Action Planning
Now you can make your *Action Plans* specifying how you will achieve your physical activity Goal for this week (Monday-Sunday).

© 2025 Kókai LL et al. *JAMA Network Open*.

Action Plan 1
Your Goal: x Active Zone Minutes
Total per your Action Plan(s): y Active Zone Minutes

What type of physical activity?
Cardio, Cycling, HIIT, Running, Strength training, Swimming, Tennis, Walking, Other.

How long (in Active Zone Minutes)?
___ Active Zone Minutes

When?
Monday, Tuesday, Wednesday, Thursday, Friday, Saturday, Sunday
hh:mm

Where?
At home, Gym, Public park, Public streets, Sports field, Swimming pool, Tennis court, Other

With whom?
Alone, Partner, Friend(s), Family member(s), With a Class, Other.
Add a new Action Plan

You will receive a notification reminding you of the Action Plans you have made.

How soon before the planned exercise do you want to receive the notification?
15 minutes, 30 minutes, 1 hour, 2 hours, 3 hours, 6 hours, 12 hours, 18 hours, 24 hours.

Coping Planning
Well done on making your Action Plans for the week!

Now you can make your *Coping Plans* for this week (Monday-Sunday).

Coping Plan 1
Issue that I might have to cope with in order to stick to my Action Plans this week:

The way I Plan to Cope with this issue:

Add a new Coping Plan

Wrap-up
Well done on making your Coping Plans for the week!

© 2025 Kókai LL et al. *JAMA Network Open*.

You have completed week 6 of Get Activated – great going!

Don’t forget: If you found designing your own Commitment useful in the first 4 weeks, continue using this tool now on your own to give you an extra boost toward reaching your Goal!

See you next week!

RETURN TO HOME SCREEN

© 2025 Kókai LL et al. *JAMA Network Open*.

Week 7

Goal Setting
Now you can set your physical activity *Goal* for this week.

A good idea is to set a Goal to do more Active Zone Minutes than what you did last week. You can check how many Active Zone Minutes you did last week in the *Goals* section of this app.

How many Active Zone Minutes do you aim to do this week (Monday-Sunday)?
My Goal for this week is:
___ Active Zone Minutes

Commitment Training – i2be Points Commitment
Well done on setting your physical activity Goal for the week!

Now you can make the i2be Points Commitment.
This works as follows:

- You pay a deposit of 40 i2be Points now. The points will be automatically deducted from your total.
- If you meet your Goal this week, as verified by your Fitbit data, you will be repaid the deposit. The points will be automatically added back to your total by the end of week 8 (and in time to be counted for the week 7 i2be Raffle which will be held at the end week 8)
- If you do not meet your Goal, you will not be repaid the deposit. The consequences of this for you would be:

    - You can’t enter the week 7 i2be Raffle
    - More difficult for you to earn enough points to receive the Fitbit Prize by the end of the i2be program.

Give the i2be points commitment a try!

Do you want to make this commitment?
☐ Yes ☐ No
Make sure to sync your Fitbit app early next, ideally on Monday, so that we can verify if you have met your week 7 Goal.

Action Planning
Now you can make your *Action Plans* specifying how you will achieve your physical activity Goal for this week (Monday-Sunday).

© 2025 Kókai LL et al. *JAMA Network Open*.

Action Plan 1
Your Goal: x Active Zone Minutes
Total per your Action Plan(s): y Active Zone Minutes

What type of physical activity?
Cardio, Cycling, HIIT, Running, Strength training, Swimming, Tennis, Walking, Other.

How long (in Active Zone Minutes)?
___ Active Zone Minutes

When?
Monday, Tuesday, Wednesday, Thursday, Friday, Saturday, Sunday
hh:mm

Where?
At home, Gym, Public park, Public streets, Sports field, Swimming pool, Tennis court, Other

With whom?
Alone, Partner, Friend(s), Family member(s), With a Class, Other.
Add a new Action Plan

You will receive a notification reminding you of the Action Plans you have made.

How soon before the planned exercise do you want to receive the notification?
15 minutes, 30 minutes, 1 hour, 2 hours, 3 hours, 6 hours, 12 hours, 18 hours, 24 hours.

Coping Planning
Well done on making your Action Plans for the week!

Now you can make your *Coping Plans* for this week (Monday-Sunday).

Coping Plan 1
Issue that I might have to cope with in order to stick to my Action Plans this week:

The way I Plan to Cope with this issue:

Add a new Coping Plan

© 2025 Kókai LL et al. *JAMA Network Open*.

Wrap-up
Well done on making your Coping Plans for the week!

You have completed week 7 of Get Activated – what an achievement!

Don't forget: If you found designing your own Commitment useful in the first 4 weeks, continue using this tool now on your own to give you an extra boost toward reaching your Goal!

See you next week!

RETURN TO HOME SCREEN

© 2025 Kókai LL et al. *JAMA Network Open*.

Week 8

Goal Setting
Now you can set your physical activity *Goal* for this week.

A good idea is to set a Goal to do more Active Zone Minutes than what you did last week. You can check how many Active Zone Minutes you did last week in the *Goals* section of this app.

How many Active Zone Minutes do you aim to do this week (Monday-Sunday)?
My Goal for this week is:
___ Active Zone Minutes

Commitment Training – i2be Points Commitment
Well done on setting your physical activity Goal for the week!

Now you can make the i2be Points Commitment.
This works as follows:

- You pay a deposit of 40 i2be Points now. The points will be automatically deducted from your total.
- If you meet your Goal this week, as verified by your Fitbit data, you will be repaid the deposit. The points will be automatically added back to your total by the end of week 9 (and in time to be counted for the week 8 i2be Raffle which will be held at the end week 9).
- If you do not meet your Goal, you will not be repaid the deposit. The consequences of this for you would be:
    - You can't enter the week 8 i2be Raffle
    - More difficult for you to earn enough points to receive the Fitbit Prize by the end of the i2be program.

Give the i2be points commitment a try!

Do you want to make this commitment?
☐ Yes ☐ No
Make sure to sync your Fitbit app early next, ideally on Monday, so that we can verify if you have met your week 8 Goal.

Using Commitments in the future
As this is the final week of the i2be program, this is the last week in which you can make the i2be Points Commitment.

© 2025 Kókai LL et al. *JAMA Network Open*.

However, if you found Commitments useful to help you achieve your Goals, we encourage you to continue using this tool by designing your own Commitments, as we guided you through in the first four weeks of Get Activated.

In this app, you can return to the Get Activated activities you completed in those first four weeks any time for the next 12 months to review the Commitments you designed and the guidance we provided (but please note that the answer boxes will not open up again in these activities).

Action Planning
Now you can make your *Action Plans* specifying how you will achieve your physical activity Goal for this week (Monday-Sunday).

Action Plan 1
Your Goal: x Active Zone Minutes
Total per your Action Plan(s): y Active Zone Minutes

What type of physical activity?
Cardio, Cycling, HIIT, Running, Strength training, Swimming, Tennis, Walking, Other.

How long (in Active Zone Minutes)?
___ Active Zone Minutes

When?
Monday, Tuesday, Wednesday, Thursday, Friday, Saturday, Sunday
hh:mm

Where?
At home, Gym, Public park, Public streets, Sports field, Swimming pool, Tennis court, Other

With whom?
Alone, Partner, Friend(s), Family member(s), With a Class, Other.
Add a new Action Plan

You will receive a notification reminding you of the Action Plans you have made.

How soon before the planned exercise do you want to receive the notification?
15 minutes, 30 minutes, 1 hour, 2 hours, 3 hours, 6 hours, 12 hours, 18 hours, 24 hours.

Coping Planning
Well done on making your Action Plans for the week!

© 2025 Kókai LL et al. *JAMA Network Open*.

Now you can make your *Coping Plans* for this week (Monday-Sunday).

Coping Plan 1
Issue that I might have to cope with in order to stick to my Action Plans this week:

The way I Plan to Cope with this issue:

Add a new Coping Plan

Using Action and Coping planning in the future
Well done on making your Coping Plans for the week!
This is the last week of the i2be program, and therefore the last week in which we help you make Action Plans and Coping Plans.
Did you find Action Plans and Coping Plans useful to help you meet your physical activity goals? If so, we recommend that you continue using them in the future.

Some extra ideas for how to make great Action Plans and Coping Plans

1. Writing down the details of your Action Plans and Coping Plans is important to make these plans even stronger
2. Make your weekly plans easier to remember by writing them on a sticky note, and sticking them on your fridge.
3. Adapt and overcome – if you have been struggling to stick to your planning, perhaps you need to change your planning (e.g. try a different time of day or a different location). There are many roads that lead to success!
4. Involve others – make plans together! Besides being more fun, they are also harder to skip.
5. Plan ahead – perhaps you are the type of person that prefers to plan for the next week instead of the current? That way, you can organize your week around your physical activity plans – nothing crossing your schedule!

Tip: You can return to the Get Activated activities you have completed in this app any time for the next 12 months to review your previous Action Plans and Coping Plans (but please note that the answer boxes will not open up again in these activities).

Wrap-up
This is the last week of the i2be program. Well done on completing all Get Activated activities! We hope that the tools you have learned here – Commitment to a Goal, Action Plans and Coping Plans - can help you achieve your goals now and in the future. Good luck!

RETURN TO HOME SCREEN

Get Energized module content

Week 1

Good things
Welcome to your first Get Energized activity! We are pleased that you have committed to i2be for the coming eight weeks. Your journey to discover the power of an energized state of mind and body will be one to remember. You can always access the Get Energized activities of past weeks if you would like to re-read your answers.

Let's get started!

There is nothing you can get that will lead you to be present, stable, and at ease.

The goal of Get Energized activities is to reveal that you are present, stable, and at ease. This means that these qualities are already within you.

Negative thoughts and feelings that you may experience from time to time, such as doubt, fear, anxiety, and anger, are like clouds. They come and go, always moving and changing. *They are not lasting parts of you*.

On the other hand, your true nature is like the sky. It is always present, stable, and at ease, even when obscured by clouds. *Your true nature is everlasting*.

Begin with the premise that the best version of yourself is already located deep within, so Get Energized can become less about trying, doing, and getting, and more about simply relaxing, releasing, and letting go.

In order to fully experience Get Energized, your mind as well as your body should be in a relaxed state. To allow for this, each week's activity will start with a grounding activity. Grounding activities bring you into contact with the present moment – *the here and now*. The grounding activity of this week will allow you to connect to your true nature.

Each week's grounding activity will be an audio clip of about 4 minutes – so get comfortable, put on some headphones if you like, and press play below.

#Press play to hear the following text:

- Take a seat in a comfortable, quiet place. Uncross your legs and arms.
- Close your eyes, or keep a soft gaze towards the ground in front of you; lengthen your spine, and release your shoulders.

© 2025 Kókai LL et al. *JAMA Network Open*.

- Breathe slowly and evenly in and out through your nose.
- Once you are settled, notice how thoughts and feelings never cease to arise and dissipate, come and go.
- Notice too, how you can watch these thoughts and feelings, as if from a distance.
- There's a separation between the part of you that's *doing* the thinking and feeling, and the part of you that *notices* this thinking and feeling.
- This is the distance between the "clouds" of your mind, temporary thoughts and feelings; and the "sky" of your mind, your true nature, always present, watching your thoughts and feelings come and go.
- Turn further inward, towards your true nature that is always present: the watcher, the witness, the observer. Stay present with your true nature, rather than the thoughts or the emotions that come and go.
- When thoughts and emotions arise, take notice of them, without judging or labelling them as good or bad, pleasant or unpleasant. Gently take your focus back to your true nature: the watcher, the witness, the observer.
- Rest in the presence of this deep awareness, your most inner sense of self, until you hear a chime, indicating the end of this grounding activity.

#End of audio clip

Well done on relaxing your mind and body by getting in touch with your true nature.

Was it difficult to differentiate between the part of you that's *doing* the thinking, and the part of you that *notices* this thinking?
Write here...

Sometimes, the events of our lives are clearly *good* – such as hard work finally paying off; or clearly *bad* – such as the loss of a loved one. More often, however, events have several sides to them. For example, it could happen that you miss the train (*bad*), which gives you time to have a coffee in the sun (*good*). Appreciating the good things in your life will make you feel present, stable, and at ease.

We would like to invite you to write down three good things that happen to you *within one day*. These events can be big or small, it only matters that you feel a glow when you think about them: for example, a laugh with a friend, a peaceful moment to yourself, a tasty meal, or a sunny afternoon outside.

Do this activity today!
[If that is not possible, plan to do it on another specific day this week; Saturday at the latest.]
Afterwards, return to Get Energized to complete the activities below.

First good thing
Write here...

Second good thing
Write here...

© 2025 Kókai LL et al. *JAMA Network Open*.

Third good thing
Write here...

Good job on evaluating some daily events positively!

How did it feel to purposefully look for the good in things?
Write here...

When we turn our mind inward, we recognize that our true nature is present, stable, and at ease. Just as the sky is never altered by the clouds. If you have enjoyed this activity, you are invited to get in touch with your true nature, and note down good things whenever you feel like it.

RETURN TO HOME SCREEN

Week 2

Kindness
Welcome to your weekly dose of positivity! We will start with a grounding activity to put your mind and body at ease. We are going to practice loving kindness: the desire for someone else to be happy, or for yourself to be happy.

Just like last week, the grounding activity will be an audio clip of about 4 minutes – so get comfortable, put on some headphones if you like, and press play below.

#Press play to hear the following text:

- Take a seat in a comfortable, quiet place. Uncross your legs and arms.
- Close your eyes, or keep a soft gaze towards the ground in front of you; lengthen your spine, and release your shoulders.
- Breathe slowly and evenly in and out through your nose.
- Once you are settled, bring to mind someone who makes you feel happy; a relative, close friend, spouse, child or pet.
- Have a sense of him or her being in front of you - feel them, sense them, see them.
- As you imagine him or her, notice how you're feeling inside - maybe you feel some warmth on your skin, a smile on your face, a sense of expansiveness in your heart. This is Loving Kindness. This feeling is accessible to all of us at any moment.
- Now that you have this loved one in front of your mind's eye, begin to wish him or her well by saying the following four sentences out loud, as written on your screen:
  May you be safe and protected from danger
  May you be happy and peaceful
  May you be healthy and strong
  May you have ease and well-being
- You can say these words, or you can say other words that have even more meaningful to you.
- Repeat the four sentences three times, directing them towards your loved one.
- As you are sending out these words and feelings of Loving Kindness, imagine that your loved begins to send these words and feelings back to you – see if you can accept this Loving Kindness, and take it in, as written on your screen:

May *you* be safe and protected from danger
May *you* be happy and peaceful
May *you* be healthy and strong
May *you* have ease and well-being

- Repeat the four sentences three times, directing them towards yourself, in the name of your loved one.
- Rest in the presence of the words and feelings of Loving Kindness, both oriented towards your loved one and yourself, until you hear a chime, indicating the end of this grounding activity.

#End of audio clip

© 2025 Kókai LL et al. *JAMA Network Open*.

May you be safe and protected from danger<br>May you be happy and peaceful<br>May you be healthy and strong<br>May you have ease and well-being

Well done on putting your mind and body at ease through the practice of loving kindness.

How did it feel to send out these words and feelings to your loved one? How did it feel to 'receive' them from your loved one?
Write here...

By shifting our focus from receiving to giving, we can recognize that we have an abundance of good to give. Try going through your day with an attitude of kindness towards others: for example, think positive thoughts towards someone, smile at someone, compliment someone, hold a door open, give a small gift, or write a thank you note.

Purposefully engage in three acts of kindness within one day. Any act of kindness, no matter how large or small, counts. Note down your three acts of kindness.

Do this activity today!
[If that is not possible, plan to do it on another specific day this week; Saturday at the latest.]
Afterwards, return to Get Energized to complete the activities below.

First act of kindness
Write here...

Second act of kindness
Write here...

Third act of kindness
Write here...

Good job on taking the time to be kind to others!

How did it feel to be purposefully kind?
Write here...

We are connected to the people around us at all times, especially to our loved ones. If you have enjoyed this activity, you are invited to perform loving kindness, and note down acts of kindness whenever you want.

RETURN TO HOME SCREEN

© 2025 Kókai LL et al. *JAMA Network Open*.

Week 3

Beautiful sensations
Welcome to week 3 of Get Energized. Let us start with a grounding activity that awakens your senses of hearing, smell, and touch, and that allows you to take things in with curiosity and without judgement.

Get comfortable, put on some headphones if you like, and press play below.

#Press play to hear the following text:

- Take a seat in a comfortable, quiet place. Uncross your legs and arms.
- Close your eyes, or keep a soft gaze towards the ground in front of you; lengthen your spine, and release your shoulders.
- Breathe slowly and evenly in and out through your nose.
- Once you are settled, guide your awareness towards sound. Without 'searching for sound', open your awareness until sounds reach you. What is it that you hear?
- Can you notice sound without judging or labelling it (as good or bad, pleasant or unpleasant)?
- If and when you notice your mind wandering off to the other senses, to thoughts or feelings, simply return your focus to sound. Continue for about a minute.
- Next, guide your awareness towards smell. Without 'searching for smells', open your awareness to smells that are present. What is it that you smell?
- Can you notice what you smell without judging or labelling it (as good or bad, pleasant or unpleasant)?
- If and when you notice your mind wandering off to the other senses, to thoughts or feelings, simply return your focus to smell. Continue for about a minute.
- Finally, guide your awareness towards touch. Without 'looking for touching', notice what sensations you can feel through your skin. What is it that you are touching (already)?
- Can you notice what you sense with your skin without judging or labelling it (as good or bad, pleasant or unpleasant)?
- If and when you notice your mind wandering off to the other senses, to thoughts or feelings, simply return your focus to touch. Continue for about a minute.
- Remain present with the experience of touch until you hear a chime, indicating the end of this grounding activity.

#End of audio clip

Well done! Did you arrive at a more serene state of body and mind by zoning in on your senses one by one?

Describe the experience of zoning in on just one of the senses. You can choose to describe hearing, smell, or touch.
Write here...

© 2025 Kókai LL et al. *JAMA Network Open*.

By creating more space between *noticing* your sensations, and *judging or labelling* your sensations, you can purposefully *choose not to* judge or label them at all. This way, you can let go of sensations that you do not wish to experience: they will float away like a cloud.

On the other hand, you can also purposefully *choose to* judge and label your sensations. This can be especially relevant when you wish to experience good or pleasant sensations. You can intensely enjoy them, before they float away like a cloud.

Take a few minutes to find beauty in three sensations right now. For example, do you hear something beautiful? Do you smell something nice? Do you feel something pleasant?

First beautiful thing
Write here…

Second beautiful thing
Write here…

Third beautiful thing
Write here…

You *chose* to judge and label your sensations in order to bask in beauty. Well done!

Did you, at any point during the activity, feel like you are *in the present moment*? If so, could you describe the feeling of being in the present moment?
Write here…

What we notice through our senses does not provide us knowledge on ultimate reality. Rather, we judge and label our sensations based on our past experiences.
Do what makes you feel present, stable and at ease: whether it is choosing not to engage with perceptions and letting them go; or whether it is choosing to judge, label, and savor perceptions.

If you have enjoyed this activity, you are invited to zone in on your senses one by one, and note down beautiful sensations on any other day as well.

RETURN TO HOME SCREEN

© 2025 Kókai LL et al. *JAMA Network Open*.

Week 4

Gratitude
Welcome back! Already on week four of Get Energized, good on you! This week's activity will take you to a place of gratitude – it will open your eyes to all the things that are already going well in your life.

Get comfortable, put on some headphones if you like, and press play below.

#Press play to hear the following text:

- Take a seat in a comfortable, quiet place. Uncross your legs and arms.
- Close your eyes, or keep a soft gaze towards the ground in front of you; lengthen your spine, and release your shoulders.
- Breathe slowly and evenly in and out through your nose.
- Once you are settled, begin by recognizing your good fortune for living in your particular country. A place that is relatively safe, and offers so much opportunity.
- Zoom in further to your particular region, area, and neighborhood. Note all the positive qualities around you.
- Zoom in to the shelter in which you sit. Be aware that not everyone is fortunate enough to have a roof over their heads.
- Notice the clothes you are wearing, or the comfort of the cushions or chair upon which you are sitting.
- Notice your body. Breathing. Functioning.
- Experience gratitude for each of your senses. Hearing, seeing, tasting, smelling, sensing.
- Recognize that you are here right now, becoming grounded. Recognize how rare and precious it is that you have interest in learning, growing, and deepening your awareness.
- Sit with this sense of gratitude until you hear a chime, indicating the end of this grounding activity.

#End of audio clip

Well done on focusing on some of the fundamental things that are going well in your life.

Name three things that you are grateful for.

First thing I am grateful for
Write here...

Second thing I am grateful for
Write here...

Third thing I am grateful for
Write here...

© 2025 Kókai LL et al. *JAMA Network Open*.

For each of these things you are most grateful for, list one action you could take that would demonstrate your gratitude. For example, if you are grateful for your living room, could you keep it more tidy and cozy than you do now?

First grateful action
Write here...

Second grateful action
Write here...

Third grateful action
Write here...

Think of a (living) person to whom you would like to express your gratitude, and write a letter to him/her in the text box below. Try to be specific in describing the way in which his/her actions have made an important difference in your life.
Write here...

Optional follow-up activity

If you wish to share your letter with the person you wrote it to, continue with the activity below.

Arrange a physical visit or Skype call as soon as possible with the person you wrote the letter to, without explaining the purpose. Try to make it as casual as possible.
When you see this person, read your letter slowly (you can access your letter by returning to the previous page). Make sure to speak with expression and to keep eye contact. Allow the other person to react unhurriedly. Reminisce about the specific events that made this person so important to you.

As we begin to build gratitude, we take note of what we have. As our gratitude grows, we become naturally inclined to share our gratitude with others. If you have enjoyed this activity, you are invited to reflect on what you are grateful for, and share your gratitude with others whenever you feel like it.

RETURN TO HOME SCREEN

© 2025 Kókai LL et al. *JAMA Network Open*.

Week 5

Amusing things
Welcome back! Ready to recharge? Humor may be the best medicine - having a good laugh every day is a great way to increase your quality of life. However, when humor is needed the most, you may not have something ready, or stress may keep you from seeing the humor in the situation. In that case, you can use the following grounding activity to get your dose of laughter.

Get comfortable, put on some headphones if you like, and press play below.

#Press play to hear the following text:

- Take a seat in a comfortable, quiet place. Uncross your legs and arms.
- Close your eyes, or keep a soft gaze towards the ground in front of you; lengthen your spine, and release your shoulders.
- Breathe slowly and evenly in and out through your nose.
- Once you are settled, stretch generally to loosen up any physical tension that may hold you back from laughter. Make sure to stretch your arms, shoulders, back, chest, and legs.
- Now, as silly as it seems, start faking laughter. Make yourself go through the motions of a good belly laugh. Soon, the contagiousness of the act of laughter alone will take hold. You'll begin to laugh spontaneously and genuinely. Throw your head back and laugh from the heart!
- Continue to let yourself be amused by the situation you are in, reminisce about light-hearted situations, or just lean into the physicality of laughter – let go of your restraints!
- Sit with this sense of amusement until you hear a chime, indicating the end of this grounding activity.

#End of audio clip

Well done on loosening up and giving yourself a good laugh!

Did you laugh out loud? If so, what sensations did you feel in your body when you laughed out loud?
Write here…

Amusing things happen to us all the time. Allowing yourself to laugh out loud at the smallest things – seeing something unexpected on the street, overhearing a surprising conversation, or just having an amusing thought - will make you feel so light-hearted!

Notice three amusing things within one day. These events can be as small or large as you can imagine – it's the experience of amusement that counts.

Do this activity today!
[If that is not possible, plan to do it on another specific day this week; Saturday at the latest.]

© 2025 Kókai LL et al. *JAMA Network Open*.

Afterwards, return to Get Energized to complete the activities below.

First amusing thing
Write here…

Second amusing thing
Write here…

Third amusing thing
Write here…

Good job on letting humor seep into your everyday life. Every day we laugh is a good day!

What mood were you in the moment *before* you noticed amusing things? What mood were you in the moment *after* you noticed amusing things?
Write here…

The therapeutic benefits of humor and laughter have been known for millennia – it is the bodily manifestation of being lighthearted. If you have enjoyed this activity, you are invited to laugh out loud as often as you can, and reflect on amusing events in your life regularly.

RETURN TO HOME SCREEN

© 2025 Kókai LL et al. *JAMA Network Open*.

Week 6

Savoring
Welcome back for your weekly dose of positivity! You may often find yourself busy, just flying from one task to the other, or even multitasking, in a hurry to finish. To slow down for a change, let's start with a grounding activity of breath awareness.

Get comfortable, put on some headphones if you like, and press play below.

#Press play to hear the following text:

- Take a seat in a comfortable, quiet place. Uncross your legs and arms.
- Close your eyes, or keep a soft gaze towards the ground in front of you; lengthen your spine, and release your shoulders.
- Breathe slowly and evenly in and out through your nose.
- Once you are settled, without trying to change the breath in anyway, notice how effortlessly it flows into and out of your body.
- Pay attention to each subtle movement of the breath. The inhalation, beginning at the nostrils and moving slowly through to the lungs. The slight pause at the end of the inhalation, chest and belly expanded. The exhalation and the way the body contracts or 'falls' as the breath moves back out. The slight pause at the end of the exhalation, chest and belly relaxed.
- Keep this relaxed, open, attention on your breath. If and when your mind wanders, simply observe this, and then bring back your awareness to your breath.
- Sit with this sense of breath awareness until you hear a chime, indicating the end of this grounding activity.

#End of audio clip

Good job on heightening your awareness of your breath.

How did it feel to become aware of your breath?
Write here...

Did your breath change in any way due to the attention you paid to it?
Write here...

Another great way to recharge is to *take it all in*.

Stop, and savor the moment by engaging in one of these simple activities. Try to be mindful of your sensations, thoughts, and feelings while doing the activity.
Sharpen your sensations: Focus deliberately on certain sensations and block out others. For example, listen to a classical masterpiece, or other powerful song, on your headphones with your eyes closed.

© 2025 Kókai LL et al. *JAMA Network Open*.

Share an experience: Seek out a friend or family member to share an experience with, for example going for a walk in nature. Describe to them how you experience the activity, and ask him/her about their experience. Tell him/her how much you value this moment.

Praise yourself: Share your achievements with a friend or family member. Feel your pride authentically – don't make yourself small. Acknowledge all the hard work you put into your achievements. Celebrate!

Do this activity today!
[If that is not possible, plan to do it on another specific day this week; Saturday at the latest.]
Afterwards, return to Get Energized to complete the activities below.

Well done on taking it all in!

Which savoring activity did you choose? How did it make you feel?
Write here…

Purposefully scheduling moments to be savored can help you put time aside to take it all in. If you have enjoyed this activity, become aware of your breath more often, and try the other two savoring activities as well!

RETURN TO HOME SCREEN

© 2025 Kókai LL et al. *JAMA Network Open*.

Week 7

Validation
An easy way to understand what validation is to think of its opposite, which is *in*validation: expressions such as “It could be worse”, “Don’t be a baby”, and “Get over yourself” are invalidating. Instead of invalidating how we feel, which can subsequently heighten intense feelings, validation can serve to soothe intense feelings. We will start with a grounding activity – this time about validating your own feelings.

Get comfortable, put on some headphones if you like, and press play below.

#Press play to hear the following text:

- Take a seat in a comfortable, quiet place. Uncross your legs and arms.
- Close your eyes, or keep a soft gaze towards the ground in front of you; lengthen your spine, and release your shoulders.
- Breathe slowly and evenly in and out through your nose.
- Once you are settled, identify an intense feeling that you have felt recently, or perhaps one that you are feeling right now – the more recent the better.
- Feel the feeling (again) with intensity, including the bodily sensations that came with it: perhaps butterflies in your stomach, lightheadedness, or fatigue.
- See if you can name what you feel. Is it anger? Anxiety? Joy? Relief? If you cannot identify the feeling, that is okay as well. Try to acknowledge this feeling, and accept it as a part of your current experience.
- Try to figure out why you are feeling this way. Be as friendly to yourself as you would be to others. If your friend was nervous before a presentation, you would remind him or her that it makes perfect sense to feel this way before speaking publicly, and that the majority of people feel this way.
- Sit with this sense of acknowledgement, acceptance, and compassion for your feelings until you hear a chime, indicating the end of this grounding activity.

#End of audio clip

Well done! Meeting our feelings heads on leads to constructive ways of moving forward.

Why do you think people sometimes suppress, rather than fully experience, their intense feelings?
Write here…

Validation can also be used on the feelings of others. Next time someone tells you about their feelings, whether about something *big* (your friend is sad after a bad review at work), *small* (your spouse is happy to have found a new TV show), or *tiny* (your mother is relieved to have gotten to the grocery store before it closed), make sure to validate their feelings.

© 2025 Kókai LL et al. *JAMA Network Open*.

Make your wording and body language *active and positive*. For example, while making eye contact, say “That sounds awfully difficult”, “It makes sense why you feel this way”, or “That really is great”.

Note that validation does not mean that you agree with or support a feeling; especially if that feeling is destructive or irrational. Validation means that you acknowledge and compassionately accept a feeling. If you have enjoyed this activity, try to use validation whenever you or another person experience intense feelings.

RETURN TO HOME SCREEN

© 2025 Kókai LL et al. *JAMA Network Open*.

Week 8

Powerful body
Welcome to the final week of Get Energized. As always, we will start with a grounding activity. We will tune into our physical being to highlight the strong connection between body and mind.

Get comfortable, put on some headphones if you like, and press play below.

#Press play to hear the following text:

- Take a seat in a comfortable, quiet place. Uncross your legs and arms.
- Close your eyes, or keep a soft gaze towards the ground in front of you; lengthen your spine, and release your shoulders.
- Breathe slowly and evenly in and out through your nose.
- Once you are settled, draw your awareness to your left foot. Hold your awareness here as you observe any sensations that might be present in this part of the body. Sensations you might observe include: tingling, contraction, swirling, heat, cold, numbness, and even absence of sensation.
- After noting whatever sensation is present in the left foot, move upwards to your left ankle. And, as you move through the body, note what arises in the mind as well. Compassionately observe if any thoughts or feelings pull your attention away from the body. Come back to your point of focus.
- Continue this process by slowly moving up the entirety of the left leg until you reach the hip. Repeat in the same way as you scan the right leg next. Again, note any tingling, contraction, swirling, heat, cold, numbness, and even absence of sensation.
- Continue upwards throughout the core of your body: the stomach, the chest and the throat. After witnessing the throat, continue from the left hand up to the left shoulder, followed by the right hand up to the right shoulder. Then, slowly move up to your head.
- Once you have completed this scan, hold your entire body in your awareness until you hear a chime, indicating the end of this grounding activity.

#End of audio clip

Well done on tuning into your physical being.

Were there certain areas of the body that held your focus more than others? If so, why was this the case?
Write here…

How you feel shapes how your body feels. But did you know that your body (language) also shapes how you feel? So stand tall, like the loving, joyful, hopeful, grateful, inspired, proud, content, and amused person you are! It will make you feel powerful.

© 2025 Kókai LL et al. *JAMA Network Open*.

You can try doing a power pose *before* meeting people.

The Performer: Take some time at home, in the elevator, or the bathroom to throw your hands in the air and widen your stance, as if you are a performer, soaking up the applause after your sold-out performance. Hold this pose for about two minutes to get the physiological changes going that will make you feel confident. Enter the meeting full of energy.

You can also do a power pose *while* meeting people.

The Wonder Woman: Puff out your chest, plant your hands on your hips, and stand with feet hip-width apart. Tilt your chin up to maximize the power trip.

Try one of the poses right now! It doesn't matter if you are not in / about to enter a social situation; you can still experience feeling confident. Next time that you could benefit from a power boost in a social situation, you will know what to do!

Which pose did you try? How did the posture of your body affect your mood?
Write here...

There is a close connection between the mind and the body. If you have enjoyed this activity, you are invited to tune in with your body and power pose whenever it feels right.

We hope that you have enjoyed the Get Energized activities of the past eight weeks! You will continue to have access to these activities until the final follow-up questionnaire of i2be 12 months from now, although the response boxes will not open up again. However, we encourage you to:

- Play the grounding audio clips again and again
- Scroll through all activities again and answer on a sheet of paper
- Discuss your answers with a friend or family member!

RETURN TO HOME SCREEN

© 2025 Kókai LL et al. *JAMA Network Open*.

eReferences

Kókai LL, Ó Ceallaigh DT, Wijtzes AI, et al. Moving from intention to behaviour: a randomised controlled trial protocol for an app-based physical activity intervention (i2be). *BMJ Open*. 2022;12(1):e053711. doi:10.1136/bmjopen-2021-053711.

Metz G, Peters G-JY, Crutzen R. Acyclic behavior change diagrams: a tool to report and analyze interventions. *Health Psychology and Behavioral Medicine*. 2022;10(1):1216-1228.

© 2025 Kókai LL et al. *JAMA Network Open*.
