# Supplementary material for: App-Based Physical Activity Intervention Among Women With Prior Hypertensive Pregnancy Disorder: A Randomized Clinical Trial
Source: JAMA Netw Open. 2025 Apr 2;8(4):e252656. doi: 10.1001/jamanetworkopen.2025.2656 (PMC11966332; doi:10.1001/jamanetworkopen.2025.2656)
Supplement: Supplement 3. — Data Sharing Statement [file jamanetwopen-e252656-s003.pdf]

Data Sharing Statement

Kókai. App-Based Physical Activity Intervention Among Women With Prior Hypertensive Pregnancy Disorder. *JAMA Netw Open*. Published April 02, 2025.
doi:10.1001/jamanetworkopen.2025.2656

Data

Additional Information: Netherlands Trial Register (NL9329)
https://onderzoekmetmensen.nl/en/trial/28866
Data available: Yes
Data types: Deidentified participant data, Data dictionary
How to access data: Available upon reasonable request from Lili Kókai (l.kokai@erasmusmc.nl)
When available: With publication

Supporting Documents

Document types: Statistical/analytic code
How to access documents: Available upon reasonable request from Lili Kókai (l.kokai@erasmusmc.nl)
When available: With publication

Additional Information

Who can access the data: Anyone, upon reasonable request
Types of analyses: Any analysis
Mechanisms of data availability: Secure file transfer (e.g. SURFfilesender)
